# A small polymerase ribozyme that can synthesize itself and its complementary strand

**DOI:** 10.1126/science.adt2760

**Published:** 2026-02-12

**Authors:** Edoardo Gianni, Samantha L. Y. Kwok, Christopher J. K. Wan, Kevin Goeij, Bryce E. Clifton, Enrico S. Colizzi, James Attwater, Philipp Holliger

**Affiliations:** 1 MRC Laboratory of Molecular Biology; Cambridge, CB1 0QH, United Kingdom; 2 Inria Centre de Lyon, team Biotic; Villeurbanne, 69100, France

## Abstract

The emergence of a chemical system capable of self-replication and evolution is a critical event in the origin of life. RNA polymerase ribozymes can replicate RNA, but their large size and structural complexity impede self-replication and preclude their spontaneous emergence. Here we describe QT45: a 45-nucleotide polymerase ribozyme, discovered from random sequence pools, that catalyzes general RNA-templated RNA synthesis using trinucleotide triphosphate (triplet) substrates in mildly alkaline eutectic ice. QT45 can synthesize both its complementary strand using a random triplet pool at 94.1% per-nucleotide fidelity, and a copy of itself using defined substrates, both with yields of ~0.2% in 72 days. The discovery of polymerase activity in a small RNA motif suggests that polymerase ribozymes are more abundant in RNA sequence space than previously thought.

The replication of genetic material, with variation on which natural selection can act, is a hallmark of life (1). How this capacity for heredity and evolution first emerged is unknown. The RNA world hypothesis posits that a catalytic RNA sequence (ribozyme) capable of driving its own replication emerged from random-sequence pools of RNA oligomers formed by prebiotic chemistry on the early Earth (2–6). This same heterogeneous pool of oligomers could be used as substrates for self-replication by the ribozyme (7, 8), starting the propagation of the genetic material within an evolving system.

To persist, such a ribozyme would need to act as an RNA-dependent RNA polymerase, catalyzing the two steps of a self-replication cycle — the synthesis of its complementary strand ((-) strand) and of itself ((+) strand) — using monomer or short oligomer substrates that allow the free sequence variation needed for open-ended evolution. A single RNA molecule would need to fulfil the multiple interdependent functions of such an RNA polymerase: general template and substrate binding, iterative phosphodiester bond formation, and accurate RNA-templated RNA synthesis. Ultimately, performing these functions with sufficient yields and fidelity to overcome chemical and mutational decay would allow the propagation of genetic information (6, 9).

RNA sequences that catalyze RNA-templated RNA synthesis using mono- or trinucleotide triphosphate substrates have been discovered by *in vitro* evolution and RNA engineering of the class I ligase ribozyme (class I polymerases) (10–18). However, while these polymerase ribozymes show how RNA can fulfil many of the functions required for self-replication, they have so far fallen short of self-replication, unable to synthesize even their (+) or (-) strands individually. This may be due to both their size (~150-300 nucleotides), which imposes a high synthetic burden, and their structural complexity, as stably folded RNA can pose a significant obstacle to replication (13, 14).

The size and complexity of the class I polymerases not only reflect their descent by augmentation of the already large class I ligase ribozyme (11, 13), but are also in accord with the notion that functional sophistication in RNA scales with size and structural complexity (19–21). However, the large size of these ribozymes challenges the validity of the RNA World hypothesis, due to the implausibility of their spontaneous emergence (22–24). Furthermore, RNA sequences of the size of existing polymerase ribozymes are far outside the range of RNA oligomers observed to form abiotically (25–29). This leads to seemingly paradoxical requirements for RNA self-replication, whereby ribozymes must be large and complex to encode polymerase activity, but that same size and complexity impede both their self-replication and initial emergence.

## Discovery of new small ribozyme motifs

To reconcile the two competing requirements of short length and complex activity, we hypothesized that RNA polymerase activity might be encoded by shorter RNA motifs. This would be advantageous for both self-replication and its emergence, as shorter sequences would be both easier to copy and more readily generated by prebiotic chemistry. To define a minimal length of RNA sequence required for polymerase ribozyme function, we carried out a *de novo* selection from a random sequence pool for the templated polymerization of RNA. To ease the adaptive demands and isolate the shortest motif possible, we leveraged our past observations that RNA polymerase activity is boosted in eutectic ice, due to its capacity to both stabilize polymerase ribozymes and concentrate their substrates (30). We chose trinucleotide triphosphates (henceforth called triplets) as substrates, as they have been shown to both enable the copying of structured RNA templates (13, 31) and inhibit strand reannealing in replication cycles (32). Triplet substrates thus minimize the phenotypic requirements of a polymerase without compromising the free sequence variation in synthetic products, a prerequisite for open-ended evolution in RNA self-replication.

**Fig. 1:**
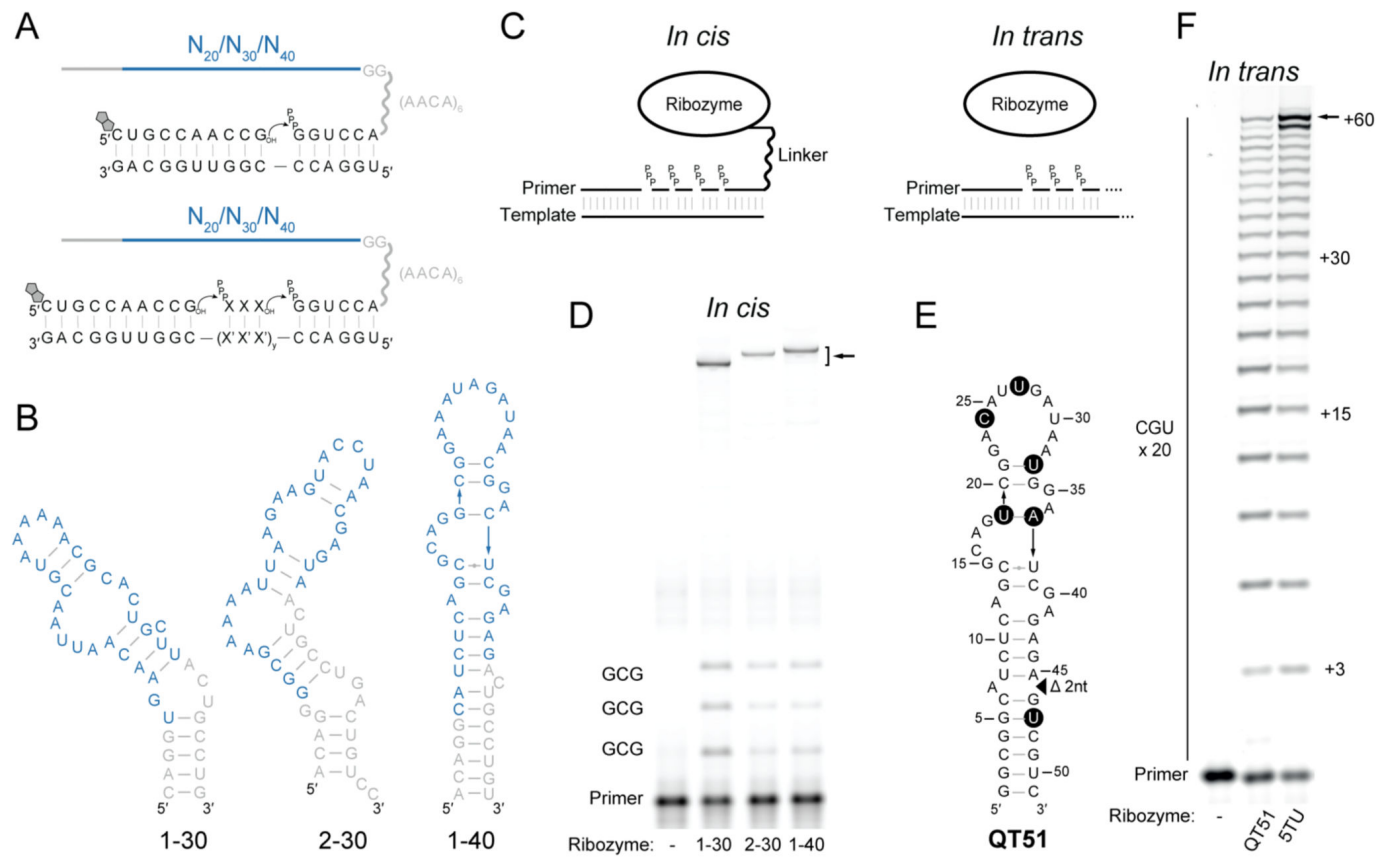
Discovery and evolution of three small polymerase ribozyme motifs. (**A**) *Top*: selection construct used for the initial selection rounds (rounds 1-3/5), with the library tethered via a flexible linker to a hexamer sequence complementary to the template 5′ end. The 5′-biotinylated primer enables the capture of active ribozymes (details in fig. S1-S2). *Bottom*: selection construct used in the later rounds (3/5-11), requiring the polymerization of triphosphorylated trinucleotide (triplet) substrates. Sequence of the triplet (XXX) and number of triplets (y) encoded by the template (X′X′X′) was varied over the course of the selection (details in table S1-S2). (**B**) Sequence and predicted secondary structures of three RNA motifs that display iterative triplet ligation i.e. triplet polymerase activity (in blue: nucleotides derived from the random library section, in gray: nucleotides derived from the constant flanking regions). (**C**) Two types of ribozyme activity. *In cis*: ribozyme is hybridized to template via a flexible linker to a hexameric tag that base pairs to the template, favoring a pseudo-intramolecular reaction. *In trans*: ribozyme interacts freely with the primer-template and polymerizes triplets in an intermolecular reaction. (**D**) Iterative triplet (x3 pppGCG) polymerization by the ribozymes displayed in (B) assayed using the *in cis* format. Arrow indicates region where the *in cis* construct migrates when ligated to the full-length product. Reaction conditions: 50 nM ribozyme-substrate, 50 nM primer BCy3P10GA, 50 nM template t6FP10gaGCG3, 5 μM pppGCG triplet, 0.05% Tween 20, 200 mM KCl, 50 mM MgCl_2_, 50 mM CHES-KOH, pH 9, -7 °C frozen, 3 days. (**E**) Sequence and predicted secondary structure of the QT51 ribozyme derived from 1-40 motif by mutation of 6 residues (black circles) and a 2-nucleotide deletion (triangle). (**F**) Synthesis of a 60-nucleotide sequence using the *in trans* format in comparison with 5TU polymerase ribozyme. Reaction conditions: 0.25 μM primer F10, 0.25 μM template tP10CGU20, 0.25 μM ribozyme (QT51 or 5TU+t1.5), 10 μM pppCGU triplet, in 0.05% Tween 20, 50 mM MgCl_2_, 50 mM CHES-KOH, pH 9 for QT51, and in 0.05% Tween 20, 200 mM MgCl_2_, 50 mM Tris-Cl, pH 8.3 for 5TU+t1.5, both incubated at -7 °C frozen, 14 days.

We initiated selections from three small, random sequence pools (~6×10^12^ unique RNA sequences) each containing a short (20, 30 or 40 nucleotides) randomized region constituted as a tandem repeat (fig. S1). We challenged the pools first to catalyze a single templated ligation of a primer to an adjacent triphosphorylated substrate that is covalently linked to the library via a flexible RNA linker (Fig. 1A, top). Once catalytic activity was observed, we challenged the pools to catalyze triplet polymerization (Fig. 1A, bottom). Owing to the flexible linker, active members of the pools preferentially react intramolecularly, extending the primer and ligating it to themselves via the tethered substrate. This results in a covalent coupling between active members of the library and the biotinylated primer, enabling selective recovery of active library members via streptavidin pull-down. To ensure generality of polymerase activity, selective pressure was gradually increased by requiring the templated incorporation of an increasing number of triplets and varying the template and primer sequences used throughout the rounds (table S1).

We had initially designed our library construct as a tandem repeat based on the reasoning that a larger, dimeric RNA would have a greater chance to form a complex ribozyme structure, while requiring only the monomer sequence to be replicated. However, when we assayed for polyclonal polymerase ribozyme activity in monomeric and dimeric form, activity was observed in the library even in its monomer form in round 8 (fig. S2). Further rounds of selection were therefore carried out using the monomer form (fig. S3). After 11 rounds of selection, we identified three small, unrelated RNA motifs from two of the libraries (named 1-30, 2-30, 1-40) (Fig. 1B) each with template-dependent RNA polymerase ribozyme activity satisfying the minimum requirements of iterative, cognate triplet addition for different template sequences (Fig 1D, fig. S4). All three motifs displayed regiospecific formation of the canonical 3′-5′ phosphodiester bond (fig. S5), similarly to the class I ligase (33).

## QT: a new small ribozyme class with complex RNA polymerase activity

Each isolated clone was subjected to mutagenesis (24% per base randomization) and 7 more rounds of selection (table S2), resulting in a dominant clone with robust triplet polymerase activity derived from the 1-40 ribozyme (Fig. 1E). This 51-nucleotide ribozyme (named “Quite Tiny 51” – QT51) catalyzes RNA-templated phosphodiester bond formation from a 3′-OH and an adjacent 5′-triphosphate (5′-PPP) at an apparent rate (k_obs_) of 0.06 min^-1^ (fig. S6) and can synthesize RNA sequences longer than itself, such as a 60-nt repeat of 20 CGU triplets (yield: 3%, in 14 days) (Fig. 1F). This compares favorably with the previously discovered class I RNA polymerase ribozyme 5TU+t1.5 (hereby referred to as 5TU) that uses triplet substrates (13, 31). 5TU is more than 5 times larger than QT51 (fig. S7) and serves as a standard for triplet polymerization throughout this study.

**Fig. 2:**
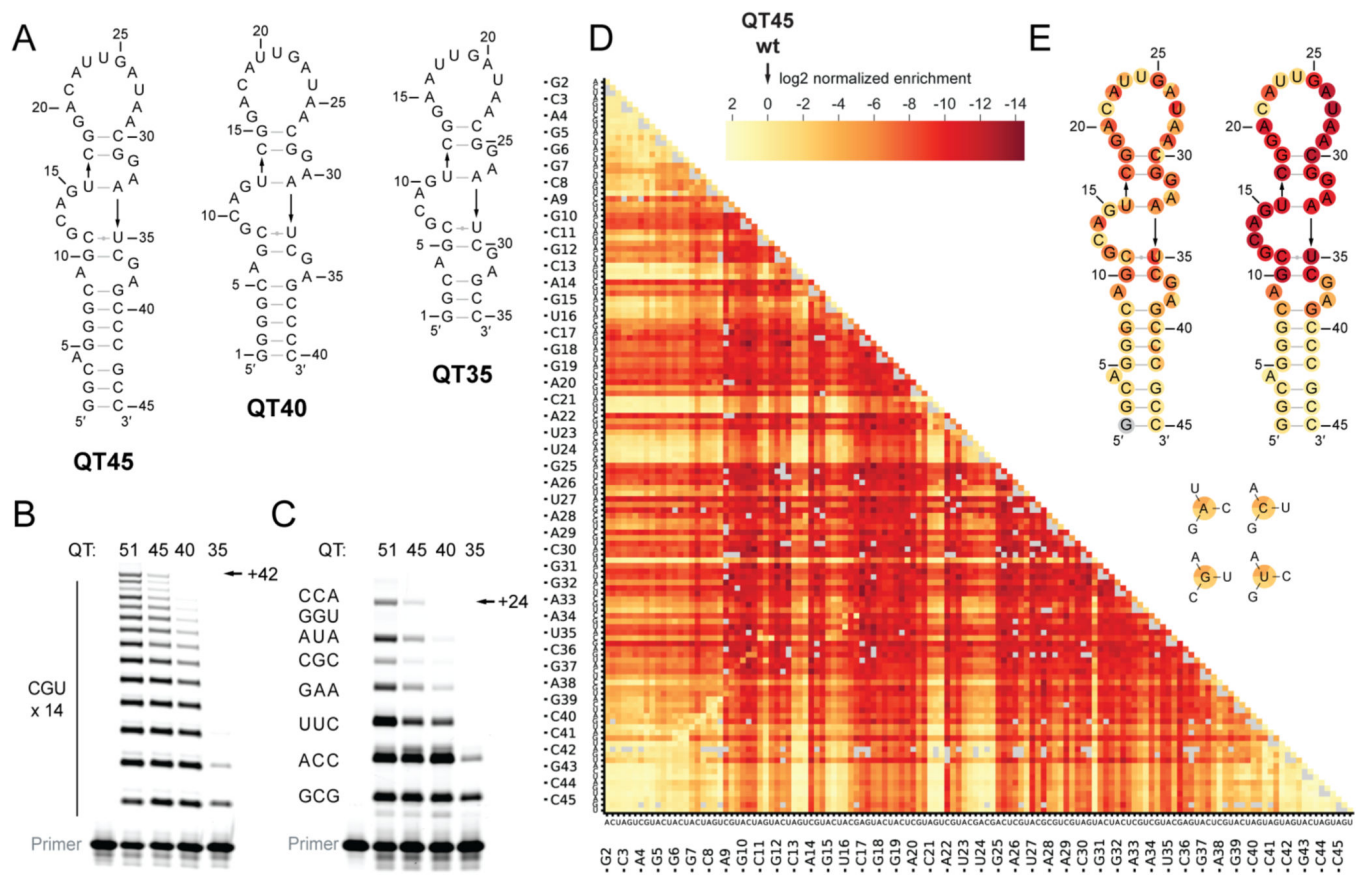
A small RNA motif with a functionally dense core encodes the triplet polymerase activity of the QT ribozyme. (**A**) Predicted secondary structure diagrams of the ribozymes used in (B) and (C). (**B**) Synthesis of a 42-nucleotide CGU repeat sequence by QT51 and its truncation variants. Reaction conditions: 0.25 μM primer F10, 0.25 μM template tP10CGU14, 0.25 μM ribozyme, 5 μM pppCGU triplet, 0.05% Tween 20, 50 mM MgCl_2_, 50 mM CHES-KOH, pH 9, -7 °C frozen, 5 days. (**C**) Synthesis of a mixed sequence template by the same ribozymes as in (A). Reaction conditions: 0.25 μM primer F10, 0.25 μM template t6FP10mix, 1.25 μM ribozyme, 5 μM each triplet, 0.05% Tween 20, 50 mM MgCl_2_, 50 mM CHES-KOH, pH 9, 5 days at -7 °C frozen. In both (A) and (B), ribozymes are not hybridized to template. (**D**) Heatmap of log normalized enrichment for primer extension activity on a 3 UGC template by measured single mutants (132 mutants) and double mutants (8346 mutants) of the QT45 ribozyme, with the first constituent point mutation indicated on the x-axis and the second mutation on the y-axis. Missing data points are shown in gray. (**E**) Predicted secondary structure of QT45 ribozyme with nucleotide color corresponding to measured activity on a 3 UGC template for (*left*) each of three possible single substitutions at each position or (*right*) a deletion at each position. Substitution mutations displayed in the same order as the three representative mutations shown in the key below. Nucleotide colors use the same color scheme as panel (D).

Polymerase ribozyme activity persisted in progressively truncated (45, 40, 35 nucleotides) versions of QT51 (Fig. 2A). The 45-nucleotide version, named QT45, retained near full RNA polymerase activity (as judged by synthesis of a 42-nucleotide product), whereas the further truncation variants QT40 and QT35 showed progressively reduced activity (Fig. 2B). The small size of QT45 also results in enhanced stability to degradation, with a half-life of ~117 days in standard reaction conditions (fig. S8), compared with the larger class I ligase derived R18 ribozyme, displaying a half-life of ~16 days (30).

To test its general RNA polymerase activity, we challenged QT51 and its truncation variants to copy a 24-nucleotide mixed sequence template comprising a representative variety of triplet junctions (8 of 16 possible ones). Both QT51 and its QT45 truncation could successively incorporate the 8 varying triplets required to synthesize this sequence (Fig. 2C). We also observed that QT ribozyme polymerase activity is not limited to trinucleotide triphosphate substrates but extends to the incorporation of longer oligonucleotides and, to a lesser extent, dinucleotide and mononucleotide triphosphates (fig. S9, fig. S10). QT45 polymerase activity is retained in the presence of triphosphorylated substrate pools composed of mono- (pppN), di- (pppNN) and trinucleotides (pppNNN) at varying ratios, with some reduced product yields at higher mono- and dinucleotide concentrations on longer templates (fig. S11). Furthermore, QT ribozyme is able to utilize 5′-adenylated (5′-5′-pyrophosphate-linked) oligonucleotide and triplet (AppGCA) substrates, although with reduced efficiency (fig. S10). These are major side products (and inhibitors) of imidazole-based activation chemistries for nonenzymatic RNA polymerization (34–36), and thus potentially a more prebiotic alternative (or precursor) to triphosphate substrates. Such promiscuity in substrate length and activation chemistry might have benefited RNA replication in a heterogeneous prebiotic environment (37).

Although originally selected linked to its substrate, QT ribozymes do not require any tethering or base pairing to the primer–template–substrate complex for RNA polymerase activity (Fig. 1C, 1F) and show multi-turnover activity (fig. S12). This suggests that the QT ribozyme can engage with the primer–template–substrate complex purely via general, sequence-independent, tertiary contacts. This advanced phenotype was previously only observed in the more advanced versions of the much larger class I polymerases (11, 13, 31, 38, 39), or in a cross-chiral ribozyme polymerase (40, 41).

In order to assess the QT45 ribozyme tertiary interactions with the primer–template-substrate, we mapped the location of the 2′-OH groups needed for efficient catalysis five nucleotides upstream and downstream of a model ligation junction (-5, 0, +5) by 2′-deoxynucleotide substitution (as in (38, 42)), and compared it to the 5TU ribozyme. Suppression of ligation by 2′-deoxy-substitutions was observed for both QT45 and 5TU at positions -4 and +2 (in the template strand) and positions -1, -2 and -3 for QT45, and positions -1 and -2 for 5TU (in the primer strand). For the substrate strand, significant suppression was observed at positions +3 and +4 for QT45, and positions +1 to +4 for 5TU (fig. S13). The close analogy between tertiary contacts for QT45 and the unrelated 5TU ribozyme is unexpected and may suggest convergent evolution towards a common mode of primer-template engagement for polymerase ribozymes.

To further understand the sequence determinants of polymerase function in this small RNA motif, we performed a comprehensive fitness landscape analysis on QT45, similarly to a previous analysis on 5TU (31) (see Supplementary Text for details on the analysis). We quantified genotype abundance changes after a single round of selection for triplet polymerase activity on a template encoding 3 UGC triplets, which provided fitness estimates for all QT45 single mutants, single deletions, and 98% of double mutants (Fig. 2D, Fig. 2E). The selection was conducted in triplicate, with fitness values shown to have strong correlation (fig. S14). This fitness landscape analysis revealed a sharp fitness peak with most mutations being detrimental to activity. QT45 single mutants ranged from -11.0 to 1.6 fitness, and double mutants from -14.5 to 2.4, which is much more negative than the 5TU fitness landscape (23) which ranges from -4.6 to 1.6 for single mutants and -8.0 to 2.9 for double mutants (fig. S15). The fitness landscape and single nucleotide deletion analysis revealed the core functional architecture of QT45: most core positions were intolerant of deletions while the basal stem region was more permissive (Fig. 2E), especially when base pairing was maintained. These patterns were consistently observed across different selection templates (fig. S16-18) and in a lower resolution fitness analysis for QT39, a variant maintaining the same core with a truncated stem (fig. S19).

Epistasis analysis revealed predominantly negative epistatic interactions, but less negatively biased than in 5TU (fig. S20). Together, fitness values and epistatic interactions (table S3-5) provided evidence for canonical base pairs (G10-C36, U16-A34, G18-C30, C8-G39, G7-C40, G6-C41) and non-canonical pairing (C11-U35), informing the prediction of QT45’s secondary structure (fig. S21). This analysis also uncovered critical sequence requirements in the apical loop and basal stem (fig. S22). The low tolerance for mutations and deletions of QT45 likely reflects a high density of functional residues in the QT ribozyme core required for sustaining the multiple structural and functional requirements of a polymerase ribozyme in a small RNA motif.

Based on the analysis of core residue identity from the fitness landscape mapping we estimate the abundance of QT ribozyme folds from random sequence pools as 9.5 × 10^-17^ (see Supplementary Text for details on this calculation). The isolation of the QT ribozyme and two other polymerase motifs from a starting pool of only ~1.2 × 10^13^ sequences points to a potentially much higher abundance of polymerase motifs in RNA sequence space, as only a small portion of the 1.2 × 10^24^ possible N40 sequences was sampled.

## Ribozyme-catalyzed synthesis of an active ribozyme

**Fig. 3:**
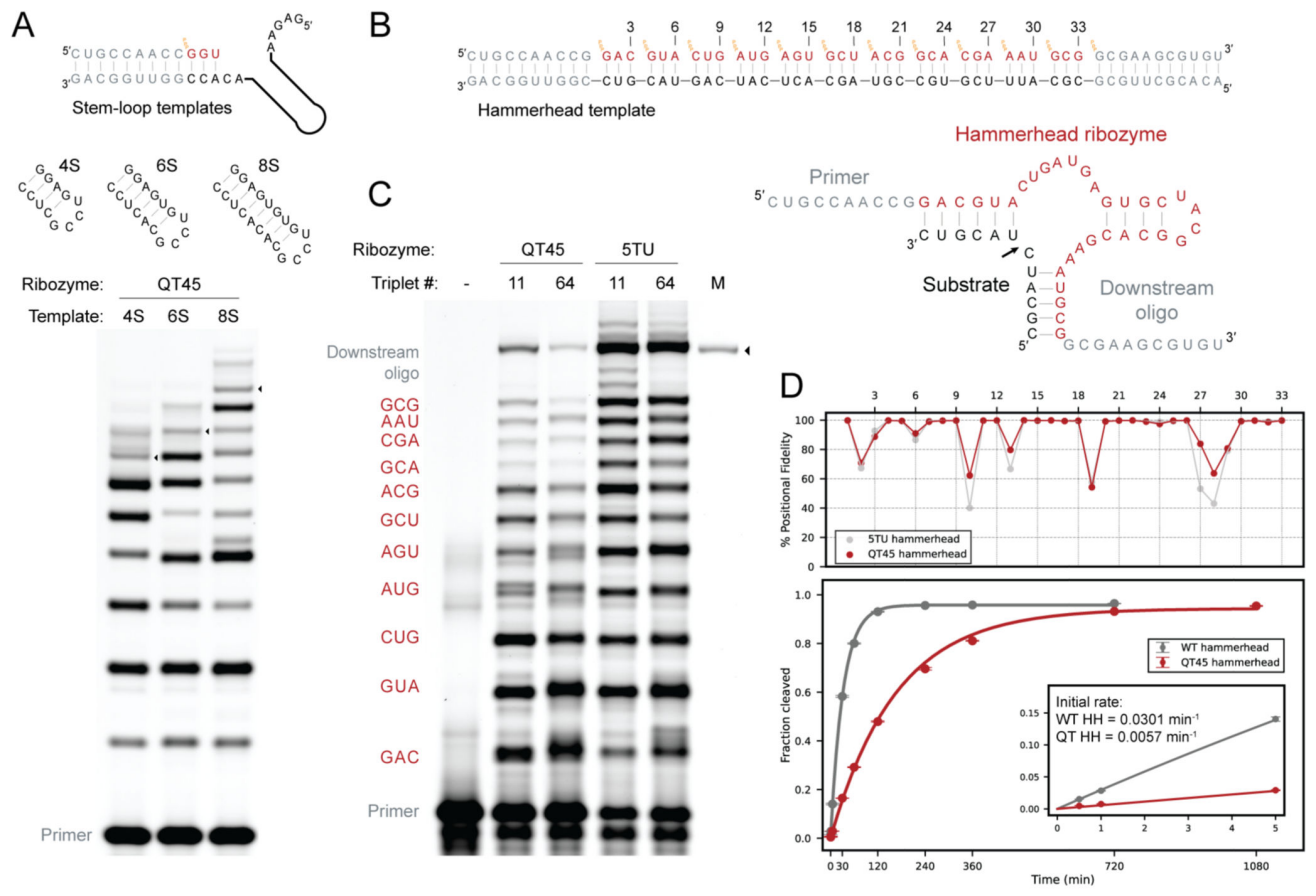
QT45 ribozyme-catalyzed synthesis of structured sequences and of an active ribozyme. (**A**) RNA synthesis on three stem-loop-containing templates with increasingly stable secondary structures. Predicted stem-loop secondary structures displayed on top. Primer extension reaction shown below, with black triangles indicating full-length product migration. Reaction conditions: 0.25 μM primer (F9), 0.25 μM template (t4S/t6S/t8S, indicated above each lane), 1.25 μM QT45 ribozyme, 5 μM each triplet (see fig. S23 for details), 0.05% Tween 20, 50 mM MgCl_2_, 50 mM CHES-KOH, pH 9, -7 °C frozen for 35 days. (**B**) *Top*: diagram of primer, template and triplets necessary for the synthesis of the hammerhead ribozyme. *Bottom*: diagram of the hammerhead ribozyme (red) in complex with its substrate (black). The cleavage site is indicated by an arrow. (**C**) Ribozyme-catalyzed synthesis of the seq0-HH hammerhead ribozyme. Full-length product indicated by a black triangle. M indicates marker full-length product lane. Reaction conditions: 0.25 μM primer BCy3P10, 0.25 μM template tP10Lte_seq0HH, 2.5 μM QT45 or 0.25 μM 5TU+t1.5, 1.25 μM each defined triplet or each of the 64 possible triplets (NNN), 0.25 μM downstream oligo pppLtest1, 0.05% Tween 20, 50 mM MgCl_2_, 50 mM CHES-KOH, pH 9, 65 days at -7 °C frozen. (**D**) *Top*: Positional fidelity of copying by QT45 shown in red, 5TU control shown in grey. *Bottom*: time-course of the cleavage activity of hammerhead ribozyme sequences synthesized by QT45 (red), compared with perfect sequence controls (gray). The data were acquired in triplicates and were fit to a single exponential function (solid lines; R^2^ ≥ 0.999), (see methods). The inset expands on the initial time points. Initial rate showed 0.0301 ± 0.0001 per minute for WT seq0-HH, and 0.00570 ± 0.00001 per minute for QT45-derived seq0-HH (18.9 ± 0.1% of wild-type initial rate).

To further examine the general polymerase activity of the QT45 ribozyme, we tested its ability to copy increasingly structured stem-loop templates (4-, 6-, 8-bp stems). In all three cases QT45 could synthesize the reverse complement of the stem-loop structure and yield full-length product (Fig. 3A), with improved yields at higher triplet concentrations (fig. S23). Such an ability to copy structured RNA templates is important for general RNA replication activity and was previously only observed in the much larger, more complex and more evolved triplet polymerase ribozyme t5^+1^ (13) and mononucleotide polymerase ribozyme 24-3 and their descendants (14).

The ability to copy structured sequences is critical for the synthesis of functional RNAs, whose templates often encode structured segments required for folding into a functional shape. To test this, we challenged QT45 to synthesize seq0-HH, a 33-nt hammerhead endonuclease ribozyme sequence (Fig. 3B), previously used to characterize polymerase ribozyme fidelity (39). This hammerhead ribozyme version is of a useful size and complexity for benchmarking polymerase ribozyme activity, although it dispenses with some sequence elements needed for full activity in cells (43, 44). QT45 was able to synthesize the full length seq0-HH using both the 11 defined triplet substrates as well as a random substrate pool comprising all 64 triplets (NNN), with yields of 1.88% and 0.39% respectively after 65 days (Fig. 3C).

We verified the identity of the synthesized products by deep sequencing, yielding 42.2% reads of perfect full-length seq0-HH products using defined triplets, and 8.5% using a random triplet substrate pool. From this, we estimate the average per nucleotide fidelity for full-length product synthesis by QT45 to be 92.6%, slightly higher than that of the most highly evolved mononucleotide polymerase ribozyme 71-89 (90.9%) on the reverse complement sequence of the same seq0-HH sequence. 5TU fidelity (89.5%) is lower than expected (given the 97.4% average per nucleotide fidelity measured for the closely related t5^+1^ ribozyme), but this may be due to the different pH of the assay conditions from those in which 5TU had been evolved (CHES-KOH pH 9 instead of Tris-HCl pH 8.3). G:U wobble mispairing, particularly at the first triplet position, is the main source of errors for all of these polymerase ribozymes (Fig. 3D).

While full-length product fidelity is the most relevant parameter for functional ribozyme synthesis, intermediate product fidelity may be informative in the context of identifying problematic sequence motifs that cause stalling and misincorporation during synthesis. In order to assess this, we sequenced all intermediate products of the seq0-HH synthesis. In some of the products we observed a reduction of fidelity for the terminal triplet (fig. S24). This is consistent with a previously described purifying effect of stalling of synthesis caused by misincorporation, leading to an improved overall fidelity of full-length products at the expense of synthetic yield (45).

We validated the functionality of seq0-HH sequences synthesized by QT45 with the random-triplet mix by comparing the RNA endonuclease activity of wild-type seq0-HH to that of seq0-HH sequences (derived from RT-PCR and transcription of the recovered QT45-synthesized full-length products). QT45-derived seq0-HH sequences were catalytically active and showed ~19% wildtype activity in the initial phase of the reaction (Fig. 3D, bottom). The directly isolated RNA products of a mini-hammerhead ribozyme fragment (46) synthesized by the QT ribozyme also exhibited endonuclease activity (Fig. S25). Ribozyme-catalyzed synthesis of functional RNAs (ribozyme or aptamer) would have underpinned any primordial RNA metabolism (6), and had previously only been achieved by the much larger class I polymerase ribozymes (12–14). This demonstrates that QT45 has the catalytic capabilities required for the synthesis of ribozymes approaching the size and complexity of itself.

## Ribozyme-catalyzed synthesis of its complementary strand and of itself

**Fig. 4:**
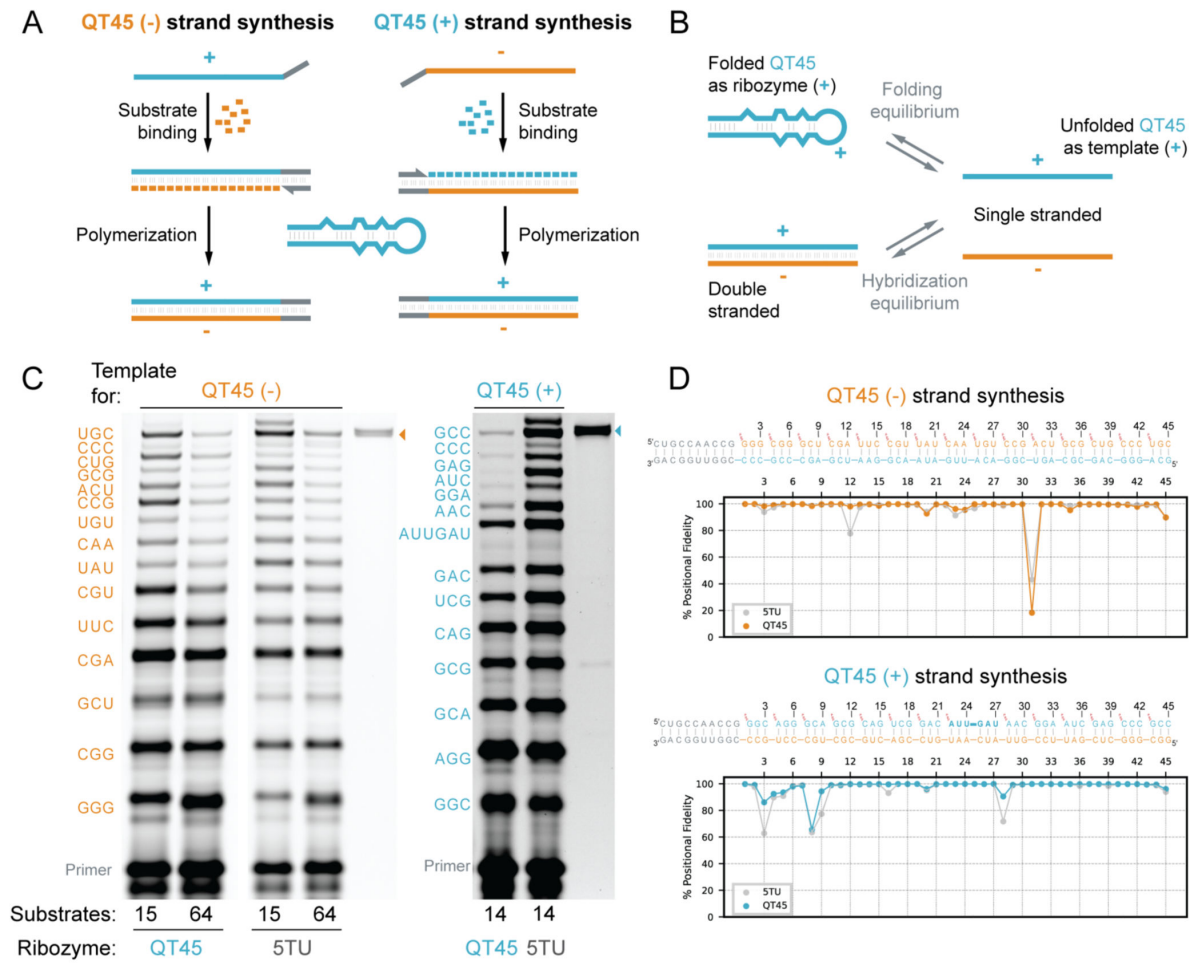
QT ribozyme-catalyzed synthesis of its complementary strand and of itself. (**A**) Diagram of the two component reactions of an RNA self-replication cycle tested with QT45: synthesis of (-) strand, the reverse complement of the ribozyme, and synthesis of the (+) strand, corresponding to the ribozyme. A primer binding site external to the ribozyme sequence is included in the template to facilitate recovery and detection of the synthetic products. (**B**) Diagram of the two key challenges specific to RNA self-replication: (+) strand must exist in an equilibrium between folded as ribozyme and unfolded as template, (+) and (-) strands need to be separated and remain in single stranded format for productive self-replication. (**C**) *Left*: QT45-catalyzed synthesis of its own complementary strand (QT45(-)) starting from 15 defined triplet substrates or all 64 possible triplet substrates, compared with the 5TU-catalyzed synthesis of the same sequence. Full-length product indicated by a tan triangle. *Right*: QT45-catalyzed synthesis of itself (QT45(+)) using a mix of triplet substrates with the aid of one pre-formed hexamer. Full-length product indicated by a teal triangle. Reaction conditions for both syntheses: 8 nM primer BCy3P10, 8 nM template (t4psP10QT45 or t4msP10QT45), 80 nM ribozyme QT45 (pQT45 for (+) strand) or 5TU+t1.5, 50 nM each substrate, 0.01% Tween 20, 0.4 mM MgCl_2_, 1.2 mM KCl, 1 mM CHES-KOH, pH 9, incubated for 72 days at -7 °C frozen. QT45(+) synthesis reaction underwent one pH-heat-freeze cycle to favor strand separation. (**D**) Positional fidelity of copying of full-length QT45(-) or full-length QT45(+), with QT45-catalysed synthesis shown in tan/teal, 5TU control shown in gray.

The robust and general triplet polymerase activity of the QT45 ribozyme, combined with its small size, suggested that it might meet the critical synthetic requirements for self-replication: the templated synthesis of both a ribozyme’s template strand (-) and of its own (+) strand (Fig. 4A).

To test this, we first examined the QT45-catalyzed synthesis of its own complementary (-) strand, wherein QT45 (the (+) strand) acts both as the template strand and as the catalyst. This requires the reconciliation of two seemingly paradoxical requirements: within the same reaction, QT45 must be both folded — to act as a catalyst — and unfolded — to act as a template. These conflicting requirements can be reconciled within the behavior of an RNA ensemble: two RNA conformations are present simultaneously within the ensemble, through a folding equilibrium (Fig. 4B). As the triplet substrates bind and unfold the template strand, this equilibrium is sensitive to the triplet concentration (fig. S26A), as observed for templates containing secondary structures (Fig. 3A, fig. S23) (13). At higher triplet concentrations, the unfolded QT45 (+) template strand conformation will be increasingly populated as triplets cooperatively bind to the (+) strand, consistent with the inhibition of QT45 by high triplet concentrations (fig. S27). At lower triplet concentrations, the folded, active QT45 (+) strand ribozyme conformation predominates. We therefore first performed a series of reactions to define the optimal range of triplet and ribozyme concentrations (fig. S28). Using these optimized conditions, we observed synthesis of the full-length complementary (-) strand when providing the defined 15 triplet substrates required for the synthesis or the complete pool of 64 triplet (pppNNN) substrates (yield: 0.24% after 72 days) (Fig. 4C). Deep sequencing confirmed the identity of the full-length (-) strand synthesized using the NNN pool, with 10.9% perfect full-length (-) strand sequences, and an average per nucleotide fidelity of 94.1% (Fig. 4D), slightly higher than the fidelity of seq0-HH synthesis.

Having shown that QT45 could synthesize its own QT45 (-) template strand, we next sought to test if it could also synthesize itself, i.e. another QT45 (+) strand from a QT45 (-) template. This again requires reconciliation of conflicting constraints and trade-offs. Here, the (+) strand must fold (and remain folded) into a catalytic RNA motif and interact with the complementary (-) strand template without forming the thermodynamically highly favored (+)(-) strand duplex, which is an inactive, dead-end product (Fig. 4B). Again, we sought to resolve this conflict by exploiting the ability of triplets to shift the folding and duplex-formation equilibrium to kinetically trap the QT45 (-) template in an unfolded template conformation stabilized by triplet binding (fig. S26B).

One might assume that simply providing an excess of QT45 (+) ribozyme over QT45 (-) template should maximize synthetic yields. However, while this can improve the yields of partial products, we observed consistent inhibition of extension before reaching full-length QT45 (+) strand synthesis (fig. S29). Inhibition was even observed when supplementing the reaction with the unrelated 5TU polymerase ribozyme to serve as the catalyst, pointing towards sequestration of the template (-) strands by the QT45 (+) strands independently of the catalyst used. We conjecture that, at higher concentrations, the (+) strand outcompetes triplet substrates for binding to the (-) strand template, driving increasing formation of the unproductive (+)(-) strand duplex (fig. S26B). Indeed, in the absence of the QT45 (+) strand, we observed efficient full-length synthesis by 5TU. This suggests that the strand-inhibition problem, caused by the formation of the dead end (+)(-) strand duplex, is a key obstacle to closing the self-replication cycle.

We hypothesized that substrates that interact more strongly with the template strand might compete more effectively with QT45 (+) for hybridization to the QT45 (-) template. To test this, we performed a series of reactions each supplemented with a single triphosphorylated RNA hexamer substrate (equivalent to two pre-ligated triplets) complementary to different template positions. Among these, hexamers binding to the template region for the AU-rich apical loop (G25-C30, or A22-U27) proved effective at relieving strand-inhibition and supporting full-length synthesis (fig. S30, fig. S29, Fig. 4C). Therefore, the inclusion of one defined RNA hexamer (pppAUUGAU) together with the required triplet substrates enabled self-synthesis of the full length QT45 (+) strand by the QT45 (+) ribozyme on a QT45 (-) template (yield: 0.17%, in 72 days) (Fig. 4C). Deep sequencing confirmed the synthesis of the correct QT45 (+) sequence from defined substrates (Fig. 4D) with 43.4% of perfect full-length QT45 (+) products.

These results show that the two key synthetic reactions required for a self-replication cycle: (-) and (+) strand self-synthesis, can both be carried out by the QT45 ribozyme in separate reactions under identical buffer, salt, and RNA concentrations. These reaction conditions were optimized to match the low RNA and MgCl_2_ concentrations recently found to enable RNA replication via iterative coupled pH/freeze-thaw cycles (32). Using the same conditions, we tested QT45 for replication of a model double-stranded RNA template and show that QT45 can copy two complementary RNA sequences simultaneously in a single “one-pot” reaction using pH-freeze-thaw cycles (fig. S31).

Using a random pool of all possible 64 trinucleotide substrates enables mutation and free sequence variation during replication, which is a prerequisite for evolution. However, the number of mutations per round of replication (error rate) and the relative replication fitness of these mutants define the maximum sequence length that can be sustained (Eigen error threshold) (6, 9, 47). By this measure, the short length of QT45 would be predicted to be advantageous, as it reduces the fidelity required for survival compared to the larger polymerase ribozymes (fig. S32A). To better understand replication in the QT45 system, we modelled the specific behavior of a population of QT45 wild-type and its mutants using the empirical relative fitness values measured in this study (Fig. 2D) as proxies for replication rates (see Supplementary Text for details on the model). This analysis suggests that at the fidelities observed for QT45 products (92.6% and 94.1%) the population of active variants (quasispecies) would be minimal (<0.5%), but an increase in fidelity to levels observed in other polymerase ribozymes (e.g. 97.4% (13)), would enable maintenance of a sizeable population of active variants of QT45 length in the current mutational landscape (~12% of the total population, fig. S32B).

We also observed a low background of full-length product formation independent of triplet substrates, without the characteristic extension ladder of *bona fide* ribozyme synthesis products (fig. S30). This side product arises from recombination by transesterification between partially extended products and the ribozyme (fig. S33A), via nonenzymatic ligation of a 2′,3′-cyclic phosphate to an adjacent 5′-hydroxyl as observed previously (48–51) and reproduced in our work (fig. S33B). In order to discern the synthetic products (produced by ribozyme catalysis) from the recombination products, we appended a short DNA tail to the QT45 ribozyme sequence, without including this sequence in the template encoding it (fig. S34). This results in different migration of the synthetic (no tail) products compared to the recombined (with tail) products in gel electrophoresis, allowing us to separate the two products for sequencing reactions. While recombination here is an inconvenient side reaction for analytical purposes, the inherent property of RNA to recombine is likely to be advantageous in self-replication reactions as recombination is known to accelerate evolution and mitigate the deleterious effects of mutational drift (Muller’s ratchet) (52–54).

## Discussion

Our study shows that the complex functions needed for RNA replication — intermolecular binding to the primer-template-substrate complex, regiospecific catalysis, and general template-dependent RNA synthesis — can all be performed by an RNA motif of just 45 nucleotides. This advanced phenotype, encoded in a small motif, enables QT45 to achieve the two key reactions required for self-replication: the synthesis of its complementary strand and of itself. These syntheses are currently slow and low-yielding, with further improvements in synthetic efficiency and fidelity likely required to overcome chemical degradation and achieve self-sustained replication. However, the QT ribozyme has only undergone a total of 18 rounds of evolution from a random sequence pool, indicating a likely potential for further development.

Our experiments revealed two conflicting requirements inherent to RNA self-replication: the need for ribozymes to simultaneously coexist as both folded catalysts and unfolded templates and to avoid inhibition of the ribozyme activity by hybridization to its complementary (template) strand. The QT ribozyme-catalyzed synthesis of itself and of its complementary strand shows how these constraints can be overcome through the emergent properties of RNA ensemble equilibria and their modulation by triplet substrate interactions and reaction conditions. Together with recent advances in our understanding of the physico-chemical conditions conducive to RNA replication (32, 55–57) this suggests a plausible path towards iterative cycles of RNA-catalyzed RNA self-replication.

The small size of the QT polymerase ribozyme, approaching lengths that may be accessible by nonenzymatic RNA polymerization (25, 26), reduces the synthetic and fidelity burden for self-replication, enhances ribozyme half-life, and suggests that motifs encoding such an activity may be more abundant in RNA sequence space than anticipated.

## Supplementary Material

Supplementary Materials

## Data Availability

sequencing datasets generated in this study and processing pipelines are available at Dryad (58), processed datasets, code to analyze the sequencing datasets, and the quasispecies model are available on Zenodo (59). All other data are available in the main text or the supplementary materials. All materials used in this study will be made available upon request.
