# Supplementary material for: A small polymerase ribozyme that can synthesize itself and its complementary strand
Source: Science. Author manuscript; Available in PMC 2026 Feb 24. (PMC7618777; doi:10.1126/science.adt2760)
Supplement: Supplementary Materials [file EMS212593-supplement-Supplementary_Materials.pdf]

Supplementary Materials for

A small polymerase ribozyme that can synthesize itself and its complementary strand

Edoardo Gianni*, Samantha L. Y. Kwok, Christopher J. K. Wan, Kevin Goeij, Bryce E. Clifton, Enrico S. Colizzi, James Attwater, Philipp Holliger*

*Corresponding authors: Edoardo Gianni, egianni@mrc-lmb.cam.ac.uk; Philipp Holliger, ph@mrc-lmb.cam.ac.uk

The PDF file includes:

Materials and Methods
Supplementary Text
Figs. S1 to S34
Tables S1 to S8

Methods and Materials

1. Various nucleic acid manipulations

Methods repeatedly used in the paper have been collated for convenience. Variations from standard protocols are otherwise stated *in itinere.*

1.1. T7 *in vitro* transcription

The *in vitro* transcription method used is based on (*60*). If the RNA required a triphosphate at the 5′-end, the "GTP" transcription protocol was used. If the RNA required a monophosphate at the 5′-end, the "GMP" transcription protocol was used. "GTP" transcription reaction conditions: 40 mM Tris·HCl pH 8, 10 mM DTT, 2 mM spermidine, 20 mM $MgCl_2$, 7.5 mM each NTP (Thermo Fisher Scientific), double-stranded DNA template containing 5T7 sequence at the 5′ end upstream of the region to transcribe (varying amount, preferably >5 pmoles), 0.01 units/μL of inorganic pyrophosphatase (Thermo Fisher Scientific), ~50 μg/mL of T7 RNA polymerase (expressed and purified in house). Reactions were incubated overnight (~16 hours) at 37°C. In order to remove template DNA, reactions were treated with 0.1 units/μL of Turbo DNase (Invitrogen) for 1 hour prior to purification. "GMP" transcription reaction conditions varied the nucleotide concentration as follows: 4mM each NTP, 20 mM GMP. All other components were not varied from the "GTP" transcription.

1.2. PAGE purification of oligonucleotides

Oligonucleotides derived from *in vitro* transcription or chemical synthesis was mixed in in FA9525 denaturing loading buffer (95% formamide, 25 mM EDTA, bromophenol blue) to a final concentration of >60% formamide. Samples were heated at 94°C for 5 minutes to denature the nucleic acid and separated on an 8 M Urea 1xTBE denaturing PAGE. Gels were run at constant 30W on an EV200 Large Format PAGE Unit Gel Unit (Cambridge Electrophoresis) using bromophenol blue as a marker for migration. UV shadowing was used to identify the band of interest. The gel fragment containing the band of interest was excised, crushed using a pipette tip, and suspended in TE buffer (10mM Tris·HCl pH 7.4, 1mM EDTA). The slurry was frozen in dry ice, thawed at 50 °C for 5 minutes and left rotating at 4 °C (2 hours to overnight). The eluate was filtered using a Spin-X 0.22 μm cellulose acetate filter (Costar) and precipitated in 73% ethanol (for ribozymes/long oligonucleotides) or 85% ethanol (oligonucleotides < 8 nt). Absorbance at 260nm of the purified nucleic acids was measured using a Nanodrop ND-1000 spectrophotometer (Thermo Fisher Scientific) and the concentration was determined based on the measured absorbance and the sequence using Oligocalc (*61*).

1.3. TGK RNA synthesis

TGK (a primer dependent DNA-dependent RNA polymerase protein using single-stranded DNA as template (*62*)) was used to synthesize marker oligonucleotides. RNA primers as in the reaction requiring a marker were used. DNA versions of the RNA templates used in the reaction by the ribozyme were used as templates. The primer extension reactions were carried out in 1x Thermopol buffer (NEB), 3 mM $MgSO_4$, 0.625 mM each NTP, 0.5 μM primer, 1 μM template, 150 nM TGK (94 °C 10 seconds, 40 °C 1 minute, 65 °C 1 hour, repeated once).

1.4. Adenylation

5′-end phosphorylated, 3′-end blocked DNA adapter was incubated at 20 μM for 2h at 65°C in 1x 5′ DNA Adenylation Reaction Buffer (NEB) supplemented with 1 mM ATP (Thermo Fisher Scientific) and 5 μM of Mth RNA ligase (NEB). Reaction size was usually 80 μL, but it was scaled

depending on need. DNA from the reaction was then PAGE purified alongside a non-adenylated DNA adapter that serves as control marker.

1.5. Ligation of RNA to pre-adenylated adapter

T4 RNA ligase 2 truncated KQ (NEB) was used to ligate the 3′-end of single-stranded RNA to a DNA adapter without a bridging oligonucleotide. Each reaction contained a maximum of 1µg/µL RNA-bound Dynabeads MyOne Streptavidin C1 (ThermoFisher Scientific) beads or 50 nM of RNA in solution. Ligation was carried out in 1x NEB RNA ligase buffer, 15% PEG8000, 2 µM adenylated adapter, 0.04% Tween 20 and 20 U/µL of ligase for 2 hours at room temperature.

1.6. Template preparation for *in vitro* transcription

The indicated DNA oligonucleotides were cross-extended in four cycles of thermal cycling in standard reaction conditions for GoTaq HotStart Green MasterMix (Promega). Reactions were purified using Qiaquick PCR purification kit (Qiagen) for products >100 base pairs, and with a Nucleotide Removal Kit (Qiagen) for products <100 base pairs in length.

1.7. Triplet and dimer transcription

Triplets and dimers were prepared via run-off *in vitro* transcription using T7 RNA polymerase. A detailed method can be found in (*13*). Briefly, reaction conditions were varied as follows: 100 pmoles of DNA template for each triplet was mixed with equimolar DNA '5T7'. The "GTP" transcription protocol with the exception that a lower total NTP concentration was used (4.32 mM) as this yielded better defined bands for purification. 50 µL transcription reactions were stopped by adding 2 µL EDTA and 5 µL of 100% glycerol. The reaction products were separated on a 30% acrylamide 3 M Urea denaturing PAGE gel using an EV400 DNA Sequencing Unit (Cambridge Electrophoresis). UV shadowing was used to identify bands, and the correct band, identified based on relative migration to known triplets was excised. The RNA was extracted from the gel fragment as in method 1.2. Correct sequence composition was confirmed by A260/280 absorbance ratio, measured with a Nanodrop ND-1000 spectrophotometer (Thermo Fisher Scientific)

1.8. Template-dependent ribozyme-catalyzed RNA synthesis: reaction setup-up and product detection

1.8.1. Reaction setup

Standard primer extensions reactions were typically conducted as follows: 5 pmoles of biotinylated fluorescently labelled primer was mixed in water with template, ribozyme (5TU+t1.5 or QT), triphosphorylated oligonucleotide substrate(s), and annealed (80°C 2 minutes, 17°C 10 minutes) in half the final reaction volume. The reaction components were then kept on ice until pre-chilled buffer and salts were added. Final buffer concentrations are described in each figure legend. Upon buffer addition the reactions were equalized on ice for ~1 minute, then frozen in dry ice for ~ 1 minute and transferred to a -7°C R4 series TC120 refrigerated cooling bath (Grant) for the time indicated in figure legends. Reactions were then stopped with equimolar EDTA to the Mg^{2+} in the reaction.

The concentrations indicated in the figure legends describe pre-freezing conditions, assuming that the final post-freezing operational volume is determined by the solute concentration in the sample. The formation of ice-crystals causes all solutes to concentrate to their final operating concentrations upon equilibration of the eutectic phase. pH is also expected to vary due to the temperature dependence of the buffer's pKa (*63*). For example, if 1 µmole of KCl and 1 pmole of RNA were added to a reaction volume of 10 µL or 20 µL, both are described as 100 mM KCl and

0.1 µM RNA of a virtual 10 µL pre-freezing reaction volume, as the concentrations in the eutectic phase would be identical post-freezing.

1.8.2. Product detection for analytical purposes

For biotinylated primers, reactions were incubated with Dynabeads MyOne Streptavidin C1 (Thermo Fisher Scientific) at 0.1 pmol biotinylated primer/µg of beads, in at least one reaction volume of BWBT (0.2 M NaCl, 10 mM Tris·HCl pH 7.4, 1 mM EDTA, 0.1% Tween-20). Beads were washed once in BWBT, twice in NaBET25 (25 mM NaOH, 1mM EDTA, 0.05% Tween 20), and again washed twice in BWBT before resuspending in FA9525 and heated at 94°C for 5 minutes in order to disrupt the biotin-streptavidin interaction and elute the primer from beads. The supernatant was run on an 8 M urea 1x TBE denaturing PAGE. Gels were analyzed on a Typhoon Trio scanner (GE Healthcare). For non-biotinylated primers, reactions were mixed in >60% FA9525 with 10-20-fold molar excess of unlabeled strand complementary to the template in the reaction (described as "competing oligos" in table S6) to prevent product/template reannealing. Upon denaturation (94°C 5 minutes) RNAs were separated on an 8 M Urea 1xTBE denaturing PAGE. Gels were analyzed on a Typhoon Trio scanner (GE Healthcare). Gel bands were quantified using the ImageQuant analysis software.

1.8.3. Product detection and recovery for selection and sequencing

Dynabeads MyOne Streptavidin C1 were added to the stopped reactions at 0.05 pmol biotinylated primer/µg of beads or an even greater excess of beads, in at least one reaction volume of BWBT. Then, the beads were washed twice with BWBT, twice with NaBET25 to confirm covalent linkage of construct to primer (and transferred to a fresh microcentrifuge tube to minimize downstream contamination between washes), and twice with BWBT, before resuspending in FA9525. Biotinylated RNA was eluted from the beads by disrupting the biotin-streptavidin interaction by heating at 94 °C for 5 minutes, and separated on an 8M Urea 1X TBE denaturing PAGE gel alongside RNA markers equivalent to successfully ligated constructs (generated by similar extension reactions but with added 5TU and t1.5 ribozymes). The marker-adjacent gel region in the construct lane was excised. Biotinylated RNA was then eluted and bound to MyOne Streptavidin C1 Dynabeads in BWBT overnight. After 50 µm filtering (Partec Celltrics (Wolflabs (York, UK))) of the supernatant to remove gel fragments, the beads were washed twice with BWBT, twice with NaBET25 (transferred to fresh tube between washes), and twice with BWBT before proceeding to subsequent reactions.

2. De novo selection

2.1. Dimeric construct selection protocol

Details specific to each selection round can be found in table S1. A diagram of the steps involved in the dimeric selection construct can be found in fig. S1. Round 1 constructs were prepared via the circularization of a starting material of 200 pmoles of ULTc2dN40/ULTc2dN30/ULTc2dN20 using equimolar 5T76FfGG as a splint. The two oligonucleotides were annealed (80 °C 2 minutes, 17°C 10 minutes) in 4/5 of the final reaction volume in 1X T4 DNA ligase reaction buffer (NEB). The components were kept on ice until prechilled T4 DNA ligase diluted in 1/5 of the reaction buffer was added at a final concentration of 8000 units/µL. Each oligonucleotide in the reaction was at a low concentration of 100 nM to favor intramolecular ligation. After 1 h incubation at 16 °C, dNTPs (GE Healthcare UK) were added to a final concentration of 800 µM and T4 DNA polymerase (NEB) was added at a final concentration of 0.012 units/µL. These were incubated at 25 °C for an additional hour. The product was purified using Qiaquick PCR Purification kit (QIAGEN).

From round 2 onwards, the libraries required an additional reaction step for the generation of a ssDNA equivalent to ULTc2dN40/ULTc2dN30/ULTc2dN20. To do so, 5 µL of RT-PCR reaction from the previous round was used as template for PCR with GoTaq HotStart Green MasterMix (Promega) for 14 cycles. 200 pmoles of primers forceGG17 and AACA2ULT were used. DNA Polymerase I, Large (Klenow) fragment (NEB) was added after PCR at a final concentration of 0.05 units/µL and incubated at room temperature for 15 minutes to generate blunt ends. Products were purified using a Nucleotide removal kit (QIAGEN). Lambda exonuclease (NEB) was used to remove the phosphorylated strand and generate ssDNA for subsequent steps, and repurified. The ssDNA was annealed with equimolar 5T76FfGG as a splint, and treated similarly to round 1, with an additional 5′-end phosphorylation step prior to ligation (1X T4 DNA ligase buffer, 0.25 units/µL T4 PNK (NEB), 30 minutes at 37°C, heat inactivated 20 minutes at 65 °C). The same reaction is diluted 5 fold in the same ligation/extension buffer described for round 1, except for a lower T4 DNA ligase concentration of 1600 units/µL. The product was Qiaquick PCR purified (QIAGEN).

After obtaining this purified product, 3 further reactions were carried out sequentially in one pot. The first reaction consisted in nicking of deoxy-uracil nucleotides in the oligonucleotide via USER enzyme treatment (0.05 units/µL USER enzyme (NEB) in 1X Cutsmart buffer supplemented with 5 mM DTT for 15 minutes at 37 °C), the second reaction was the dephosphorylation of 3′-ends (T4 PNK (NEB) at 0.5 units/µL for 45 minutes at 37 °C), lastly dNTPs and DNA Polymerase I, Large (Klenow) fragment (NEB) at a final concentration of 1 mM and 0.05 units/µL respectively were added to extend the 3′-ends, and incubated for an additional 45 minutes at 37 °C. Reactions were stopped using 6X Purple Gel Loading Dye (NEB) and products were separated on a 1X SYBR Safe (Life Technologies Ltd) 3.5% UltraPure Agarose (Life Technologies Ltd) gel. The correct products were excised, gel purified (QIAGEN) and used as templates for "GTP" transcription. The transcription was treated with Turbo DNase (Invitrogen) and PAGE purified as in method 1.2.

Reactions were set-up as in method 1.8.1 with equimolar primer/template to the RNA libraries (exact conditions for each round in table S1). The final concentrations before freezing were 50 mM $MgCl_2$, 200 mM KCl, 50 mM CHES-KOH pH 9, 0.05% Tween 20, with 50 nM primer/template/library in round 1 and round 12, and 20 nM in all other rounds. Reactions were stopped with equimolar EDTA to the Mg^{2+} present and products were recovered and bound to beads as described in section 1.8.3. The beads were then resuspended in the RT-PCR reaction containing pTLT reverse primer and varying forward primer covering part of the ligation junction from the ribozyme reaction (details of primers used in each round of the selection are in table S1). The RT-PCR product DNA was used in subsequent selection rounds or sequenced. Error prone PCR was carried out after round 5 using the GeneMorph II kit for mutagenesis (Agilent).

2.2. Monomeric selection

The selection in monomeric form followed similar steps to the one in dimeric form, with a simpler construct generation procedure. The ribozyme primer extension reaction set-up was identical, with recovery only diverging just before RT-PCR. The bead-bound biotinylated RNA was 3′-end dephosphorylated using T4 PNK and adapter ligated to a pre-adenylated adapter (HDVlig) prior to RT-PCR using primers HDVrec and forceGG (or a variable primer depending on the template used in the ribozyme primer extension, detailed in table S6). The RT-PCR product was then used as a template for a subsequent in-nest PCR, with primers 5T76FfGG and HDVrt used regenerate the T7 promoter and a HDV ribozyme 3′-end cassette. The DNA was subsequently purified using Qiaquick PCR purification kit (QIAGEN) and used for "GTP" transcription. The transcription was treated with Turbo DNase (Invitrogen) and PAGE purified as in method 1.2. The

selection steps were equivalent to the ones used in the dimeric selection of method 2.1. After selection, the recovered bead-bound RNA was dephosphorylated with T4 PNK for 1 hour, adapter ligated to AdeHDVlig as in method 1.5. The product was amplified via RT-PCR with the primers specified in table S6 using SuperScriptIII/Platinum Taq One Step RT-PCR system (Thermo Fisher Scientific). The resulting product was used in subsequent selection rounds or sequenced.

3. Regiospecificity assay

Single triplet (pppACC) incorporation reactions were set-up using a primer containing a single G at the 3′-end (FBAP9). Reactions were set up *in trans* for QT45 and 5TU+t1.5 using the following conditions: 0.25 μM ribozyme QT45 or 5TU+t1.5, 0.25 μM FBAP9 primer, 0.25 μM t6FAP9ACCCUG, 5 μM pppACC, 5 μM hoCUG, and 0.05% Tween 20, in 50 mM $MgCl_2$, 50 mM CHES-KOH, pH 9 for QT51, and in 200 mM $MgCl_2$, 50 mM Tris·HCl , pH 8.3, for 5TU+t1.5. Reactions were set up *in cis* for 1-30, 2-30, 1-40 using the following reaction conditions: 50 nM ribozyme 1-30 or 2-30 or 1-40, 50 nM FBAP9 primer, 50 nM t6FAP9ACCCUG, 5 μM pppACC, 5 μM hoCUG, and 0.05% Tween 20, in 50 mM $MgCl_2$, 200 mM KCl, 50 mM CHES-KOH, pH 9. Reactions were incubated for 63 days at -7 °C frozen. This reaction set-up ensured that only a single G is present just before the ligation junction, making it the target site for cleavage by RNase T1. A non-triphosphorylated triplet (hoCUG) is included downstream of the pppACC incorporation site to facilitate single triplet incorporation. The reaction was stopped, and the primer extension products were bound to streptavidin coated beads (Dynabeads MyOne Streptavidin C1) and separated from the template via denaturing washes (as described for selections). Single triplet incorporation products are PAGE purified and ethanol precipitated.

The RNase T1 cleavage reactions were set-up as following: 0.1 μM of RNA (either ribozyme generated or chemically synthesized standards) was incubated in 10 mM Tris·HCl pH 7.4, 2 mM EDTA, 250 units of RNase T1 (Thermo Scientific) for 30 minutes at 37°C. Chemically synthesized standards of 3'-5' or 2'-5' linkage (FBAP9_35ACC and FBAP9_25ACC) were used to confirm cleavage of only the 3'-5' bond. A chemically synthesized 3' phosphorylated oligonucleotide (FBAP9_3p) was used as marker of the correct cleavage product. The reactions were run on a 20% denaturing PAGE to separate the products.

4. Hammerhead ribozyme activity assay and sequencing

4.1. Seq0-HH hammerhead ribozyme synthesis, sequencing, and activity assay

The hammerhead synthesis reactions were described in the figure legend (Fig. 3). To facilitate product amplification for sequencing, reactions were set-up in parallel under identical conditions using a primer containing a 5′ DNA overhang (bioCy3KyleP10). Synthesized products were recovered as in method 1.8.3 and used for the subsequent sequencing step. The recovered biotinylated products were adapter ligated to AdeHDVlig as in method 1.5. After adapter ligation, the RNA was amplified via RT-PCR with primers kyle_F and HDVrec using SuperScriptIII/Platinum Taq One Step RT-PCR system (Thermo Fisher Scientific). The resulting product was used in subsequent sequencing reactions (method 7) or used to generate hammerhead RNA and assay its activity. To do so, the RT-PCR product was PCR amplified using 5T7kyleF and HDVrt primers. A wildtype control for seq0-HH was prepared by PCR of a DNA oligo (seq0-hh-DNA). The PCR product was purified and used for T7 *in vitro* transcription and PAGE purified.

The hammerhead activity time-course assay was set up with the following reaction conditions: 0.25 μM substrate (Fsub-seq0HH), 0.2 μM seq0-HH (QT45 synthesis derived or control), 0.05% Tween 20, 20 mM $MgCl_2$, 50 mM Tris·HCl pH 8 in 10 μL at 23 °C. Each reaction was set-up individually and stopped at the timepoints shown in Figure 3 by quenching by addition of 30 μL of 95% formamide 25 mM EDTA, immediately frozen in dry ice and stored at -70 °C.

Each timepoint was carried out in triplicates. The fraction of cleaved product was quantified using ImageQuant software and plotted in Figure 3D (bottom) using matplotlib. A single exponential curve was fit to the data. Curve fitting was performed using scipy.optimize.curve_fit with weighted least squares, where weights were derived from the standard deviation of three technical replicates.

Hammerhead ribozyme cleavage follows first order kinetics described by $P_t = P_{inf} \times (1 - e^{(-kobs \times t)})$, where P_t is the fraction cleaved at time t, P_{inf} is the maximum fraction cleaved at the reaction endpoint, and kobs is the observed rate constant. Hence, fitting the curve involves determining the values of P_{inf} and kobs that best fit the data. These determined values were then used to calculate the initial rate, which is given by the equation $dP/dt|(t=0) = P_{inf} \times kobs$. Finally, the calculated QT45-synthesis-derived initial rate was divided by the calculated wild-type hammerhead initial rate to assess the activity of the QT45-synthesised hammerhead ribozyme compared to the perfect wildtype hammerhead ribozyme control.

4.2. Hammerhead fragment synthesis, sequencing, and direct assaying

The hammerhead fragment synthesis reaction conditions were described in the figure legend (fig. S25). Three different primers were used depending on the subsequent steps. For visualization, the reaction was set-up using BCy3P10, to assay the activity of the synthesized product, A647BP10 was used in order to simultaneously scan the synthesized hammerhead and its FAM labelled substrate. For sequencing, a primer containing a 5′ DNA overhang was used (bioCy3KyleP10) to facilitate product amplification. Synthesized products were recovered as in method 1.8.3 and used for the subsequent sequencing step. The recovered biotinylated products were adapter ligated to AdeHDVlig as in method 1.5. After adapter ligation, the RNA was amplified via RT-PCR with primers kyle_F and HDVrec using SuperScriptIII/Platinum Taq One Step RT-PCR system (Thermo Fisher Scientific). The resulting product was used in subsequent sequencing reactions (method 7).

To assay hammerhead ribozyme activity, a two-fold molar excess of FAM labelled substrate (Fsubuhl) was mixed with A647 labelled hammerhead (either synthesized by QT51, 5TU or TGK). Reactions contained 10 mM Tris·HCl pH 8, 2 mM $MgCl_2$, and 0.05% Tween-20. Prior to $MgCl_2$ addition the reaction was incubated at 60 °C for 1 minute, then moved to ice. To start the reaction $MgCl_2$ was added and the sample was frozen in dry ice and incubated at -7 °C for 24 hours to let the reaction occur in the eutectic concentrate. The reaction was quenched in 65% FA9525 and analyzed via denaturing PAGE as in method 1.8.2.

5. Ribozyme-catalyzed synthesis of itself and its complementary strand

For (+) strand self-synthesis reactions all reaction components (described in detail in the corresponding figure legend) were mixed in a total volume of 125 µL at room temperature. To reduce recombination, the ribozyme used contained a 5′-phosphate (pQT45). To reduce strand-reannealing, a single cycle of heating and acidification followed by quick freezing was used to initiate the reactions (as in (*32*)), described in method 6. Reactions were then incubated at -7 °C for the time indicated in the figure legend.

For (-) strand synthesis, the reaction was set up as described in detail in the corresponding figure legend in a volume of 125 µL at room temperature, then frozen at -7 °C to start the reaction. The reactions used for sequencing were set-up as in method 1.8.1, with the following conditions: 0.25 µM primer BCy3P10, 0.25 µM template t4msP10QT45, 10 µM QT45 or 0.5 µM each 5TU and t1.5, 2.5 µM each of the 15 defined triplets or 1.75 µM each of the 64 triplets, 0.05% Tween 20, 50 mM MgCl2, 50 mM CHES-KOH, pH 9, 28 days at -7 °C frozen.

To sequence the reactions, synthesized products were recovered as in method 1.8.3 and used for the subsequent sequencing step. The recovered biotinylated products were adapter ligated to

AdeHDVlig as in method 1.5. After adapter ligation, the RNA was amplified via RT-PCR with primers ggP10 and HDVrec using SuperScriptIII/Platinum Taq One Step RT-PCR system (Thermo Fisher Scientific). The resulting product was used in subsequent sequencing reactions (method 7).

6. pH-freeze-thaw cycle for dsRNA copying

For reactions where the complementary strand to the template was present, the reaction protocol from (*32*) was used to facilitate strand separation and reduces the extent of strand reannealing. Briefly, this consists in a 125 µL replication buffer in 0.5-ml Eppendorf DNA low-bind tubes where the template strand and buffer are in dilute conditions ≤ 8 nM template, 1 mM CHES-KOH pH 9, 0.01% Tween 20, 0.3-1.2 mM KCl, 0.4 mM $MgCl_2$ buffer. Reaction components were mixed at room temperature, and the pH cycling was begun by adding 0.75 µL of 0.1 M HCl (to overwhelm the CHES buffer and lower the pH). The reaction was then vortexed and heated to 80 °C for 2 minutes to denature the RNA strands. After the incubation, 0.75 µL of 0.1 M KOH was added to neutralize the reaction, which was briefly vortexed and flash frozen in liquid N_2 for 20 seconds. The frozen reactions were then incubated at -7 °C for the time described in each figure legend.

7. High-throughput sequencing

7.1. Sequencing libraries preparation

Libraries for Illumina sequencing were prepared from previously amplified RT-PCR product, by further PCR amplification with primers containing 5′ overhangs that introduce features needed for sequencing. The PCR was carried out using GoTaq HotStart Green MasterMix (Promega). An example of the PCR primers used to prepare libraries is shown below. Italicized is the region that hybridizes to the flow-cell. Underlined is the section where sequencing primers hybridize on the sequencer. A random three nucleotide “NNN” section is introduced to ensure high diversity in the beginning of the read. A four-nucleotide barcode sequence, denoted as “XXXX” is used to provide a unique barcode to each of the libraries sequenced in the same run, and it is de-multiplexed computationally after sequencing. “(land)” refers to the library-specific landing primer used.

>P5NNNXL
*AATGATACGGCGACCACCGAGATCTACAC*TCTTTCCCTACACGACGCTCTTCCGATCT**NNN**XXXX(land)

>P7NNNXL
*CAAGCAGAAGACGGCATACGAGAT*GTGACTGGAGTTCAGACGTGTGCTCTTCCGATCT**NNN**XXXX(land)

The amplified libraries containing the correct overhangs were agarose gel purified (Qiagen) and pooled together. Pooled libraries were quantified using a Qubit 2.0 Fluorometer (Thermo Fisher Scientific) using the high sensitivity dsDNA Quantification Assay Kits (Invitrogen), prior to denaturation and dilution in HT1 buffer (Illumina) to be sequenced on a MiSeq System, HiSeq 2500, or NextSeq 2000 (Illumina).

7.2. Analysis of sequencing data

We computationally pre-processed the sequencing data in order to facilitate downstream analyses. For the mini hammerhead and (+)/(-) sequencing data, paired-end reads were merged using PEAR (*64*). We then trimmed the single-end reads or merged paired-end reads using the

BBtools pipeline (*65*) and quality filtered the reads for a minimum base quality of 30 over 100% of the read using the FASTX-Toolkit (*66*). The individual libraries were demultiplexed using Cutadapt (*67*). For the full hammerhead sequencing data, paired-end reads were merged and filter to have less than 5% of the read with quality<Q30 using fastp (*68*). Individual libraries were demultiplexed using Cutadapt.

For the fidelity analysis of ribozyme products, the pre-processed reads were aligned to the correct product using BBMap and Samtools. This generated a pileup file that was used for subsequent analysis and visualization using Python. The output pileup files were parsed in Python using the Pandas dependency and plotted as heatmaps using Matplotlib and Seaborn. Alignments of a small number of clones were generated using MAFFT (*69*) on the Aliview visualization software (*70*).

8. <u>Synthesis of adenylated triplets</u>

The 5'-adenylated trinucleotide AppGCA was synthesized by a method based on that of (*71*). Briefly, the 5'-monophosphates of adenosine (5'-AMP) and guanosine (5'-GMP) were coupled via phosphorimidazolide chemistry to make the 5'-5' pyrophosphate-containing AppG intermediate, which was then fed into a triplet transcription reaction.

First, the 5'-phosphorimidazolide of adenosine was synthesized according to (*72*). 0.6 mmol (1 equivalent, or eq.) of adenosine 5'-monophosphate was mixed with 5 eq. imidazole in water and titrated to pH 5 with HCl. The mixture was lyophilized and then dissolved in 30 mL dimethyl sulfoxide. 9 eq. of triphenylphosphine and 10 equivalents of 2,2′-dipyridyl disulfide were added to the solution and stirred under ambient conditions for 1 hour. The product was precipitated by addition to a solution of 400 mL acetone, 250 mL diethyl ether, 30 mL triethylamine, and 1.6 mL of sodium perchlorate-saturated acetone. The supernatant was decanted and the remaining precipitate pelleted by brief centrifugation. The pellet was first washed with a solution containing 2.2 mL TEA, 18 mL ether, and 29 mL acetone and then washed twice with 50-mL volumes of a 1:1 solution of acetone and ether. The pellet was dried under a stream of nitrogen gas. No further purification was conducted.

Next, AppG was obtained by reacting the 5'-phosphorimidazolide of adenosine (1 eq., 10 µmol) with 5'-GMP (1 eq.) in the presence of $MgCl_2$ (2 eq.) in 0.2 mL 50% (by volume) aqueous formamide at 50 °C for 4 hours. The reaction was mixed with 4 eq. of disodium EDTA (pH 8) and diluted to 2 mL in 0.1 M triethylammonium bicarbonate, pH 8.5. The product was purified by IP-HPLC using an Atlantis T3 Prep OBD column (5-micron particle size, 19 mm x 250 mm) and a 30-minute linear gradient from 0.5% acetonitrile to 15% acetonitrile in aqueous 0.1 M triethylammonium bicarbonate, pH 8.5. The product was lyophilized and resuspended in water. The concentration was determined by UV absorbance based on the sum of the individual 5'-AMP and GMP extinction coefficients.

Finally, AppG was extended by T7 RNA polymerase to AppGCA in a standard triplet transcription protocol in which the only other nucleotides added were CTP and ATP. The triplet was purified by HPLC using an Xbridge BEH Shield RP18 OBD Prep column (5-micron particle size, 10 mm x 250 mm) and a 20-minute linear gradient from 0.5% acetonitrile to 5% acetonitrile in aqueous 0.1 M triethylammonium bicarbonate, pH 8.5. After lyophilization, the product was suspended in 0.2 mL 200 mM NaCl and precipitated with 5.67 volumes of ethanol and centrifugation for 30 minutes at 21000 rcf. The pellet was washed once with 85% ethanol and air dried prior to resuspension in water.

9. Fitness landscape generation

The fitness landscape of QT45 was determined twice, the first time with varied libraries and templates (3 UGC, 12 CUA, 3 AUA) and the second time with a single optimized library and a 3 UGC template. The fitness landscape derived from the first approach with varied libraries and templates is included in the SM and described first. The fitness landscape derived from the second approach with a single optimized library and a 3 UGC library is included in the main text and described after.

9.1. Fitness landscape of QT45 (varied libraries and templates)

9.1.1. Selection library templates

For construction of the QT45 library for fitness landscape determination, equimolar amounts of mutagenized oligonucleotides encoding variants of QT45 were mixed together. The starting sequences chosen for mutagenesis included either known neutral mutations, or known neutral indels (from a previous small scale fitness landscape), informing the oligos used for the fitness landscape (QT45MO10, QT45MO10 iG1 dC45, QT45MO10 dG1 iC45, QT45MO10 C21D ins1G, QT45MO10 C21D ins45C, QT45MO10 C21D ins20G, QT45MO10 C21D ins22G, QT45MO10 U23D ins45C, and QT45MO10 U23D ins1G). These various starting sequences would enable the exploration of the mutational landscape starting from slightly different positions in sequence space. To obtain oligonucleotides encoding the HDV ribozyme at the 3'-end, 2 nmol of the resultant mixture was then ligated to 2 nmol of HDVrest, using T4 DNA ligase and 2 nmol of HDVspl as a complementary DNA splint. After heat inactivation and spin concentration with a 3 kDa molecular weight cut-off filter, the construct was urea-PAGE purified. The product band was excised, eluted in 10 mM Tris·HCl pH 7.4 overnight, and precipitated in 73% ethanol.

To generate the template for transcription, 260 pmol of the resultant oligonucleotide was then mixed with 260 pmol of partially complementary oligonucleotide 5T76F6LfGG and Bst2.0 polymerase (NEB) was used to 'fill-in' the single-stranded regions to make a double-stranded product with a short linker (sp12-6L library template). Similarly, 20 pmol of resultant oligonucleotide from the above ligation step was also 'filled-in' with 5T76F8LfGG using Bst2.0 to make a similar product but with a longer linker (sp12-8L library template). Both products were then Qiaquick PCR purified (Qiagen) and transcribed overnight using T7 RNA polymerase to yield RNA selection libraries. Products were subsequently purified using preparative-scale urea-PAGE. The product band was excised, eluted in 10 mM Tris·HCl pH 7.4 overnight, and precipitated in 73% ethanol.

9.2. In vitro evolution cycle, recovery, and sequencing

10 pmol of sp12-6L library was annealed with equimolar temp6FP10UGC3 template, BCy3P10 primer, and 1 nmol of UGC triplet in 250 µl of water with 0.1% tween (80°C 2 min, 17°C 10 min). The annealed mixture was then placed on ice, 250 µl of chilled 2x extension buffer (100 mM CHES, 100 mM $MgCl_2$) was added, and the reaction was then frozen with dry ice and incubated at -7 °C for 7 hours.

After the incubation, the reaction was stopped with equimolar EDTA, and the products were recovered and bound to beads as described in section 1.8.3. Then, the 3' end of the bead-bound constructs were first dephosphorylated with T4 PNK for 1 hour (with T4 PNK added after bead resuspension in other reaction components including 0.05% Tween-20) and AdeHDVlig was subsequently ligated to the dephosphorylated 3'-end for 2 hours (with RNA ligase 2 truncated KQ added after bead resuspension in other reaction components including 0.4% Tween-20). Beads were washed twice in BWBT and used in a 50 µl RT-PCR. Reverse transcription and PCR were carried out using HDVrec and P10UGCugFrec primers using the SuperScriptIII/Platinum Taq One

Step RT-PCR system (Thermo Fisher Scientific). 4 µl of the resulting product was run on a 4% agarose gel to check the size of the products. The remaining product was Qiaquick PCR purified and then further amplified using primers that introduce indexed adapters for Illumina sequencing (P71forceGG_2024 and P51HDVba_2021) to generate the sequencing construct in a 50 µl GoTaq HotStart (Promega) PCR. Products were purified by 4% agarose gel, quantified on a Qubit 2.0 fluorometer, and then sequenced on a NextSeq 2000 (Illumina).

The above process was repeated to screen for activity on two other templates encoding 3 AUA and 12 CUA with similar setups. Regarding the 3 AUA extension setup, 10 pmol of sp12-6L library was annealed with equimolar temp6FnewnewP12AUA3 template, BCy3newnewP12 primer, and 2.5 nmol of AUA triplet in 250 µl of water with 0.1% tween (80 °C 2 min, 17 °C 10 min). The annealed mixture was then placed on ice, 250 µl of chilled 2x extension buffer (100 mM CHES, 100 mM MgCl2) was added, and the reaction was then frozen with dry ice and incubated at -7 °C for 17 hours. Regarding the 12 CUA extension setup, 10 pmol of sp12-8L library was annealed with equimolar temp6FP10CUA12 template, BCy3P10 primer, and 2.5 nmol of CUA triplet in 250 µl of water with 0.1% tween (80 °C 2 min, 17 °C 10 min). The annealed mixture was then placed on ice, 250 µl of chilled 2x extension buffer (100 mM CHES, 100 mM MgCl2) was added, and the reaction was then frozen with dry ice and incubated at -7 °C for 2 days. Downstream recovery processes were similar except that reverse transcription and PCR were carried out using HDVrec and newnewP12auaaFrec primers for the AUA sample, and HDVrec and forceGG primers for the CUA sample. Additionally, primers used to generate the sequencing constructs were also different. For the 3 AUA post-selection sample, primers P71forceGG_2024 and P53HDVba_2021 were used. For the 12 CUA post-selection sample, primers P71forceGG_2024 and P52HDVba_2021 were used. Pre-selection libraries were also amplified using primers that introduce indexed adapters for Illumina sequencing. The sp12-6L library was similarly PNK treated, adapter ligated with adeHDVlig, RT-PCR with forceGG and HDVrec, and finally PCR with P72forceGG_2024 and P512HDVba_2021. The sp12-8L library was processed similarly, except that P72forceGG_2024 and P514HDVba_2021 were used in the final PCR step.

9.3. Calculating fitness associated with each genotype

Reads from the NextSeq run were merged using PEAR (*64*) (where applicable) and reads from separate sequencing runs were combined. Reads were then demultiplexed into their respective libraries (input and 3 output libraries - 3 UGC, 3 AUA, 12 CUA) using Cutadapt, according to 6-nucleotide barcodes. Adapters were trimmed away also using Cutadapt, leaving only the variable QT45 sequence. Using FASTX-toolkit, reads were quality filtered such that each read contains only bases with Q-score 20 or above, and then identical sequences were collapsed while maintaining read count. Genotypes containing 10 reads or more in the input libraries, as well as at least 1 read in each of the 3 output libraries (3 UGC, 3 AUA, 12 CUA), were retained for downstream analysis to generate the single mutant fitness values. Genotypes containing 10 reads or more in the sp12-6L input library and at least 1 read in the 3 UGC output library were used to generate the double mutant fitness values. As the sub-library QT45MO10 dG1 iC45 was the most active and hence the most abundant in the output libraries, the fitness landscapes were generated using genotypes from this sub-library.

To determine the fitness associated with each genotype, the fraction of each library occupied by each genotype in the input and 3 output libraries were first calculated. The enrichment of each genotype during selection was then obtained by dividing the fractional abundance in the output library by that in the input library. Then, the fitness of each genotype was calculated as the log2 ratio of the enrichment of the genotype during selection and the enrichment of the wild-type

sequence during selection (*31*). Consequently, wild-type QT45 has a fitness of 0, while less active mutants have fitness values less than 0 and more active mutants have fitness values more than 0. An average of the three fitness values determined using the 3 output libraries was plotted to generate the single mutant fitness landscape, whereas the fitness value determined using the 3 UGC output library was used to plot the double mutant fitness landscape.

9.4. QT39 fitness landscape

Determination of the QT39 fitness landscape followed a similar approach described above for QT45. However, instead of 3 output libraries of different templates encoding different triplets (3 UGC, 3 AUA, 12 CUA) used to generate the QT45 fitness landscape, three technical replicates of the same template (3 CUA) were used to generate the QT39 fitness landscape. Downstream processing also followed a similar procedure, with minor differences. Following PEAR merging of reads, reads were trimmed and demultiplexed with FASTX-toolkit, further trimmed with Cutadapt, then quality filtered to Q-score 30 or above and collapsed with FASTX-toolkit.

9.5. Fitness landscape of QT45 (optimized library and three technical replicates)

The second determination of the fitness landscape involved a single optimized library and a 3 UGC template and is described below.

9.5.1. Selection library synthesis

For construction of the QT45 library for determination of the fitness landscape, equimolar amounts of oligonucleotides (listed in Table S7 for 'nodel' fitness landscape library generation) were mixed together. To obtain oligonucleotides encoding the HDV ribozyme at the 3' end, 2 nmol of the resultant mixture was then ligated to 2 nmol of HDVrest, using T4 DNA ligase and 2 nmol of HDVspl as a complementary DNA splint. After heat inactivation and spin concentration with a 3 kDa molecular weight cut-off filter, the construct was urea-PAGE purified on a 15% acrylamide gel. The product band was excised, eluted in 10 mM Tris·HCl pH 7.4 overnight, and precipitated in 73% ethanol. To generate the template for transcription, 160 pmol of the resultant oligonucleotide was then mixed with 160 pmol of partially complementary oligonucleotide 5T76F6LfG and Bst2.0 polymerase (NEB) was used to 'fill-in' the single-stranded regions to make a double-stranded product. This product was then Qiaquick PCR purified (Qiagen) and transcribed overnight using T7 RNA polymerase to yield RNA selection libraries. DNA templates were removed using 0.1 units/µL of Turbo DNase (Invitrogen) for 1 hour prior to purification. RNA libraries were subsequently purified using preparative-scale urea-PAGE with 15% acrylamide. The product band was excised, eluted in 10 mM Tris·HCl pH 7.4 overnight, and precipitated in 73% ethanol.

A similar process was used to generate the single deletion fitness landscape library, starting from oligonucleotides listed in Table S7 for 'del' fitness landscape library generation.

9.5.2. In vitro evolution cycle, recovery, and sequencing

Three technical replicates were setup and processed separately from the in vitro evolution cycle onwards. 10 pmol of 'nodel' library was annealed with equimolar temp6FP10UGC3 template, BCy3P10 primer, and 1 nmol of UGC triplet in 250 µl of water with 0.1% tween (80 °C 2 min, 17 °C 10 min). The annealed mixture was then placed on ice, 250 µl of chilled 2x extension buffer (100 mM CHES, 100 mM $MgCl_2$) was added, and the reaction was then frozen with dry ice and incubated at -7 °C for 16 hours. A similar primer extension was set up for the 'del' library, except that 10 pmol of a mixed library comprising 0.5 pmol of 'nodel' library and 9.5 pmol of

‘del’ library was used. The ‘nodel’ library was added to allow for normalization to wild-type QT45 during analysis.

After incubation, reactions were stopped with equimolar EDTA and the products were recovered and bound to beads as described in section 1.8.3. Then, the 3’ end of the bead-bound constructs were first dephosphorylated with T4 PNK for 1 hour (with T4 PNK added after bead resuspension in other reaction components including 0.05% Tween-20) and AdeHDVlig was subsequently ligated to the dephosphorylated 3’ end for 2 hours (with RNA ligase 2 truncated KQ added after bead resuspension in other reaction components including 0.4% Tween-20). The beads were washed twice in BWBT and used in a 50 µl RT-PCR. Reverse transcription and PCR were carried out using HDVrec and P10UGCugFrec primers using the SuperScriptIII/Platinum Taq One Step RT-PCR system (Thermo Fisher Scientific). 4 µl of the resulting product was run on a 4% agarose gel to check the size of the products. The remaining product was Qiaquick PCR purified and then further amplified using primers that introduce indexed adapters for Illumina sequencing (P71forceG_2024 and P5xHDVba_2021 as listed in Table S7) to generate the sequencing construct in a 50 µl GoTaq HotStart (Promega) PCR. Products were purified by 4% agarose gel, quantified on a Qubit 2.0 fluorometer, and then sequenced on a NextSeq 2000 (Illumina).

Pre-selection libraries were also amplified using primers that introduce indexed adapters for Illumina sequencing. The libraries were similarly treated with T4 PNK, adapter ligated with adeHDVlig, used as templates during RT-PCR with primers forceG and HDVrec, and finally amplified during PCR with P71forceG_2024 and P5xHDVba_2021 (as listed in Table S7) to generate the sequencing construct.

9.5.3. Calculating fitness and epistasis associated with each genotype

Reads from the NextSeq run were merged using PEAR (*64*). Then, they were demultiplexed into their respective libraries (input libraries ‘in nodel’ and ‘in del’ and output libraries ‘out nodel A’, ‘out nodel B’, ‘out nodel C’, ‘out del A’, ‘out del B’, ‘out del C’) using Cutadapt, according to 6-nucleotide barcodes. Adapters were trimmed away using the same software, leaving only the variable QT45 sequence. Using FASTX-toolkit, reads were quality filtered so that each read contains only bases with Q-score 20 or above, and then identical sequences were collapsed while maintaining read count.

To calculate single mutant fitness values used for plotting the colours on the secondary structure and to calculate double mutant fitness values used for plotting the fitness landscape, reads from ‘out nodel A’, ‘out nodel B’, ‘out nodel C’ were combined and reads from ‘out del A’, ‘out del B’, ‘out del C’ were combined. Genotypes containing 10 reads or more in the input libraries, as well as at least 1 read in the corresponding combined output libraries were retained for downstream analysis to generate fitness values.

To determine the fitness associated with each genotype, the fraction of each library occupied by each genotype in the input and output libraries were first calculated. The enrichment of each genotype during selection was then obtained by dividing the fractional abundance in the output library by that in the input library. Then, the fitness of each genotype was calculated as the log2 ratio of the enrichment of the genotype during selection, and the enrichment of the wild-type sequence during selection (*31*). Consequently, wild-type QT45 has a fitness of 0, while less active mutants have fitness values less than 0 and more active mutants have fitness values greater than 0.

To calculate epistasis values used to plot Figures S21 and S22, the two constituent single mutant fitness values (calculated as described above) were subtracted from each double mutant fitness value (calculated as described above). To calculate fitness and epistasis values shown in Table S3, S4, and S5 and used to determine base pairing, reads from ‘out nodel A’, ‘out nodel B’,

‘out nodel C’ were kept separate. Genotypes containing 10 reads or more in the input libraries, as well as at least 1 read in each of the three corresponding combined output libraries were retained for downstream analysis to generate fitness values and subsequently used to calculate epistasis values. In these calculations, an approach described in (*31*) that considers sampling error associated with a given read count (*73*) was used. In this approach, the fractional abundances of each genotype (in input and in output libraries) are calculated for each technical replicate separately as described above. Then, values from the replicates are merged as an average, weighted by the inverse of the variance of the genotype. Using these fractional abundance values, fitness values for each genotype are calculated also as described above. Errors for these fitness values are also calculated. These fitness values and their errors were then used to calculate epistasis values and their propagated errors. A one-sample t-test using these epistasis values, and their propagated errors were used to check whether these fitness values were significant. The Benjamini-Hochberg method was subsequently used to adjust the false discovery rate (*74*).

Supplementary Text

Fitness and epistasis values data processing

To enhance the reliability of derived fitness values and reduce noise from the selection and sequence recovery processes, three technical replicates were conducted for both the single deletion library and the full-length library. The calculated single and double mutant fitness values from these three replicates demonstrated strong correlation (fig. S14).

Given the strong inter-replicate correlation, reads from all three replicates were merged to generate the comprehensive fitness landscape and secondary structures colored by single mutant and single deletion fitness data (fig. 2D, 2E). This merging approach was used to visualize overall trends across the sequence space and provided enhanced coverage, yielding fitness values for all possible single mutants, all possible single deletions, and 98.0% of all double mutants in the library. The resulting fitness values were subsequently used to calculate epistatic interactions for general epistatic landscape plotting. The epistatic landscape shown in Figure S22 was generated using epistasis values calculated from these fitness values to provide comprehensive coverage of epistatic interactions across the sequence space.

For the analysis of epistatic interactions used to determine base pairing positions, reads from the three replicates were processed separately to account for sampling error associated with read count variation (*73*). Fractional abundance values were calculated independently for each replicate, then merged as a weighted average using the inverse variance of each value. Fitness values for each genotype were derived from these averaged abundance values. Epistasis was calculated by subtracting component single mutant fitness values from the corresponding double mutant fitness value. These epistasis values were then used for subsequent analysis of sequence constraints and base pairing interactions.

Statistical significance was assessed by calculating errors for fitness values and performing one-sample t-tests using fitness values and their propagated errors. A one-sample t-test was appropriate as it was used to compare the mean fitness of each mutant (derived from technical replicates) to a known reference (wild-type fitness of 0). Propagated errors were incorporated to account for measurement uncertainty. The Benjamini-Hochberg method was applied to control the false discovery rate (*74*). Only statistically significant epistasis values were used for analysis of reciprocal sign epistasis (Table S3) and large positive epistasis (Table S4).

To identify base-paired nucleotides, we developed an algorithm that analyzes mutation effects on epistasis and fitness. The algorithm first identifies all position pairs capable of forming Watson-Crick or wobble base pairs, then classifies double mutants involving candidate positions as either base pair-breaking or base pair-retaining. Pairs are retained only if they contain at least three base pair-retaining double mutants with average positive epistasis, and the average epistasis and fitness for base pair-retaining double mutants must exceed those of base pair-breaking double mutants by at least 1.0. Pairs are ranked by average epistasis of base pair-retaining double mutants, with non-overlapping constraints applied by selecting the highest-ranked pair for each position. While ribozymes exhibit dynamic structures with multiple conformations, this approach provides insight into a probable secondary structure configuration. The algorithm identified the following base pairs based on fitness and epistasis analysis: G10-C36, U16-A34, G18-C30, C8-G39, G7-C40, and G6-C41. Fitness and epistasis values of these base pairs are provided in Table S5.

Figure S21 presents an overview of the evidence supporting the identified base pairs, using the same fitness values used to plot the fitness landscape and secondary structure in Figure 2D and Figure 2E respectively. Base pair-retaining mutations generally exhibit higher fitness compared to base pair-breaking mutations (fig. S21A), though it should be noted that these base pairs contribute to both structural integrity and catalytic function. Therefore, sequence constraints for maintaining activity extend beyond simple base pair retention, and not all base pair-retaining mutations will preserve high activity levels. Similarly, epistasis values for base pair-retaining mutations are typically higher than those for base pair-breaking mutations (fig. S21B).

Analysis of reciprocal sign epistasis identified the strongest evidence for the most robust base pairs (Table S3). Reciprocal sign epistasis represents one of the strongest forms of epistatic interaction, where the fitness effect of mutation A changes sign when occurring in the background of mutation B, and vice versa (*75*). This analysis revealed strong evidence for canonical base pairs U16-A34, G7-C40, and C8-G39, as well as the non-canonical base pair C11-U35.

Analysis of double mutants exhibiting significant and large positive epistasis (Table S4) provided further evidence for the strong canonical base pairs: U16-A34, G10-C36, G18-C30, and C17-G31.

<u>Estimated of abundance of the QT functional motif</u>

The fraction of functional QT motifs in 45 nucleotide random sequences was calculated based on the fitness landscape data reported in Figure 2, and following a similar approach to (*20*). The fitness landscape data informed us on the ribozyme positions that contain critical base pairs and the tolerance to single substitutions of the remaining positions. A fitness threshold of 80% of wildtype activity was used to define a variant as active. Using this threshold, we could determine the fraction of possible base pairs that can be tolerated at critical base pairs sites (6-41: 13/16, 7-40: 5/16, 8-39: 6/16, 10-36: 1/16, 11-35: 2/16, 16-34: 2/16, 18-30: 2/16), and the fraction of possible nucleotides that can be tolerated at each non-base pairing position (2: 4/4 , 3: 4/4 , 4: 4/4 , 5: 3/4 , 9: 1/4 , 12: 1/4, 13: 4/4, 14: 1/4, 15: 2/4, 17: 1/4, 19: 1/4, 20: 1/4, 21: 4/4, 22: 1/4, 23: 1/4, 24: 4/4, 25: 1/4, 26: 1/4, 27: 1/4, 28: 1/4, 29: 1/4, 31: 1/4, 32: 1/4, 33: 1/4, 37: 1/4, 38: 2/4, 42: 3/4, 43: 4/4, 44: 4/4, 45: 4/4). The overall probability of emergence was calculated as the product of probabilities across all positions and base pairs, assuming independence, yielding 9.5×10^{-17}.

<u>Error threshold computational numerical model</u>

We implemented a deterministic replicator-mutator model to study sequence evolution under experimentally measured fitness and mutation landscapes. The model represents the genotype space as the master sequence (Hamming distance, HD = 0), all single mutants (HD = 1), all double mutants (HD = 2), and a lumped class containing all genotypes with HD ≥ 3. Population dynamics were described by the following system of Ordinary Differential Equations representing a typical Quasispecies model:

$$dx/dt = QAx - \phi x$$

With x the vector containing the concentrations of all the genotypes. The replication matrix A was constructed as a diagonal matrix containing the replication rate of each genotype. The experimental measurements are proxies for replication rates based on the synthesis of a defined short RNA sequence, and not direct measures of self-replication fitness. We use these fitness values in the replication matrix A despite the mismatch, as they are the only quantitative data we have for this

large number of genotypes. Rare values for fitness for HD=2 mutants that are missing from the dataset, as well as HD=3 lumped class are given an arbitrary growth rate value of 0.01. The mutational matrix Q was constructed by assuming a uniform per-nucleotide fidelity and that each substitution was equiprobable, with rates reflecting mutations from replication of both strands. Q was normalized such that all residual probability in each column were assigned to the HD $\geq$ 3 class, which was modeled as absorbing (Q is column-stochastic). The model was numerically integrated from an initial condition consisting entirely of the master sequence. The outflux term ϕ implements selection through a standard chemostat term that keeps the total concentration constant. The system of differential equations is numerically integrated using scipy solve_ivp with default parameters, for 500 time-steps which in all cases is sufficient to reach steady state. The fraction of the population with fitness $\geq$ 0.8 at each of the fidelity values between 90% and 99.5% is plotted for every increase of 0.5% fidelity, with this class being dominated by the genotype with highest fitness. The code is implemented in Python and is available on Zenodo (*59*).

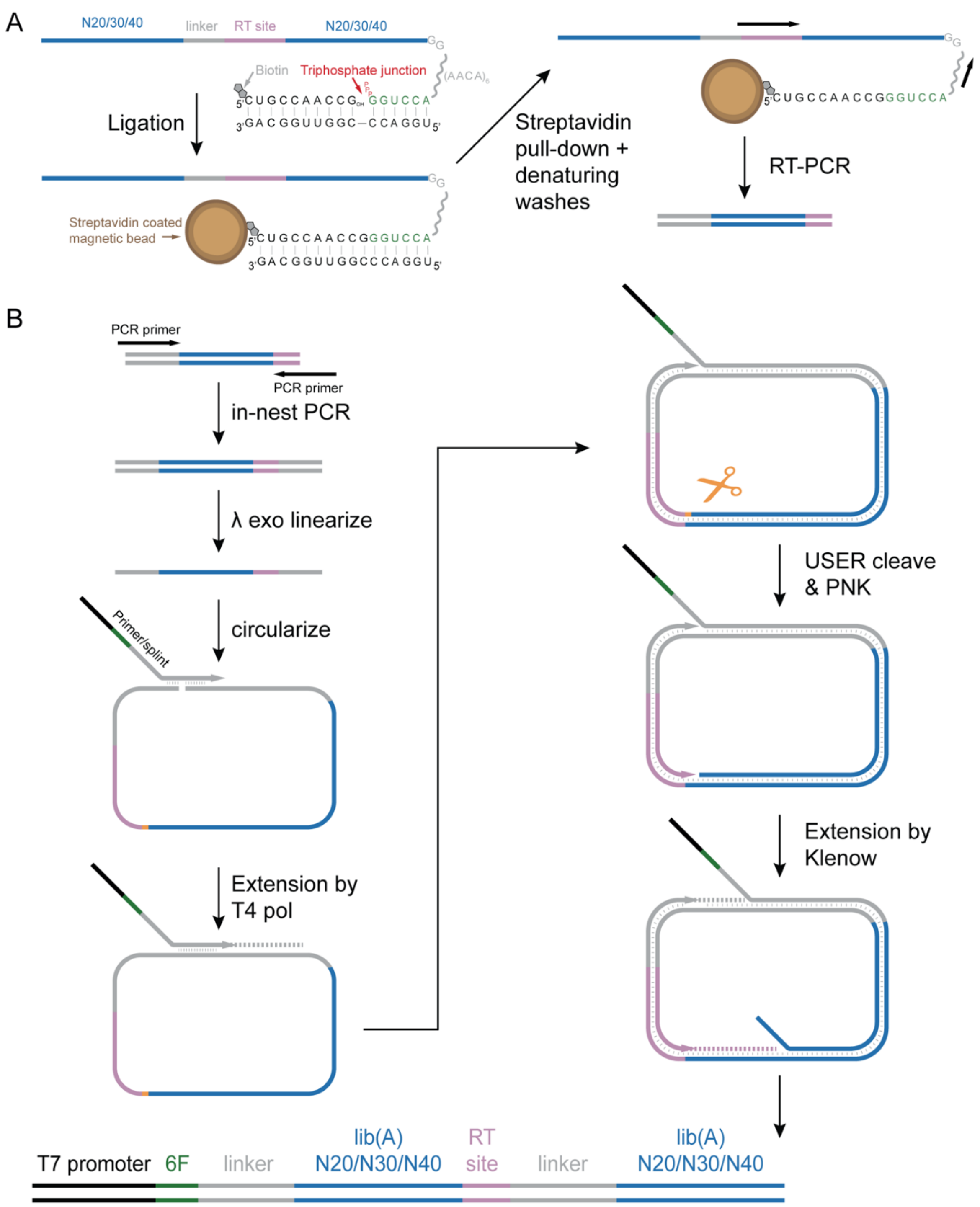

Fig. S1. Homodimeric construct formation and selection scheme.

(**A**) A homodimeric RNA library containing two copies of each random sequence (blue) is challenged to catalyze templated RNA ligation. The two copies are connected via a linker sequence (gray) and a reverse transcription primer binding site (RT site). Active members of the library are selectively recovered via streptavidin pull-down and a single monomer is amplified via RT-PCR. (**B**) The DNA of a single monomer is amplified via PCR and the product is linearized via λ exonuclease digestion. The linear product is circularized using a DNA splint containing a 5'-overhang encoding the T7 promoter. The splint is then used as a primer to synthesize the

complementary strand to the circular template. T4 DNA polymerase is used to avoid strand displacement and rolling circle amplification. A nick is formed in the circular strand using USER enzyme at a pre-defined position containing deoxy-uracil. After dephosphorylation of this nick, Klenow DNA polymerase is used to extend at the two available primer sites, displacing the opposite strand, and generating the duplicated sequence of interest with a T7 promoter.

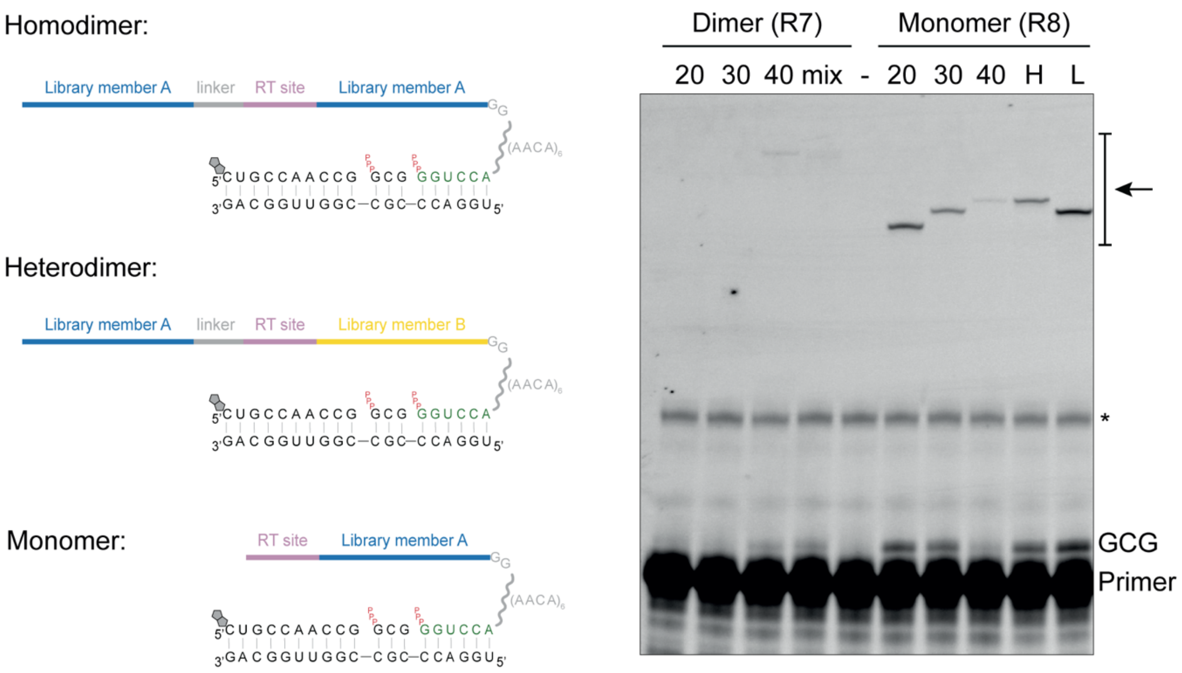

Fig. S2. Library activity as monomer or dimer.

(Left) Diagrams of three possible types of constructs are displayed. Homodimeric constructs contain two copies of the same library member. Heterodimeric constructs contain two library members differing in their sequence. Monomeric constructs have been truncated to only contain one library member and its RT site. (Right) Primer extension activity of the libraries as a dimeric construct in R7 compared with the libraries in monomeric construct in R8. Area where full length self-ligation is visible is indicated by an arrow. The asterisk indicates a reaction-independent anomalous primer migration band. Libraries of origin are annotated (see table S1 for more details). H/L consists in the "Mix" library's individual components after purification. Reaction conditions: 0.5 µM primer BCy3newP10, 0.5 µM template temp6FnewP10GCG, 5 µM pppGCG, 0.05% Tween 20, 200 mM KCl, 50 mM $MgCl_2$, 50 mM CHES-KOH, pH 9, 16 hours at -7 °C frozen.

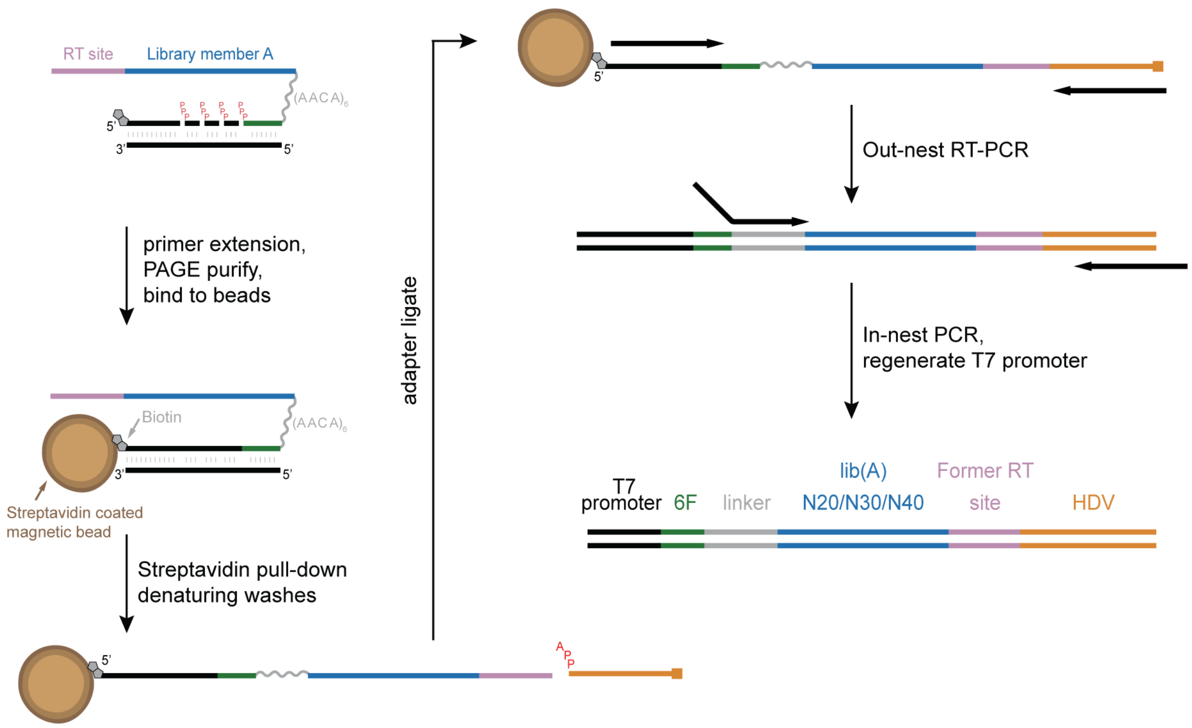

Fig. S3. Selection in monomeric form.
Diagram of the selection scheme used for the selection of a monomeric triplet polymerase ribozyme. An RNA construct containing the library of interest is incubated with a template, biotinylated primer and triplets. The 5' end of the selection construct contains 6 nucleotides that hybridize with the 5'-end of the template. Upon reaction, streptavidin beads are used to selectively recover the member of the library that carried out iterative triplet polymerization followed by ligation of the product to the hybridized region of the construct. Brief NaOH washes ensure recovery of solely the library members that are covalently bound to the biotin. Denaturing PAGE is used to select the correct size product. The recovered products are ligated to a DNA adapter, which provides a landing site for a primer in the subsequent reverse transcription. RT-PCR amplifies the recovered products, and a subsequent PCR is used to regenerate products suitable for transcription. The 3' end of this construct contains an HDV (Hepatitis Delta Virus) ribozyme (orange) that self-cleaves co-transcriptionally.

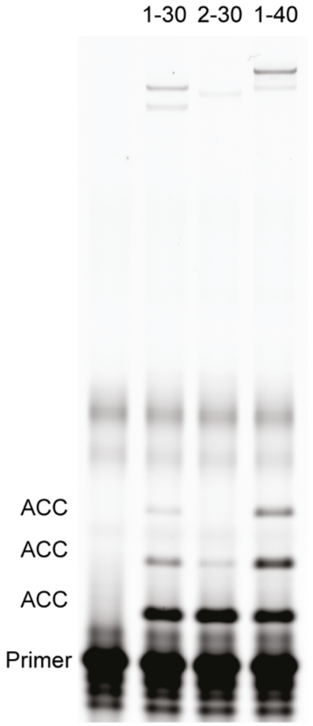

Fig. S4. Polymerization of ACC.
Iterative triplet polymerization by the clones displayed in (Fig. 1B) in the selection construct displayed in (Fig. 1A), with XXX=ACC and y=3. Reaction conditions: 50 nM ribozyme-substrate, 50 nM primer BCy3P10ga, 50 nM template temp6FP10gaACC3, 5 µM pppACC triplet, 0.05% Tween 20, 200 mM KCl, 50 mM $MgCl_2$, 50 mM CHES-KOH, pH 9, 3 days incubation at -7 °C frozen. Ribozymes are assayed *in cis* (see Figure 1C).

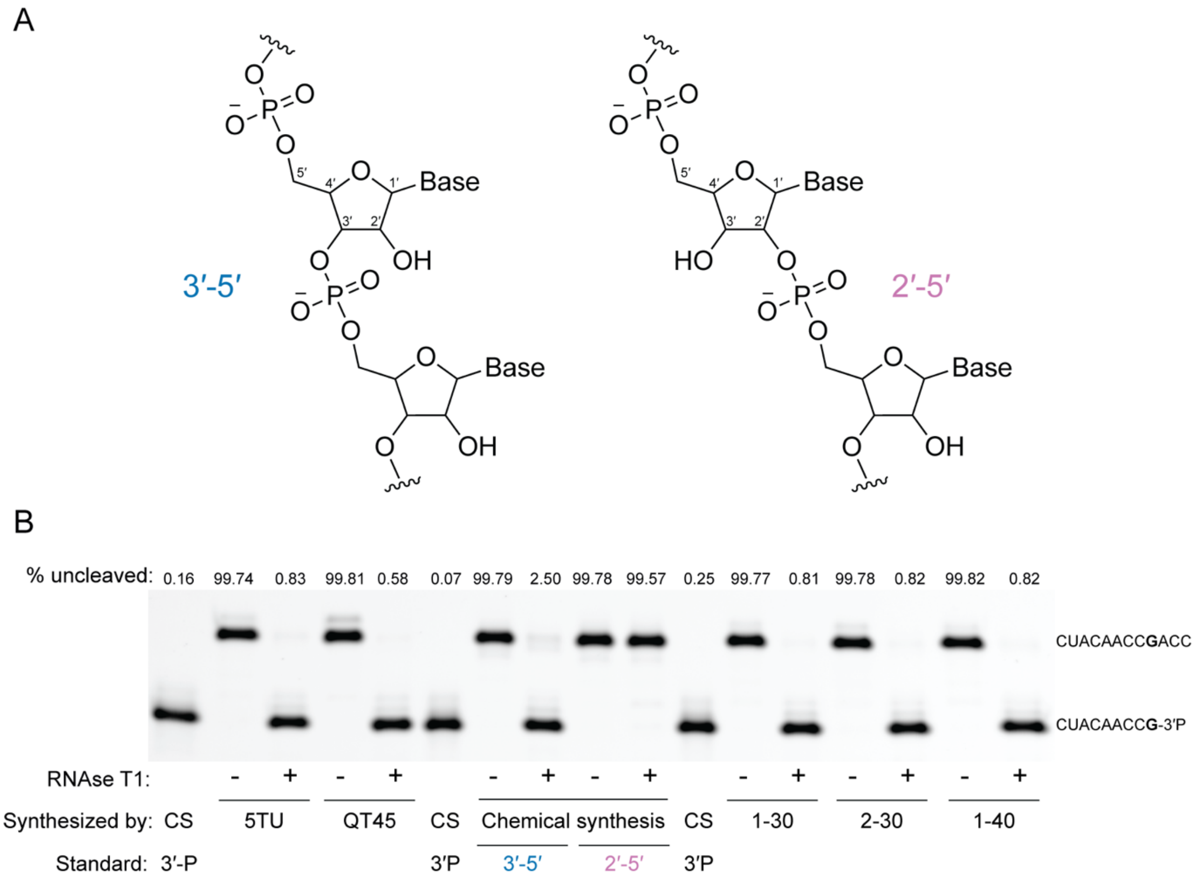

Fig. S5. Regiospecificity of ligation assessed via RNase T1 cleavage assay.
(**A**) Chemical structure of the 3′-5′ phosphodiester bond and the 2′-5′ phosphodiester bond. (**B**) Ligation products by various ribozymes (5TU, QT45, 1-30, 2-30, 1-40) of pppACC triplet to a primer (FBAP9) containing a single G at its 3' end are assayed for their susceptibility to RNase T1 cleavage compared to chemically synthesized (CS) standards (FBAP9_35ACC and FBAP9_25ACC). All ribozymes tested generated a RNAse T1 susceptible product, suggesting 3'-5' linkage. A 3' phosphorylated primer (FBAP9_3p) was used as a marker of correct digestion. RNase T1 was confirmed to cleave 3'-5' linked standards but not 2'-5' linked standards, which are resistant to RNase T1 cleavage. Chemically synthesized 3'-5' linked standards show incomplete cleavage compared to ribozyme synthesized products, presumably due to incomplete deprotection of the RNA during synthesis.

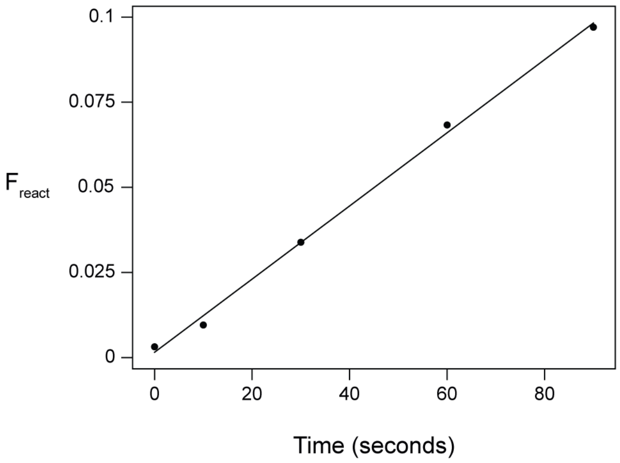

Fig. S6. Catalytic activity of QT51

Templated ligation of two RNA oligonucleotides in the format of Fig 1A, fraction of primer reacted is plotted over time in seconds. Reaction conditions: 0.1 µM ribozyme-substrate QT51_6F5L, 0.1 µM primer BCy3P10, 0.1 µM template temp6FF10, 500 mM $MgCl_2$, 250 mM CHES-KOH, pH 9, 0.05% Tween-20, 25 °C. The ribozyme is assayed *in cis* (see figure 1C). Reactions were carried out as duplicates and the mean is displayed. Fraction reacted over the first 10% (to approximate initial rates) of the reaction was fit by linear regression (solid line, $R^2 = 0.997$), and the rate was calculated from the slope.

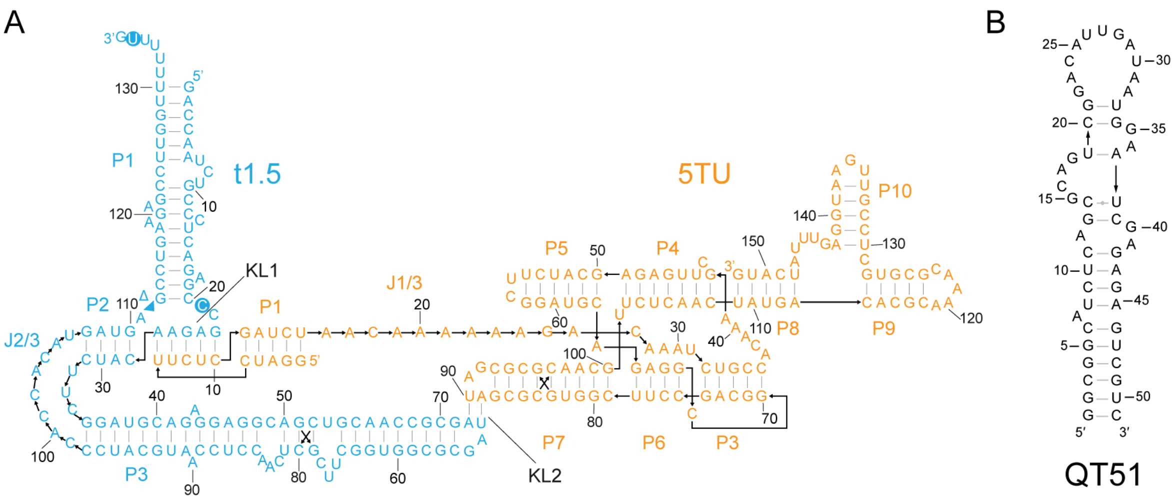

Fig. S7. Comparison of secondary structure and size of 5TU+t1.5 and QT51 ribozymes. (**A**) 5TU ribozyme with t1.5 cofactor, mutations from the ancestral t1 sequence indicated on the diagram. Figure adapted from (*31*). (**B**) QT51 ribozyme predicted secondary structure.

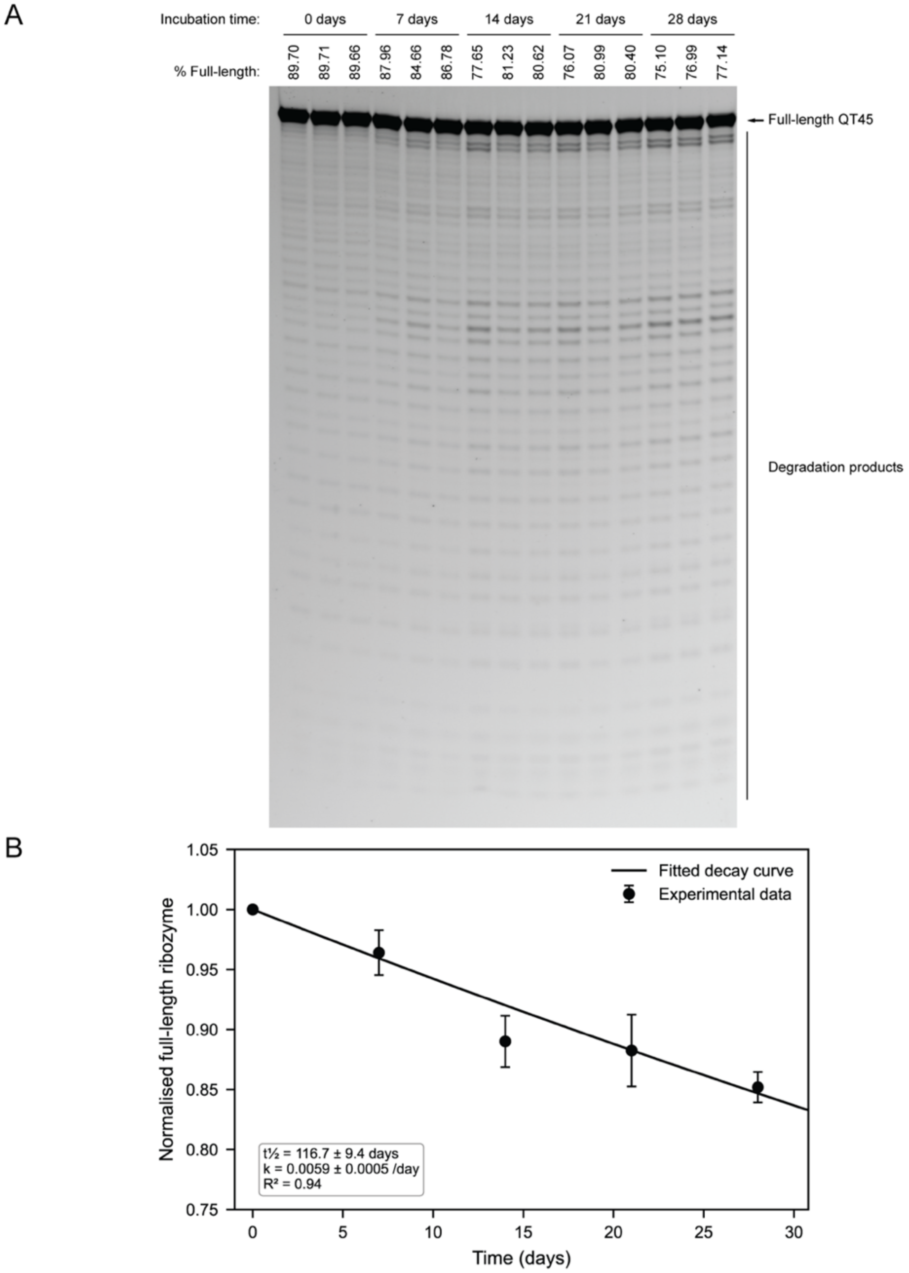

Fig. S8. Degradation rate of QT45. (**A**) Degradation time-course of FITC-QT45 observed via denaturing PAGE. FITC-QT45 ribozyme was incubated in 50 mM CHES-KOH pH 9, 50 mM $MgCl_2$, 0.05% Tween-20 reactions frozen and incubated at -7 °C for the time indicated. The full-length band intensity percentage over total bands intensity is indicated above each lane. (**B**) Experimental data from (A), normalized to starting amounts, with error bars displaying s.d. of the three replicates. Experimental data was fitted to an exponential decay function, suggesting a decay constant of 0.0059 (R^2 = 0.94) per day, and a half-life of ~117 days. Curve fitting of QT45 degradation kinetics was conducted in a similar way as hammerhead ribozyme cleavage (described in Section 4.1), also following first-order kinetics of the same form. Curve fitting was also performed using scipy.optimize.curve_fit with weighted least squares, where weights were derived from the standard deviation of three technical replicates.

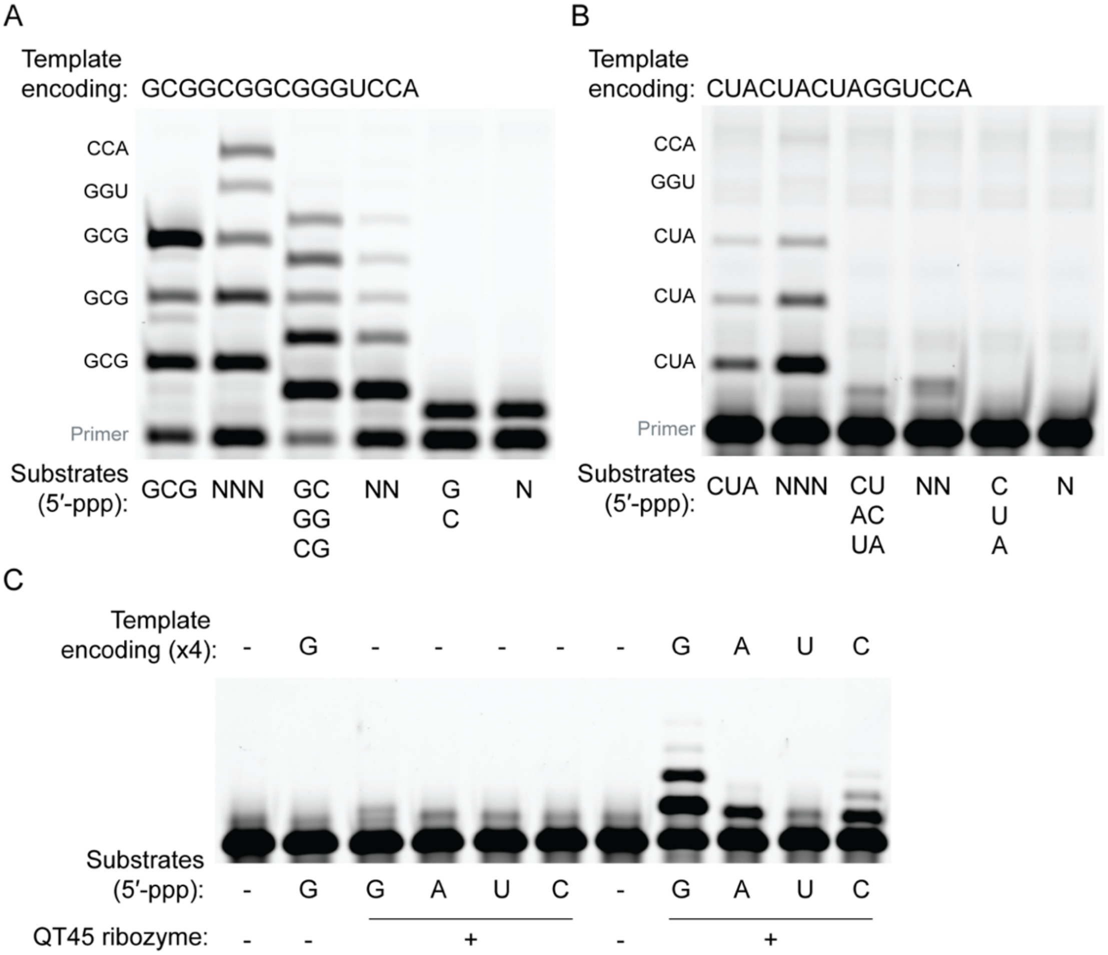

Fig. S9. QT45 polymerase activity using trinucleotide, dinucleotide, or mononucleotide substrates.
(**A**) Primer extension on template encoding 3GCG triplets followed by GGU, CCA, in the presence of either trinucleotides, dinucleotides, or mononucleotides as substrates. Reaction conditions: 0.25 µM primer (BCy3P10ga), 0.25 µM template (temp6FP10gaGCG3), 0.25 µM QT45, 50 mM $MgCl_2$, 200 mM KCl, 50 mM CHES-KOH, pH 9, 0.05% Tween-20, -7 °C frozen, 7 days. Triplet concentration: 1.75 µM each, dinucleotide concentration: 17.5 µM each, mononucleotide concentration: 175 µM each. NNN/NN/N indicates that all combinations were provided. (**B**) Primer extension on template encoding 3CUA triplets followed by GGU, CCA, in the presence of either trinucleotides, dinucleotides, or mononucleotides as substrates. Reaction conditions: 0.25 µM primer (BCy3P10), 0.25 µM template (temp6FP10CUA3), 0.25 µM QT45, 50 mM $MgCl_2$, 200 mM KCl, 50 mM CHES-KOH, pH 9, 0.05% Tween-20, -7 °C frozen, 7 days. Substrate concentrations (each): triplet 1.75 µM, dinucleotide 17.5 µM, mononucleotide 175 µM. NNN/NN/N indicates that all combinations were provided. (**C**) Primer extension reactions on template encoding 4 repeats of the same mononucleotide, followed by UCAU. Reactions were carried out in the presence of the encoded mononucleotide. Reaction conditions: 0.25 µM primer (F10), 0.25 µM template (tP10_44X_AGUA, X=A/C/G/U), 175 µM each mononucleotide triphosphate, 2.5 µM QT45, 50 mM $MgCl_2$, 50 mM CHES-KOH, pH 9, 0.05% Tween-20, -7 °C frozen, 15 days.

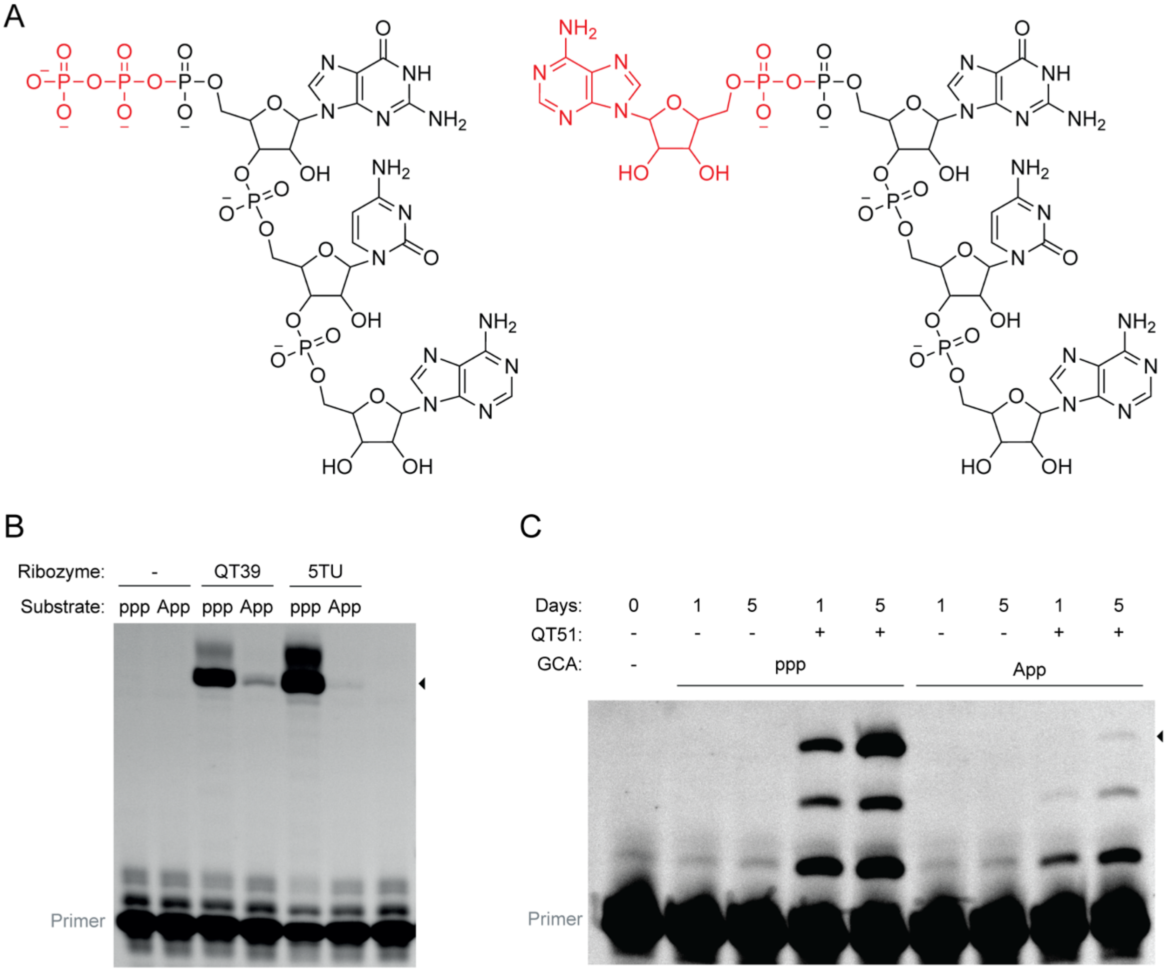

Fig. S10. Promiscuity in substrate leaving group.
(**A**) Chemical structures of triphosphorylated GCA triplet (left) and adenylated GCA triplet (right). (**B**) Comparison of ligation activity between triphosphorylated and adenylated (App) downstream substrates by QT or 5TU ribozyme. Reaction conditions: 0.25 µM primer F10, 0.25 µM template tempF10Ltest, 0.25 µM ribozyme (QT39 or 5TU+t1.5), 0.25 µM substrate (pppLtest1 or AppLtest1), 50 mM $MgCl_2$, 50 mM CHES-KOH, pH 9, -7 °C frozen, 19 hours (**C**) Comparison of triplet polymerization activity of triphosphorylated triplet compared with adenylated triplet substrate by QT ribozyme. Reaction conditions: 0.25 µM primer (BCy3nnP12), 0.25 µM template (temp6FP12nn3GCA), 0.25 µM ribozyme (QT51), 0.25 µM triplet (pppGCA or AppGCA), 50 mM $MgCl_2$, 50 mM CHES-KOH, pH 9, incubated at -7 °C frozen for the time indicated.

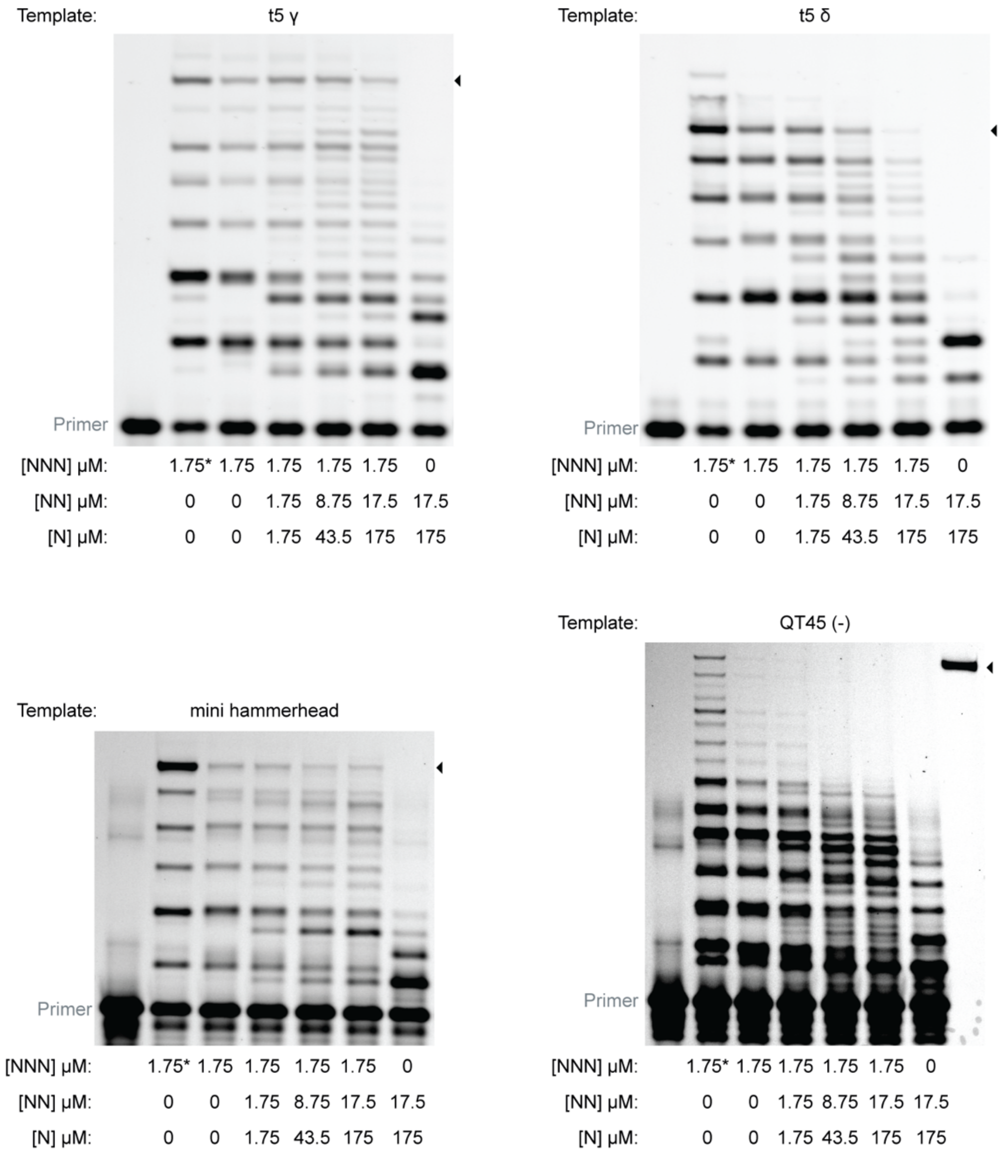

Fig. S11. QT ribozyme activity with varying distributions of mixed lengths substrate pools. Primer extension reactions were carried out either only with the triplets required for the synthesis (reactions indicated by asterisk*) or with mixes of all possible trinucleotides, dinucleotides, mononucleotides each at the concentrations indicated. Reactions conditions: 0.25 µM primer, 0.25 µM template indicated, 5 µM QT45, 0.05% Tween-20, 50 mM $MgCl_2$, 50 mM CHES-KOH, pH 9, -7°C frozen for 46 days. Primer/templates used were: Fgam7/t5gamtemp for t5γ, Fdel7/t5deltemp for t5δ, BCy3P10/tP10EuP1 for mini hammerhead and BCy3P10/t4msP10QT45 for QT45(-).

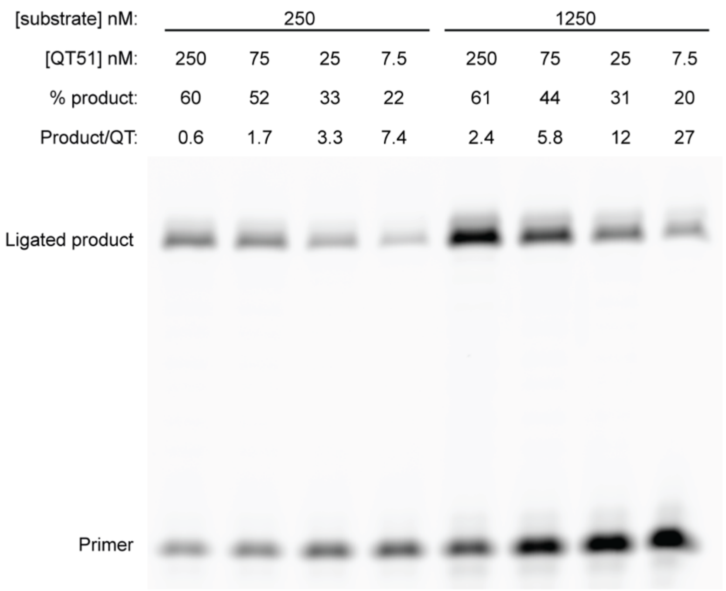

Fig. S12. Multi-turnover activity.
Ligation activity of QT51 over a range of ribozyme and substrate concentrations. Ligated product per QT ribozyme is shown. Reaction conditions (concentrations pre-freezing): 250 or 1250 nM primer/template/substrate (F10/tempF10Ltest/pppLtest1) as labelled, 250-7.5 nM QT51 ribozyme, 0.05% Tween-20, 50 mM $MgCl_2$, 50 mM CHES-KOH, pH 9, -7 °C frozen, 3 days.

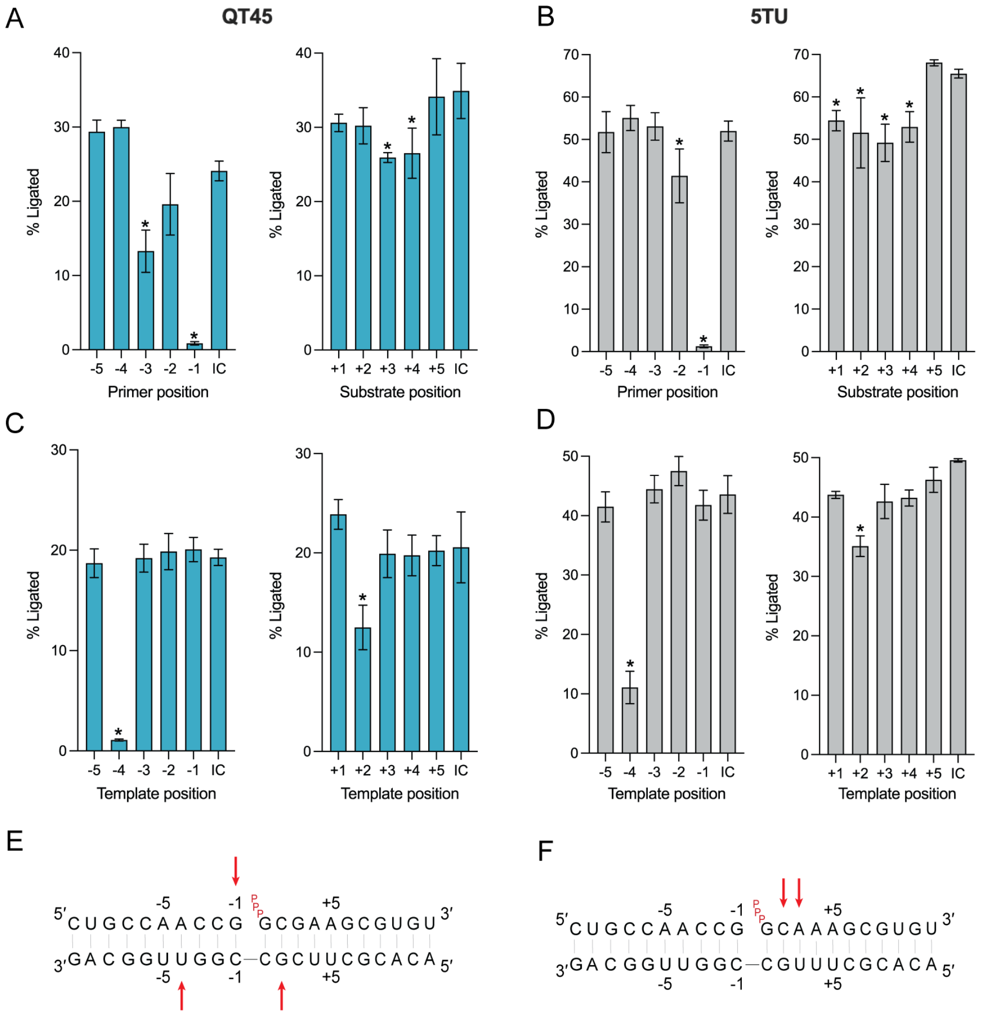

Fig. S13. Effect of 2'-hydroxyl substitutions in the template, primer, or substrate on ligation.

Percentage of product ligated by QT45 (teal) or 5TU+t1.5 (grey) in a single ligation junction where 2'-hydroxyl groups were individually substituted with 2'-deoxy, compared with an internal control (IC) of fully unsubstituted 2'-hydroxyl strand. Position of 2'-deoxy-substitutions indicated relative to a ligation junction (0). (**A**) Refers to 2'-deoxy substitutions on the primer and substrate oligonucleotide tested with QT45. (**B**) Refers to 2'-deoxy substitutions on the template tested with 5TU. (**C**) Refers to 2'-deoxy substitutions on the template tested with QT45. (**D**) Refers to 2'-deoxy substitutions on the primer and substrate tested with 5TU. (**E**) Primer-template-substrate junction used for testing primer and template position 2'-deoxy substitutions. Red arrows indicate substituted sites that affect ligation for both 5TU and QT45. (**F**) Primer-template-substrate junction used for testing substrate position 2'-deoxy substitutions. Red arrows indicate substituted sites that affect ligation for both 5TU and QT45. Reaction conditions: 0.1 µM primer F10, 0.1 µM template and substrate (see materials and methods for the exact sequences used), 0.1 µM ribozyme, 0.025% Tween-20, 50 mM $MgCl_2$, 50 mM CHES-KOH, pH 9, -7 °C frozen, 18 hours. Each substrate was tested in triplicates and the mean values for all positions were statistically analyzed using one-way ANOVA ($P < 0.05$) and a multiple comparison test to IC by the Dunnett Test; asterisks (*) mark the suppressed positions significantly different from IC. Displayed data are mean +/- SEM.

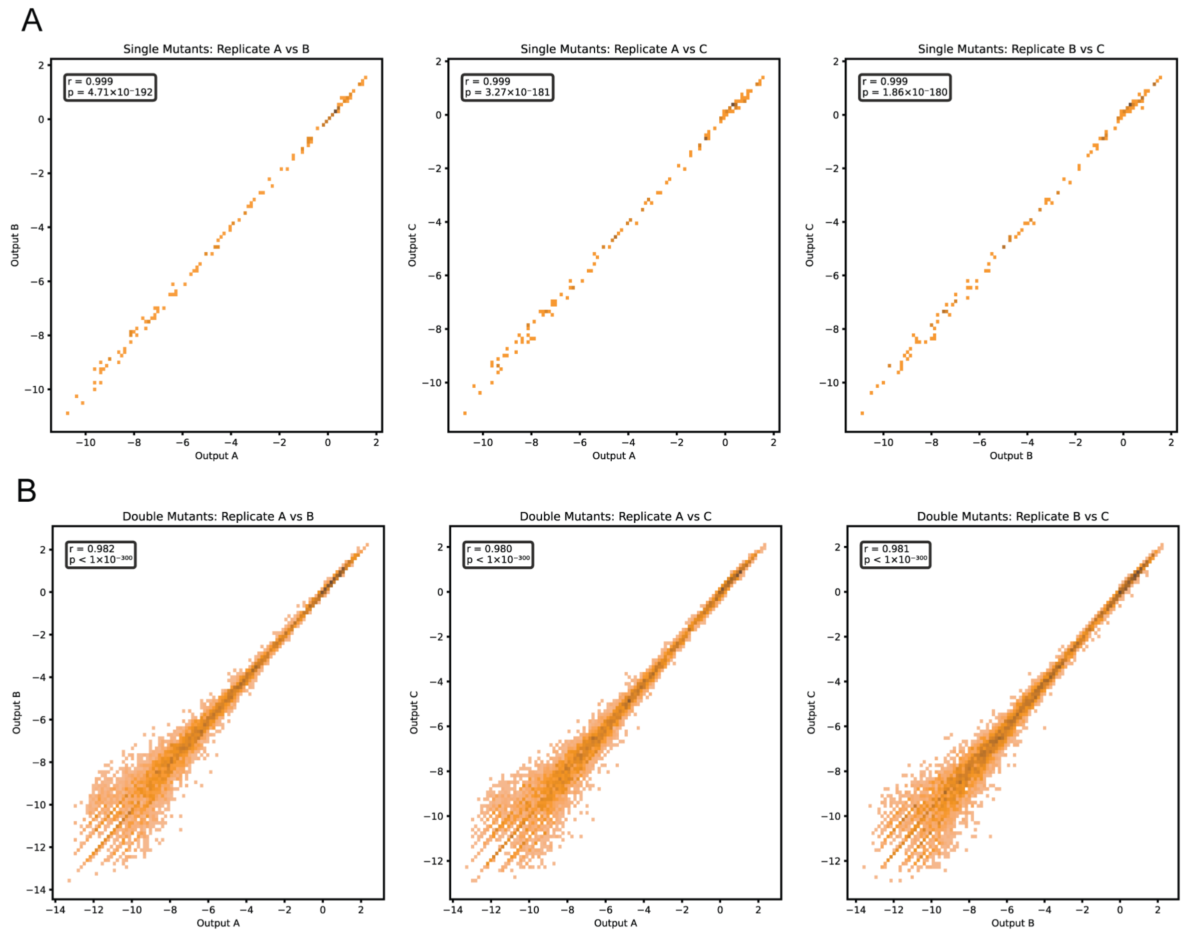

Fig. S14. Technical replicates (processed separately from primer extension onwards) show strong correlation in fitness measurements.

(A) Correlation between calculated log-transformed fitness values of single mutant genotypes across three replicates. Left: replicate A vs. B ($R = 0.999$, Pearson correlation coefficient). Middle: replicate A vs. C ($R = 0.999$, Pearson correlation coefficient). Right: replicate B vs. C ($R = 0.999$, Pearson correlation coefficient). **(B)** Correlation between calculated log-transformed fitness values of double mutant genotypes across three replicates. Left: replicate A vs. B ($R = 0.982$, Pearson correlation coefficient). Middle: replicate A vs. C ($R = 0.980$, Pearson correlation coefficient). Right: replicate B vs. C ($R = 0.981$, Pearson correlation coefficient).

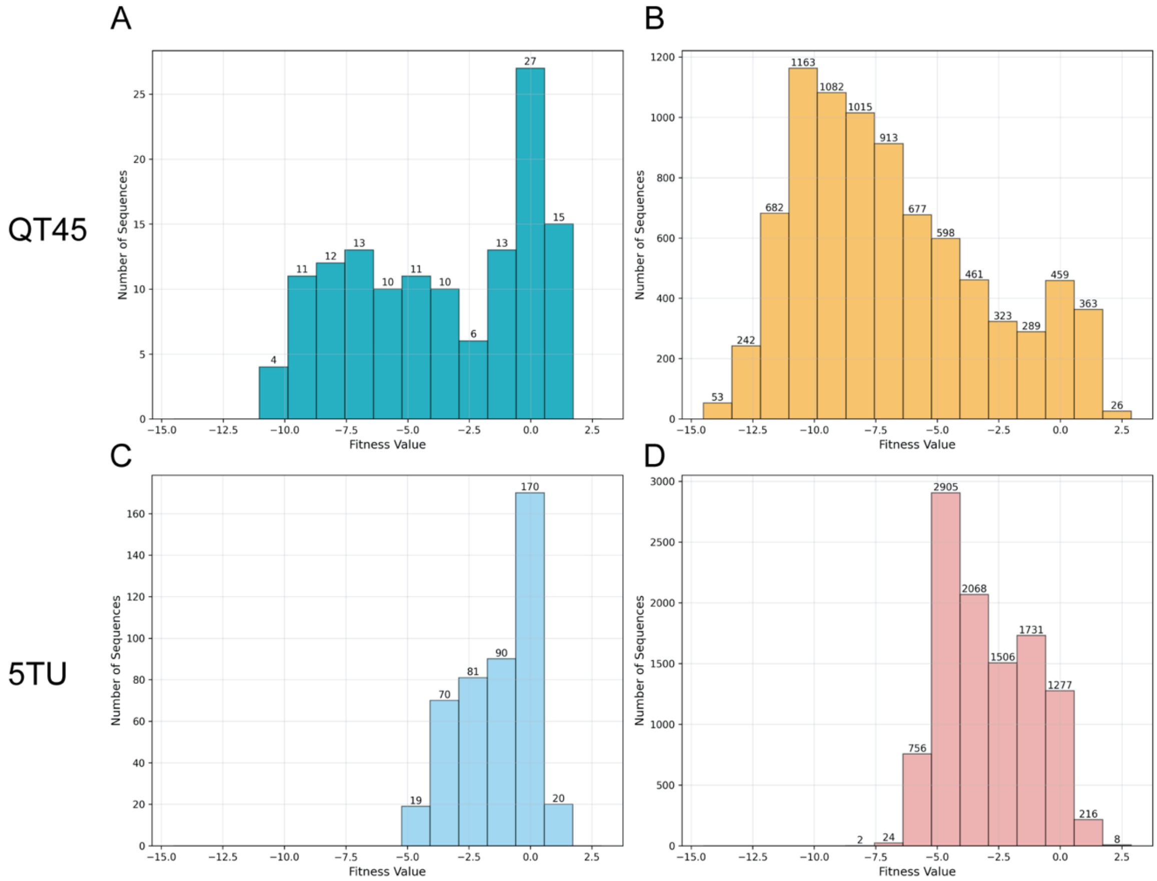

Fig. S15. Fitness value distributions suggest that QT45 shows greater sensitivity to mutations than 5TU.

(A) QT45 single mutant fitness value distribution (132 mutants) **(B)** QT45 double mutant fitness value distribution (8346 mutants). **(C)** 5TU single mutant fitness value distribution (450 mutants; data from (*31*)). **(D)** 5TU double mutant fitness value distribution (10493 mutants; data from (*31*)).

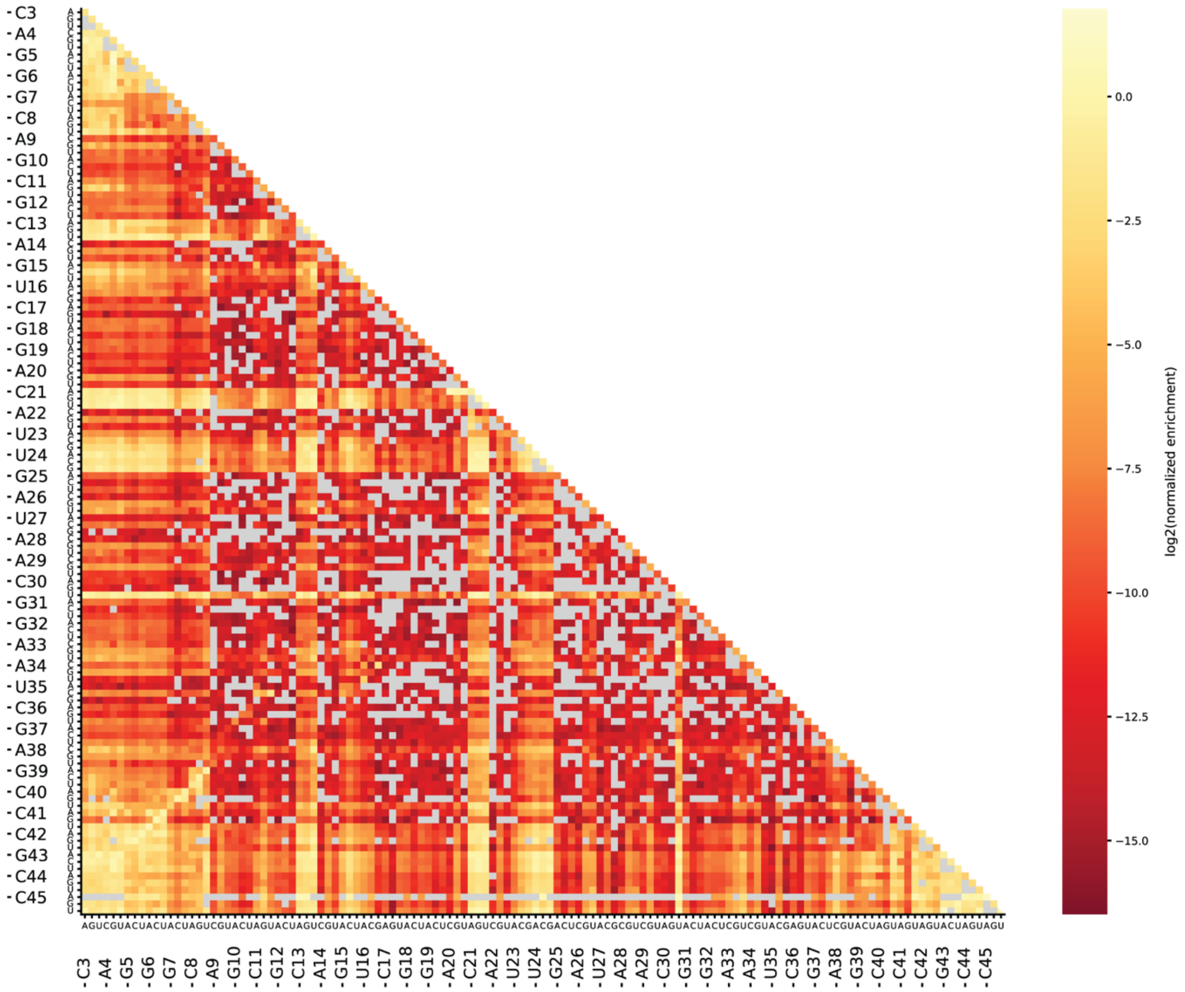

Fig. S16. Heatmap of primer extension activity on a 3 UGC template by all measured single mutants (129 mutants) and double mutants (6873 mutants) of the QT45 ribozyme.

The first constituent point mutation is indicated on the x-axis and the second mutation on the y-axis. Double mutants from the QT45MO10_dG1_iC45 register were selected from the input library comprising different shifted registers for fitness calculations after sequencing data filtering. Missing data points are shown in gray.

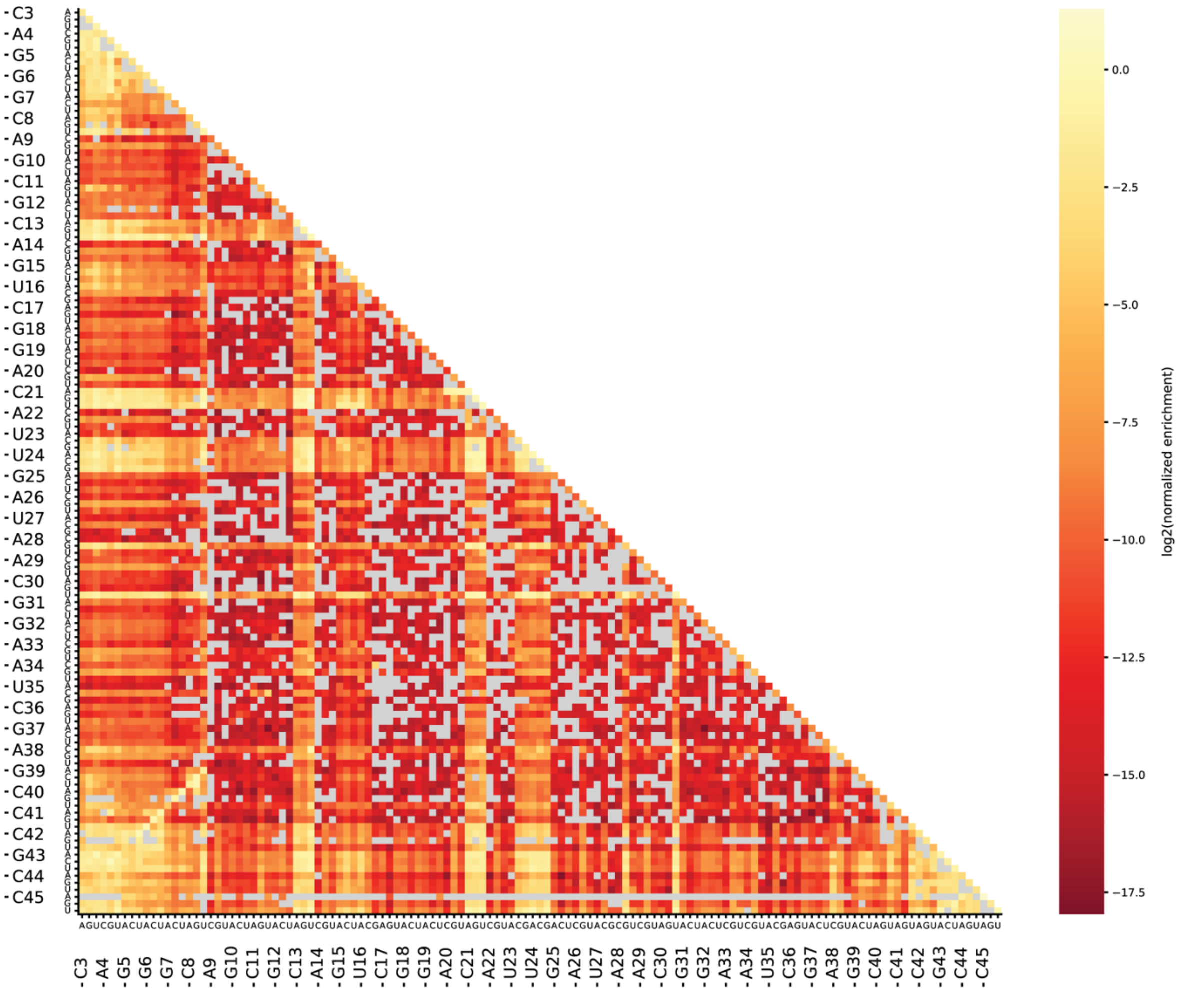

Fig. S17. Heatmap of primer extension activity on a 12 CUA template by all measured single mutants (129 mutants) and double mutants (6711 mutants) of the QT45 ribozyme.

The first constituent point mutation is indicated on the x-axis and the second mutation on the y-axis. Double mutants from the QT45MO10_dG1_iC45 register were selected from the input library comprising different shifted registers for fitness calculations after sequencing data filtering. Missing data points are shown in gray.

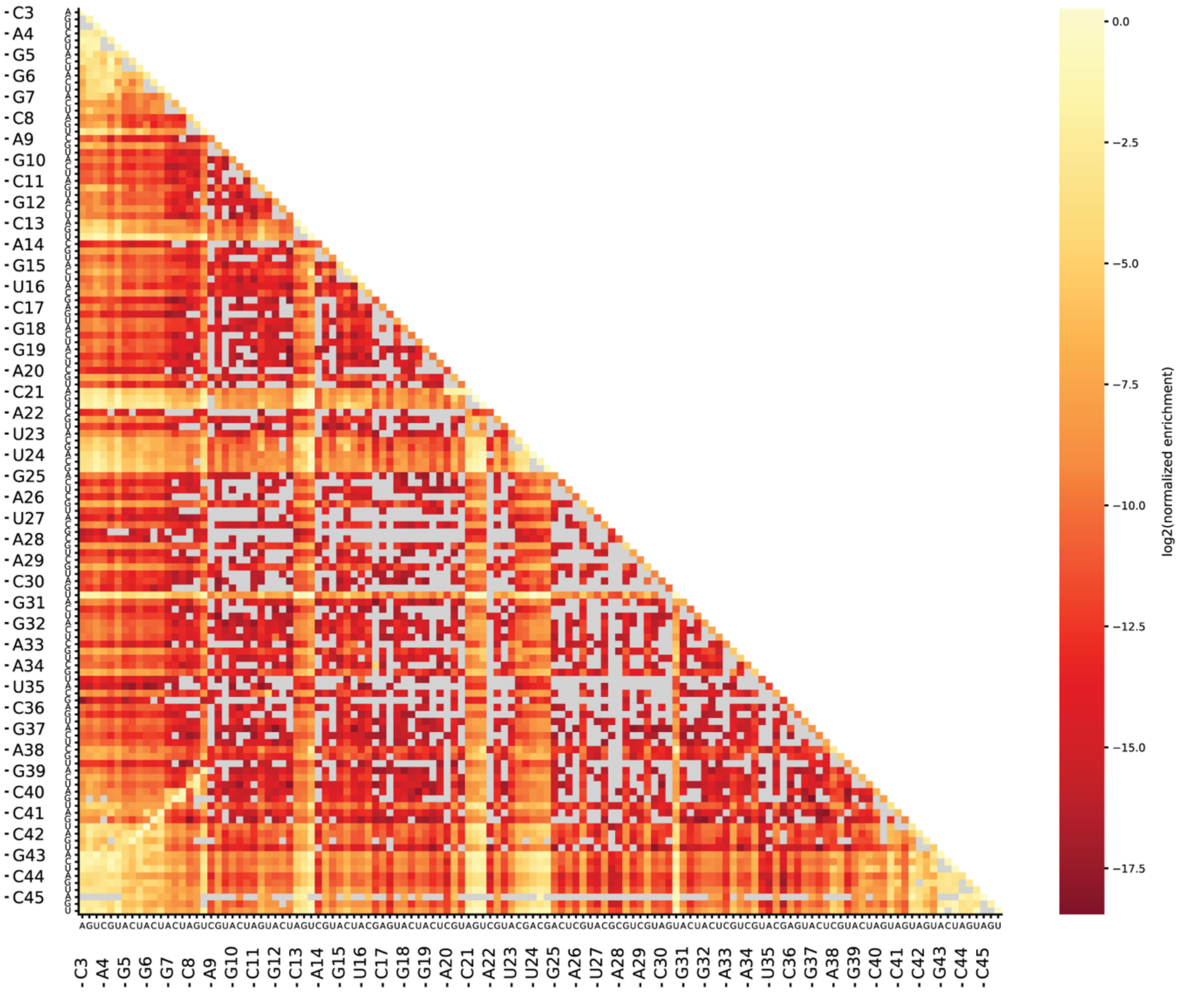

Fig. S18. Heatmap of primer extension activity on a 3 AUA template by all measured single mutants (129 mutants) and double mutants (6453 mutants) of the QT45 ribozyme.

The first constituent point mutation is indicated on the x-axis and the second mutation on the y-axis. Double mutants from the QT45MO10_dG1_iC45 register were selected from the input library comprising different shifted registers for fitness calculations after sequencing data filtering. Missing data points are shown in gray.

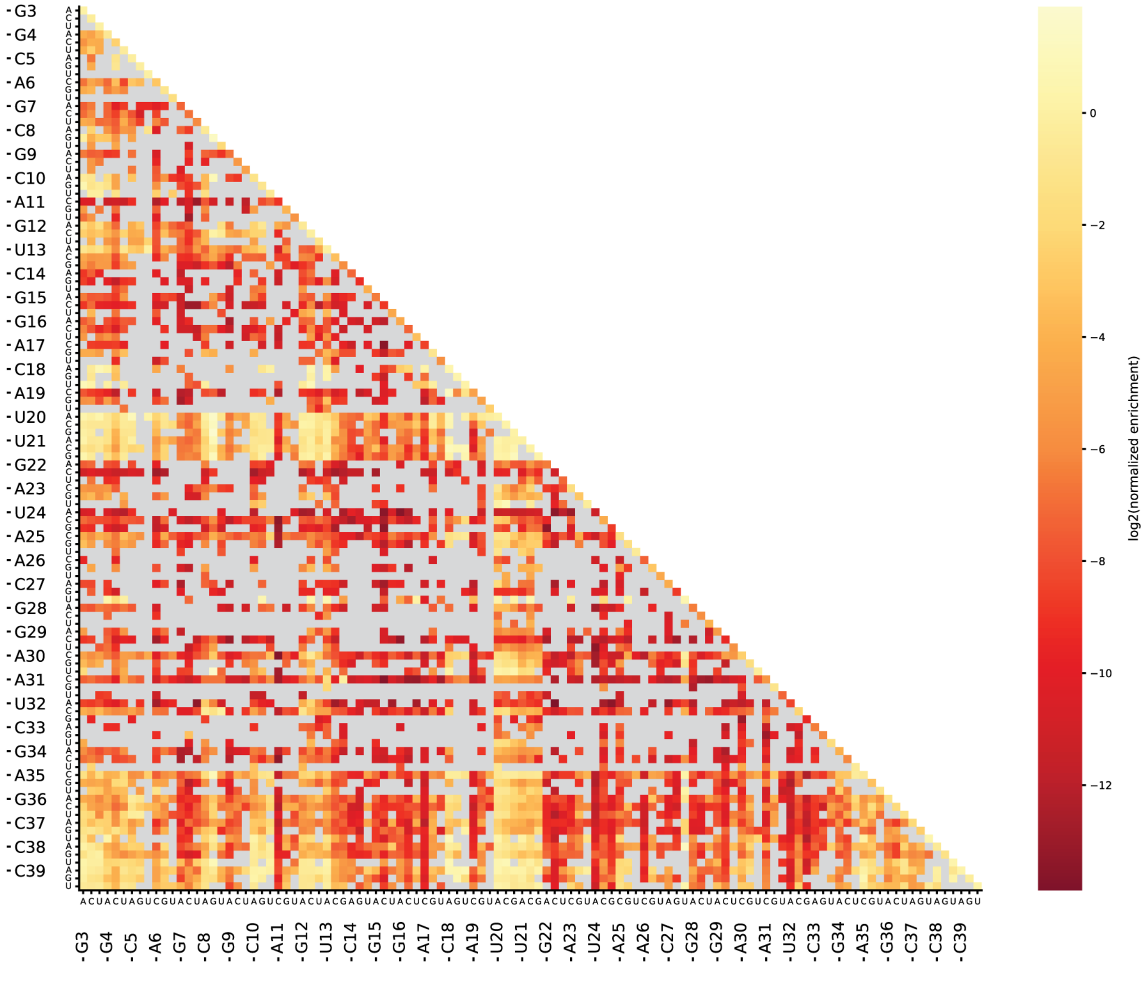

Fig. S19. Heatmap for all measured single and double mutants of the QT39 ribozyme. Heatmap for all measured single and double mutants of the QT39 ribozyme, with the first constituent point mutation indicated on the x-axis and the second one on the y-axis. Extension reactions on 3CUA template were done in triplicate and the mean fitness values were plotted. Fitness values are log-transformed enrichment values normalized to wildtype. Consequently, wild-type QT45 has a fitness of 0, while less active mutants have fitness values less than 0 and more active mutants have fitness values more than 0. As QT39 is a variant of QT45 that maintains the same core with a truncated stem, the QT39 heatmap shows similar trends as the QT45 heatmap (Fig. 2D).

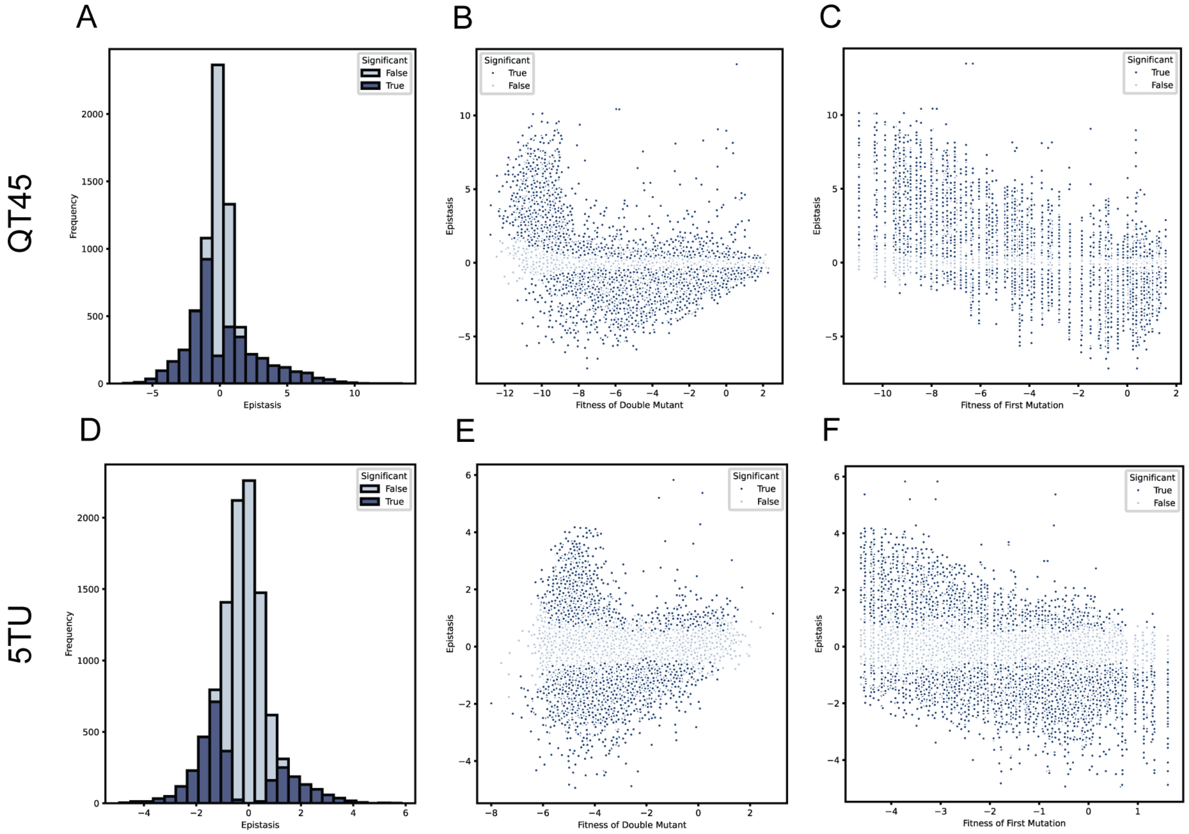

Fig. S20. QT45 epistasis is biased towards negative values, but less so than 5TU.
Top: QT45 epistasis distributions (7212 double mutants). Significant epistasis values are colored in dark blue (false discovery rate, 5.0%); non-significant epistasis values are in light blue. **(A)** Distribution of epistasis in QT45 double mutants. **(B)** Range of mean epistasis value between first and second mutations increases with decreasing fitness of the double mutant in QT45. **(C)** Mean epistasis value between first and second mutations increases with decreasing fitness of the first mutation in QT45. Bottom: 5TU epistasis distributions reproduced from (*31*) (10493 double mutants). Significant epistasis values are colored in dark blue (false discovery rate, 11.0%); non-significant epistasis values are in light blue. **(D)** Distribution of epistasis in 5TU double mutants. **(E)** Range of mean epistasis value between first and second mutations increases with decreasing fitness of the double mutant in 5TU. **(F)** Mean epistasis value between first and second mutations increases with decreasing fitness of the first mutation in 5TU.

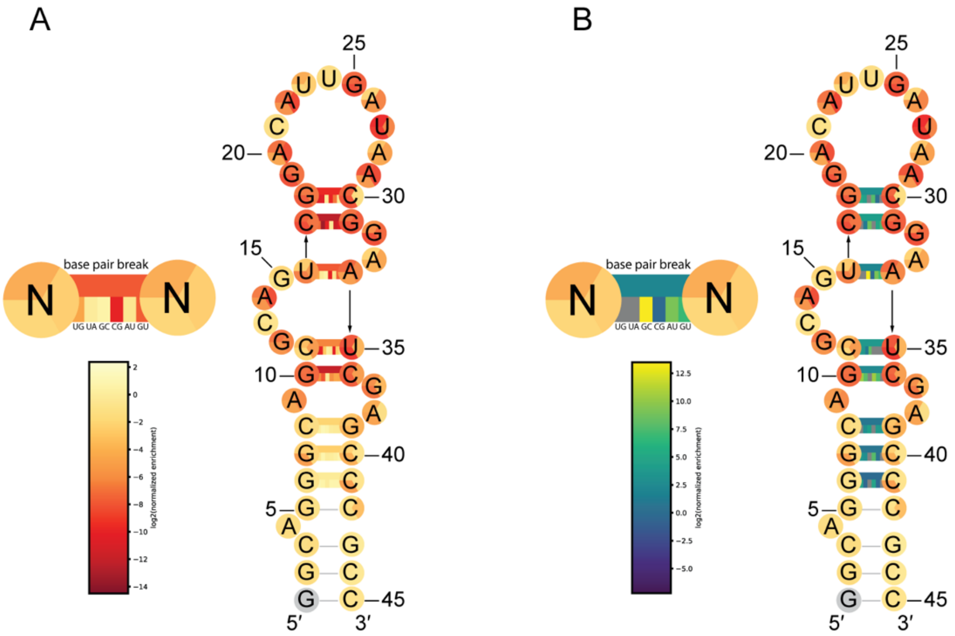

Fig. S21. Overview of evidence for base pairing in QT45.
Predicted secondary structure of QT45 ribozyme with nucleotide color corresponding to fitness for each of the three possible single mutations at each position, displayed in the same order as the three representative mutations shown and colored as in Fig. 2E. Base pair annotations are provided, where the upper rectangle connecting two bases shows the average fitness or epistasis of measured base pair-breaking double mutations. For canonical base pairs, the inner small rectangles show (from left to right) the fitness or epistasis of mutations to base pairs UG, UA, GC, CG, AU, GU. For the non-canonical base pair C11-U35, the base pairs shown are UG, UA, GC, UC, CU, CG, AU, GU. **(A)** Base pair annotations are colored according to fitness. Base pair-retaining mutations generally have higher fitness than base pair-breaking mutations. **(B)** Base pair annotations are colored according to epistasis. Base pair-retaining mutations generally show more positive epistasis compared to base pair-breaking mutations.

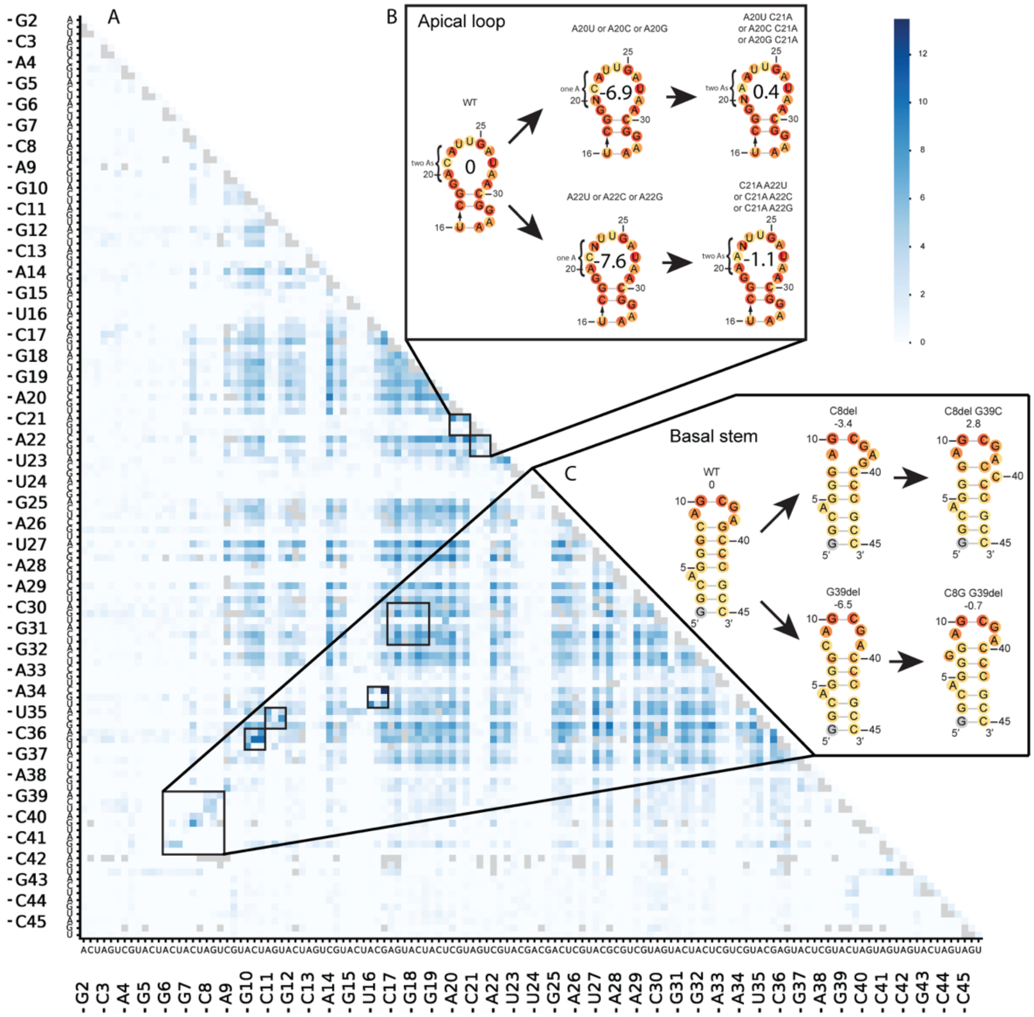

Fig. S22. Epistatic landscape reveals secondary structure interactions.

(A) Epistasis plot of primer extension activity on a 3 UGC template by all measured double mutants of the QT45 ribozyme (8346 mutants), with the first constituent point mutation indicated on the x-axis and the second mutation on the y-axis. Missing data points are shown in gray. Epistatic values range from -7.2 to 13.5, but the color scale is restricted to positive values (0 to 13.5) to emphasize positive epistatic interactions that identify base-pairing positions. **(B)** Examples of rescue interactions in the apical loop. Part of the secondary structure is shown and colored as in Fig. 2E. Numbers correspond to average fitness values of depicted mutants. Mutation of A20 or A22 greatly reduces activity, but this is rescued by the second mutation C21A. **(C)** Examples of rescue interactions in the basal stem. Disruption of the C8-G39 base pair through deletion of either base is detrimental to activity, but a similar configuration can be restored with a single mutation. Other boxed regions without panels highlight base pairs analyzed in Tables S3, S4, and S5.

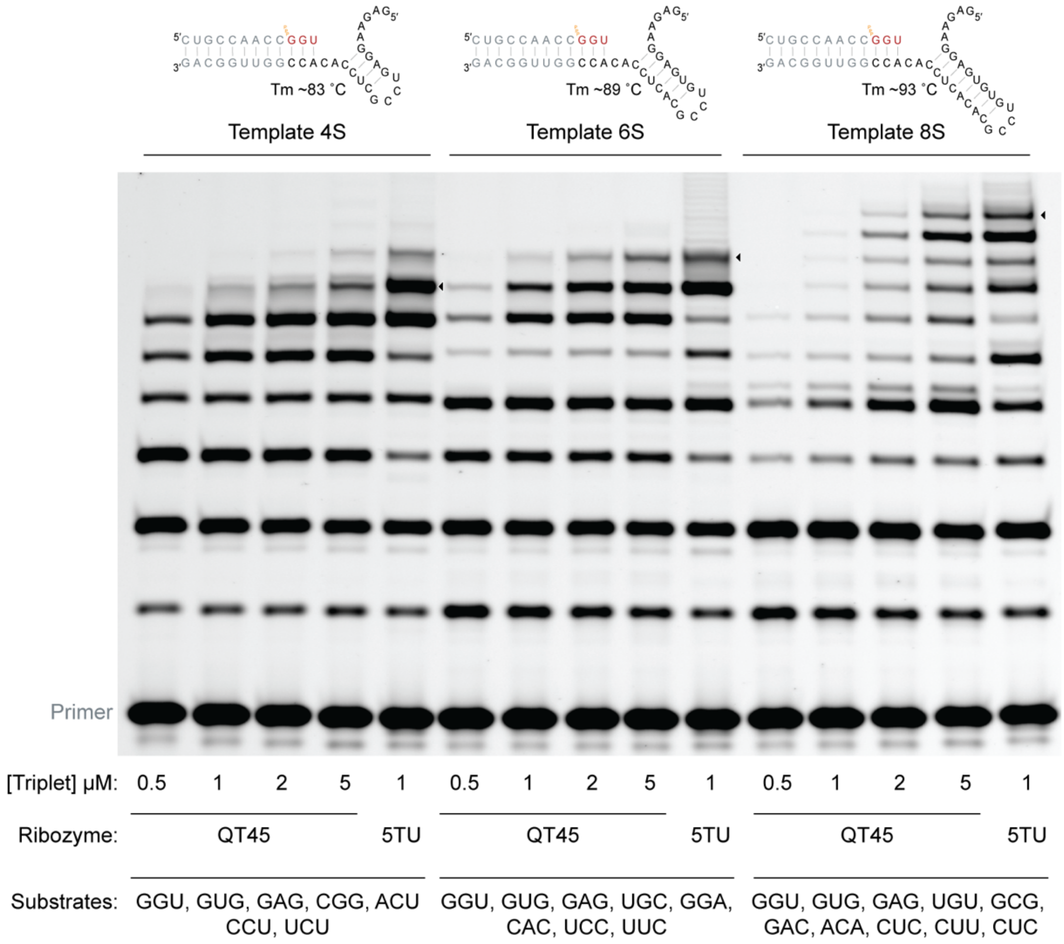

Fig. S23. QT ribozyme-catalyzed copying of stem-loop containing templates.
Copying of three templates containing increasingly stable secondary structures. Predicted stem-loop secondary structures and T_M displayed on top. Extension reactions were carried out with increasing concentrations of triplets (as indicated at the bottom). Increasing triplet concentration favored strand invasion and synthesis of structured templates. 5TU controls were used as marker of correct synthesis. Black triangles indicate full-length product migration. Reaction conditions: 0.25 µM primer (F9), 0.25 µM template (t4S/t6S/t8S, indicated above each lane), 1.25 µM QT45 ribozyme or 0.25 µM 5TU+t1.5 ribozyme, each triplet substrate at the concentration indicated below each lane, 0.05% Tween 20, 50 mM $MgCl_2$, 50 mM CHES-KOH, pH 9, -7 °C frozen for 35 days.

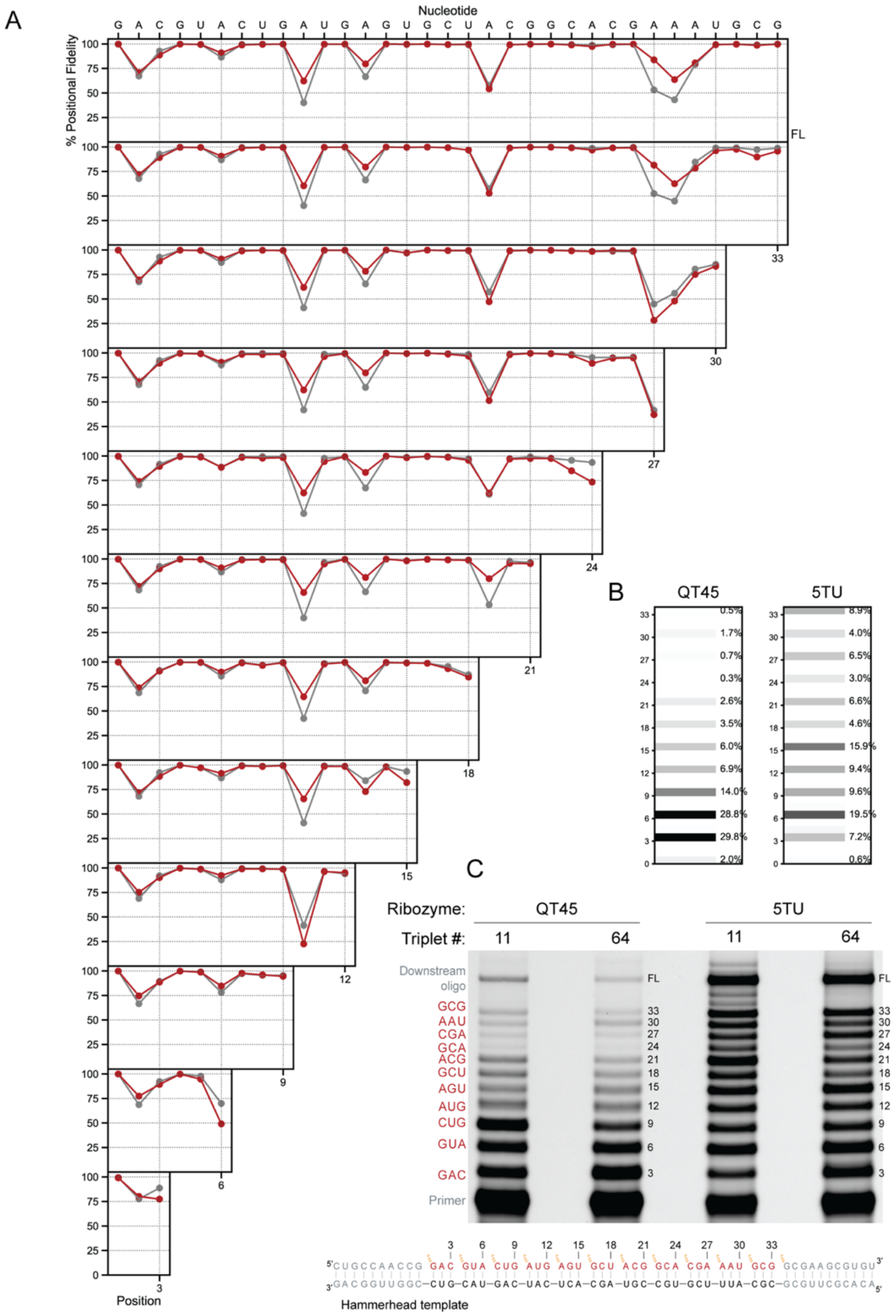

Fig. S24. Copying fidelity of intermediate products in the synthesis of the hammerhead ribozyme.

(**A**) Positional fidelity of the intermediate products in triplet register of the hammerhead ribozyme synthesis by QT45 shown in red, 5TU control shown in grey. (**B**) Percentage read abundance of the intermediate products in the hammerhead ribozyme synthesis displayed as a grayscale pseudogel. Recovered reads match expected triplet register. (**C**) Ribozyme catalyzed synthesis of the hammerhead ribozyme, reaction used for sequencing. Reaction conditions: 0.25 µM primer BCy3P10, 0.25 µM template tP10Lte_seq0HH, 2.5 µM QT45 or 0.25 µM 5TU+t1.5, 1.25 µM each defined triplet or each of the 64 possible triplets (NNN), 0.25 µM downstream oligo pppLtest1, 0.05% Tween 20, 50 mM $MgCl_2$, 50 mM CHES-KOH, pH 9, 65 days at -7 °C frozen.

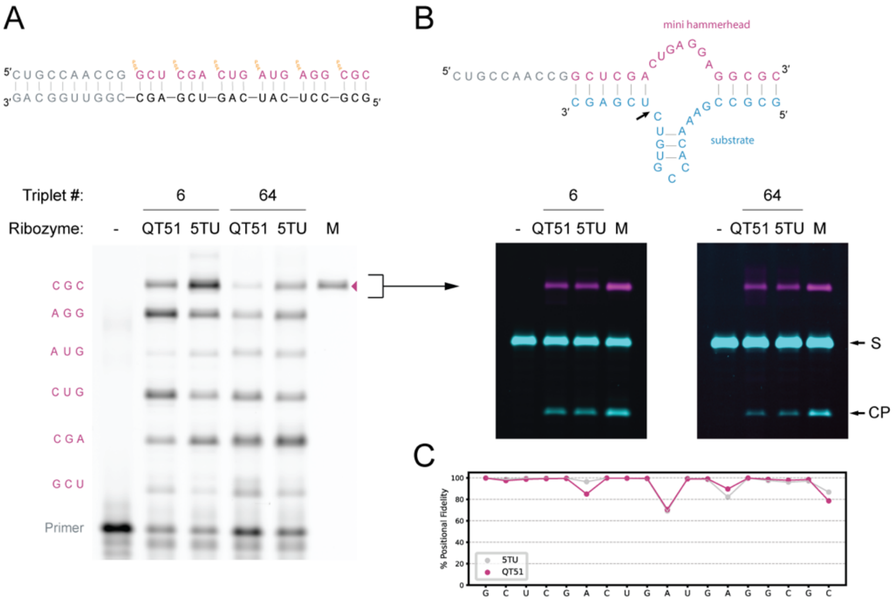

Fig. S25. QT ribozyme-catalyzed synthesis of an active ribozyme fragment (mini hammerhead).

(**A**) *Top:* diagram of primer, template and triplets necessary for the synthesis of the mini hammerhead ribozyme. *Bottom:* ribozyme catalyzed synthesis of the mini hammerhead ribozyme. Full-length product indicated by a magenta triangle. M indicates marker full-length product lane. Reaction conditions: 0.5 µM primer BCy3P10, 0.5 µM template tP10HHz, 5 µM QT51 or 0.5 µM 5TU+t1.5, 5 µM each defined triplet or 1.75 µM each of the 64 possible triplets (NNN), 0.05% Tween 20, 50 mM $MgCl_2$, 50 mM CHES-KOH, pH 9, 52 days at -7 °C frozen. (**B**) *Top:* diagram of the mini hammerhead ribozyme (magenta) in complex with its substrate (cyan). The cleavage site is indicated by an arrow. *Bottom:* cleavage activity of ribozyme-synthesized mini hammerhead ribozymes, compared with protein polymerase-synthesized controls (M lane). Synthesis reaction of the hammerhead used for cleavage was carried out as in (A) but for 36 days. Cleavage reaction conditions described in materials and methods. (**C**) Positional fidelity of copying by QT51 shown in magenta, 5TU control shown in grey. Synthesis reaction was carried out as in (A) but for 28 days.

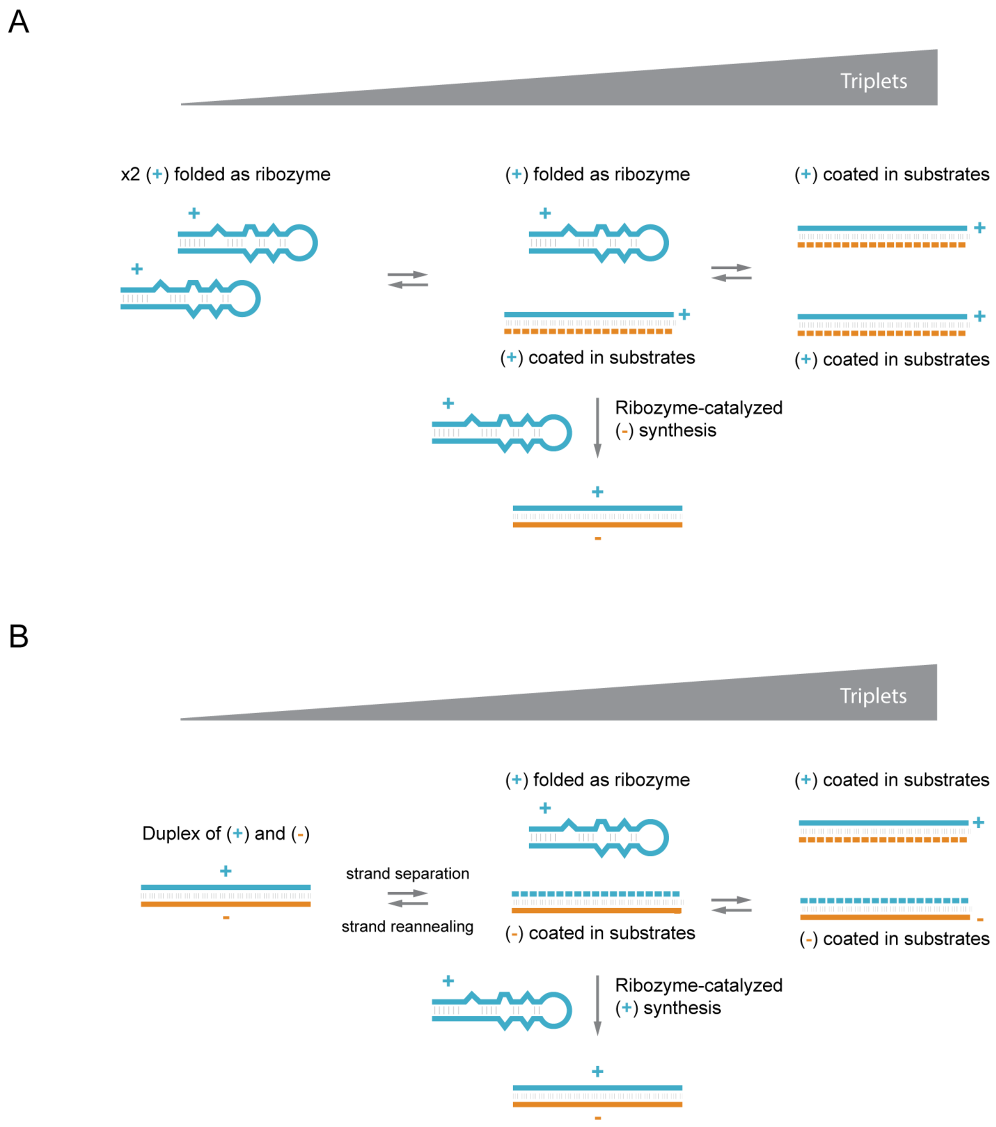

Fig. S26. Proposed role of triplet concentration in RNA self-replication.
(**A**) Effect of triplet concentration in (-) strand synthesis. At intermediate concentrations, triplets cooperatively unfold some ribozyme (+) strands, making them accessible as a template, while leaving other (+) strands folded as ribozymes. At higher concentrations all ribozymes are fully unfolded, and no (+) strand is available as catalyst. (**B**) Effect of triplet concentration on (+) strand synthesis. Upon strand separation at optimal triplet concentration, (+) strand folds as ribozyme and (-) strand is coated in substrates. At higher concentrations all ribozymes are fully unfolded, and no (+) strand is available as catalyst.

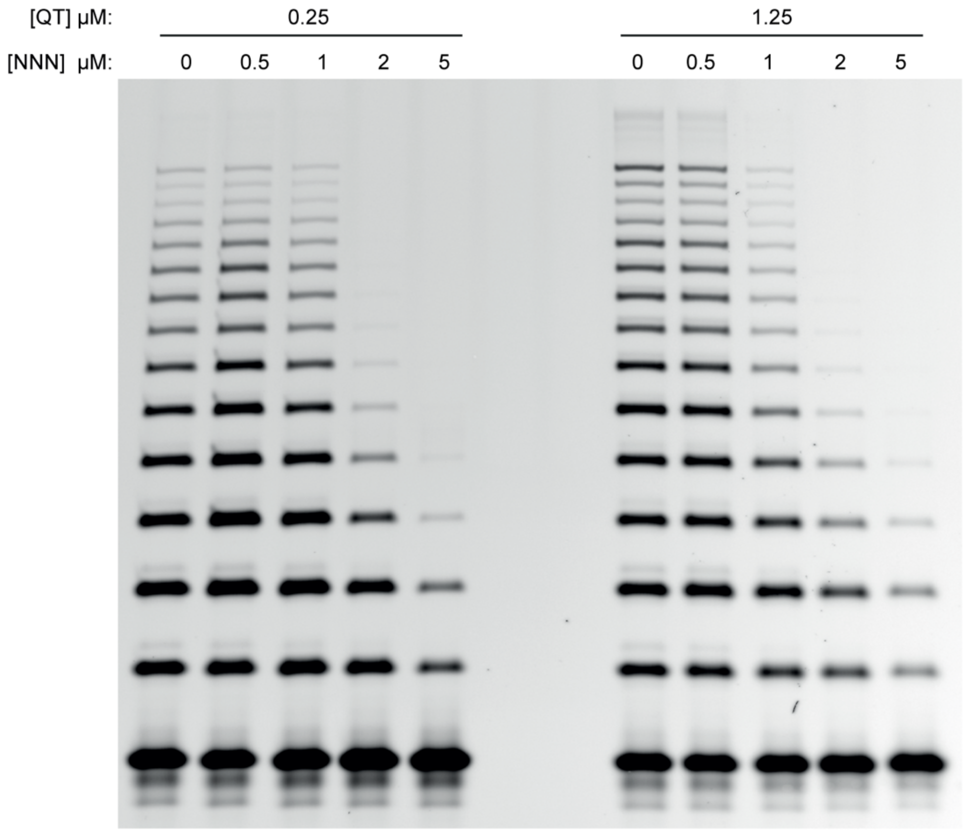

Fig. S27. Inhibition of ribozyme polymerase activity by high concentration of NNN. Synthesis of 42 nucleotide CGU repeat sequence by QT45 at varying concentrations of QT45 and NNN (equimolar mix of all possible triphosphorylated trinucleotides). Reaction conditions: 0.25 μM primer F10, 0.25 μM template tP1014CGU, 0.25 μM/1.25 μM ribozyme QT45, 5 μM pppCGU triplet, varying amount of each triplet in pppNNN (shown on gel), 50 mM $MgCl_2$, 50 mM CHES-KOH, pH 9, 3 days at -7 °C frozen.

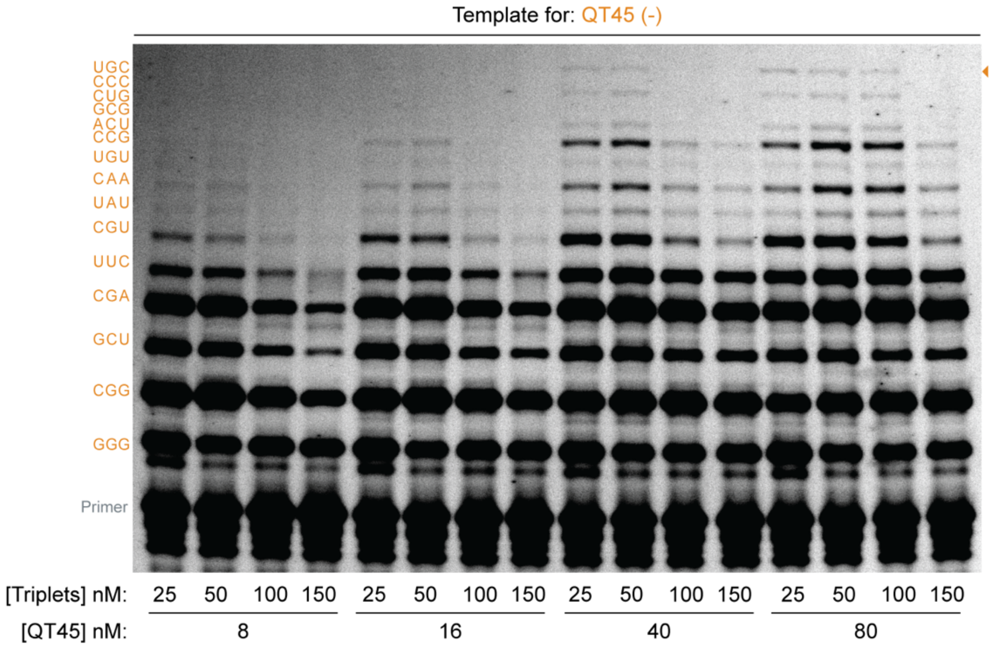

Fig. S28. Optimization of (-) strand synthesis conditions.

QT45-catalyzed synthesis of its own complementary strand (QT45(-)) starting with all 64 possible triplet substrates, at varying triplet and ribozyme concentrations. Full-length product indicated by a tan triangle. Reaction conditions: 8 nM primer BCy3P10, 23 nM template (t4msP10QT45), ribozyme and triplet concentrations annotated below the gel image, 0.01% Tween 20, 0.4 mM $MgCl_2$, 1.2 mM KCl, 1 mM CHES-KOH, pH 9, incubated for 26 days at -7 °C frozen.

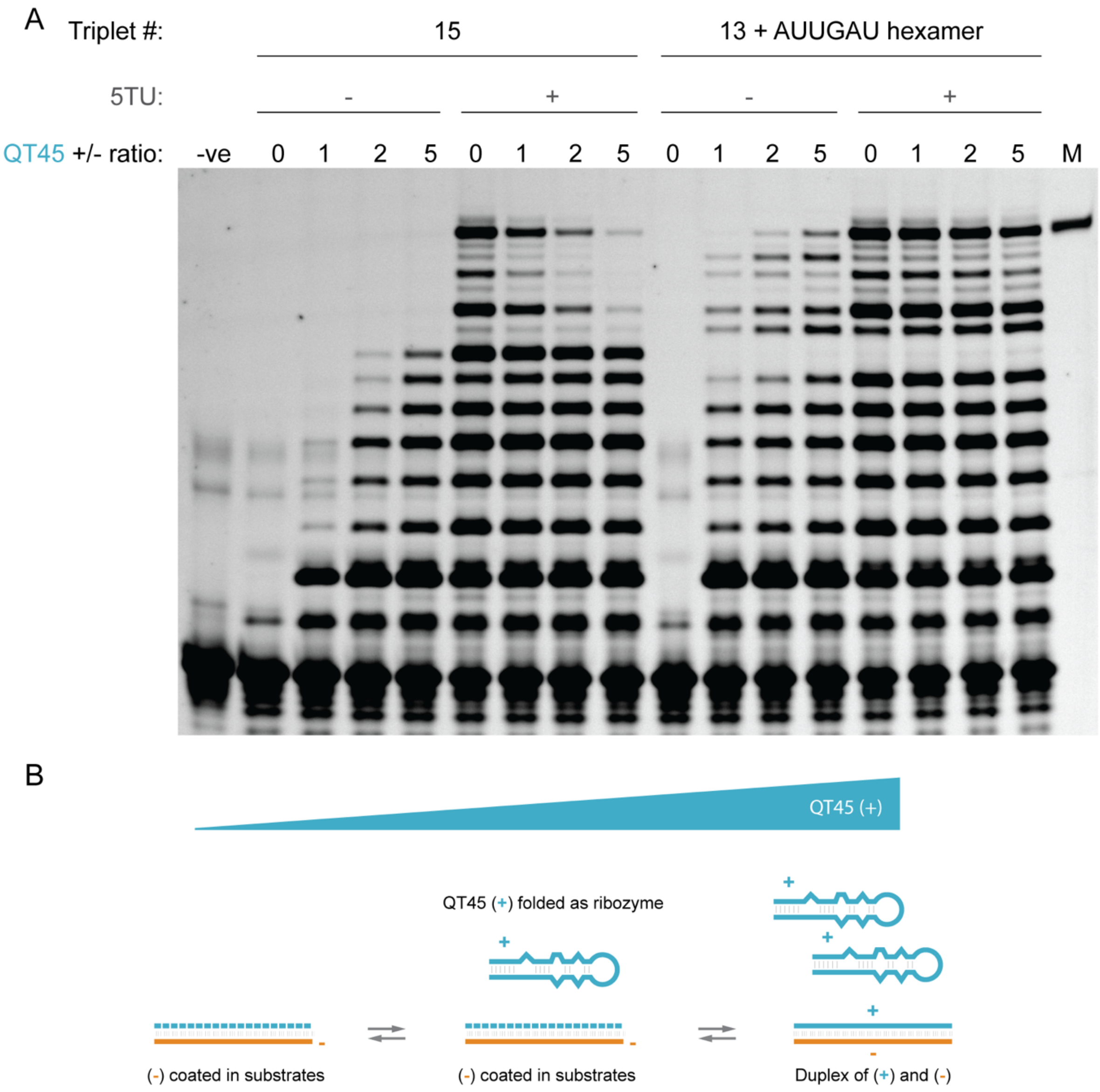

Fig. S29. Strand inhibition in QT45 (+) strand synthesis.
(**A**) QT45-catalyzed synthesis of itself (QT45(+)) with increasing ratio of (+) over (-) strand, compared with the same reaction supplemented with 5TU as an additional catalyst. Full-length is indicated by the synthetic marker on the rightmost lane. Reactions were performed using a mix of triplet substrates with or without the aid of one pre-formed hexamer. Reaction conditions: 8 nM primer, 8 nM template, 0-40 nM QT45, 0/8 nM 5TU+t1.5, 200 nM each triplet, 0/200 nM pppAUUGAU hexamer, 0.01% Tween 20, 0.4 mM $MgCl_2$, 0.6 mM KCl, 1 mM CHES-KOH, pH 9, acid-heat-cycled once to reset all RNA:RNA interactions, incubated for 27 days at -7 °C frozen. (**B**) Diagram of the effect of increasing the QT45 (+) strand concentration relative to (-). As the (+) strand increases, more (-) strands are hybridized to their reverse complement, making the template inaccessible for polymerization by QT45, and even by 5TU (in A).

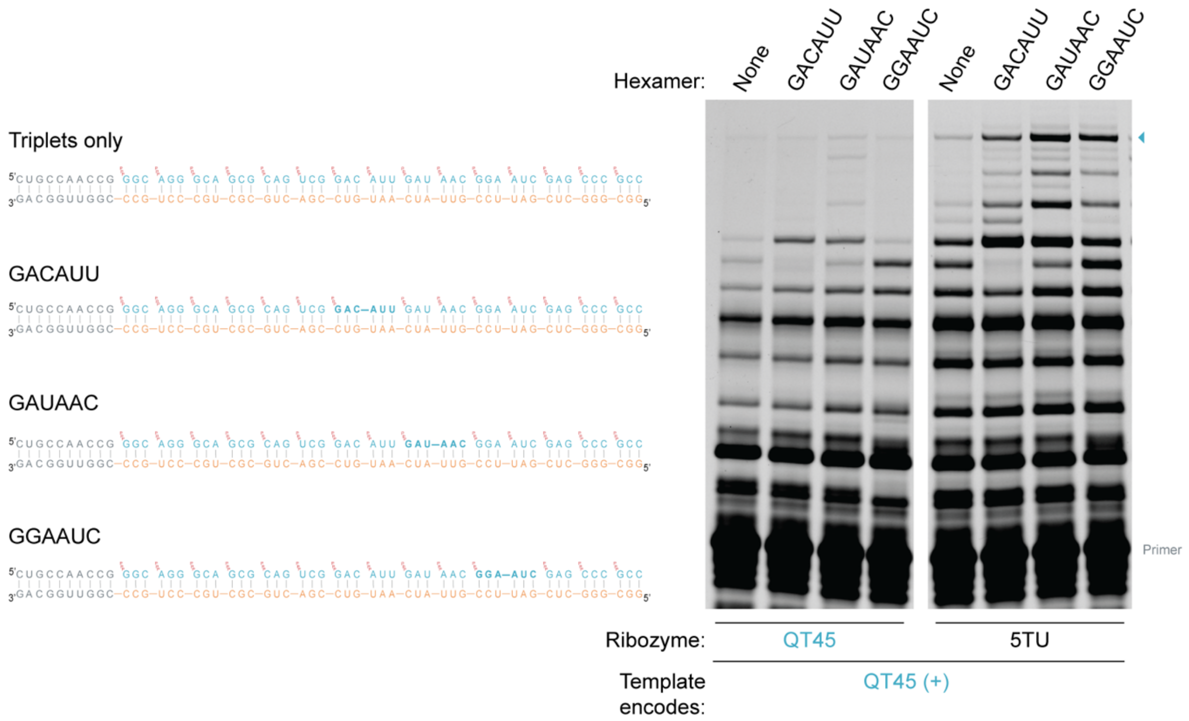

Fig. S30. Scan of individual hexameric substrates for (+) strand synthesis.
QT45-catalyzed synthesis of itself (QT45 (+)) with defined triplets and a hexamer compared with the same reaction supplemented with 5TU as an additional catalyst. Full-length is indicated by the triangle in teal. Reactions were performed using a mix of triplet substrates supplemented with a single pre-formed hexamer at various positions. Reaction conditions: 20 nM primer BCy3P10, 8 nM template, 16 nM QT45, 100 nM each triplet and hexamer, 0.01% Tween 20, 0.4 mM $MgCl_2$, 0.6 mM KCl, 1 mM CHES-KOH, pH 9, acid-heat-cycled once, incubated for 28 days at -7 °C frozen.

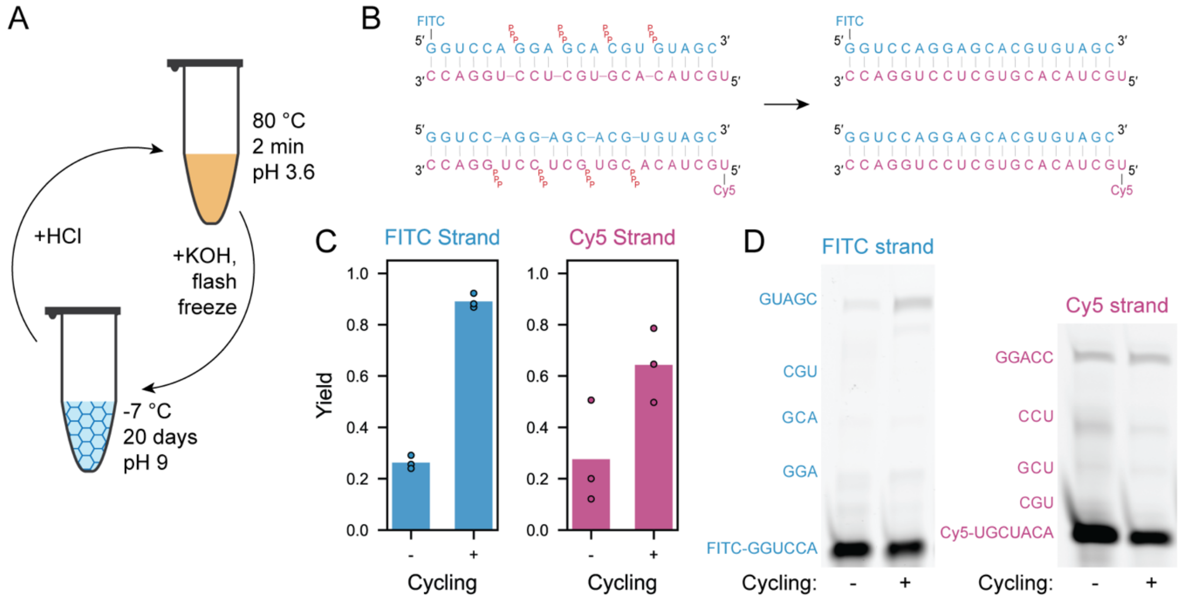

Fig. S31. Replication of a short RNA duplex sequence.

(**A**) Schematic of the conditions used for iterative RNA replication (as in (*32*)). The solution is acidified to pH 3.6 using HCl and heated to 80 °C for 2 minutes in order to separate double-stranded RNA. The solution is then neutralized and brought to pH 9 with KOH, then quickly flash frozen. Reactions are then incubated for 20 days at -7 °C, conditions conducive to RNA-catalyzed RNA polymerization. (**B**) Two short complementary RNA strands (shown in cyan and magenta) are used as templates for RNA replication, with the substrates (triphosphorylated triplets, triphosphorylated pentamer, fluorescently labelled primers) displayed hybridized to their template. (**C**) Comparison of replication of the templates and substrates shown in (B) with or without the cycling protocol shown in (A) for using QT45 ribozyme for polymerization. Average synthesis yields are shown as bar charts, with individual datapoints plotted. (**D**) Representative gels of the reactions quantified for figure (C). Reaction conditions: 1 nM plusrep3, 1 nM minusrep3, 100 nM each triplet, 20 nM each primer/pentamer, 40 nM QT45, 1 mM CHES-KOH pH 9, 0.01% Tween 20, 0.3 mM KCl, 0.4 mM $MgCl_2$, -7 °C frozen, 20 days. Cycling protocol described in detail in Supplementary Materials and Methods point 6, and its steps visualized in the diagram in (A).

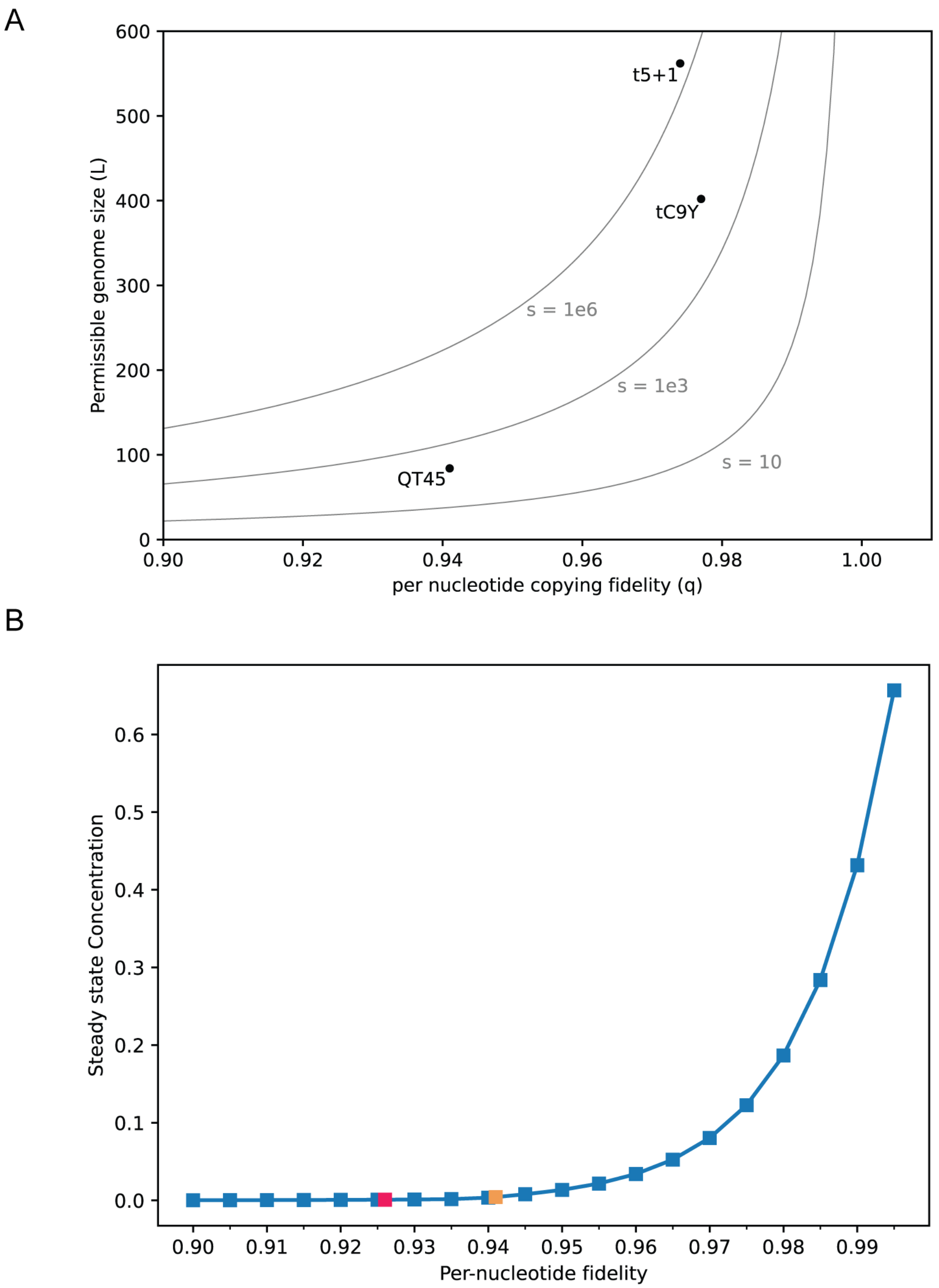

Fig. S32. Eigen error threshold.
(**A**) Theoretical relationship between ribozyme size in nucleotides (*L)* and the selective driving force *(s)* needed to maintain it at observed fidelities of replication *(q)*. *L* was calculated using the Eigen error threshold equation: $L = -log(s)/log(q)$ (*6*, *9*). (*s*) is the selective superiority of the master sequence, which corresponds to the replication rate advantage of the master sequence over its mutants exhibiting a putative shared slower replication rate, and (*q*) being the per nucleotide average replication accuracy. Per nucleotide average fidelity was used for t5+1 and QT45, rather than per trinucleotide, in order to compare more easily with the existing literature. Three different values of selective superiority (*s*) are shown as representative examples, as the selective superiority in a self-replication context is unknown. QT45, tC9Y and t5+1 ribozyme are displayed based on

their genome length. This includes the length of both (+) and (-) strand, excluding the first triplet in each strand's synthesis i.e. 84 for QT45, 402 for tC9Y and 568 for t5+1. The fidelity values used are 0.941 for QT45, this manuscript, 0.977 for tC9Y, measured in (*76*), and 0.974 for t5+1, measured in (*13*). QT45 only requires a selective superiority higher than 166 to overcome the error threshold, tC9Y would need a selective superiority higher than 1.1×10^4, and t5+1 would need a selective superiority higher than 2.6×10^6 to persist. (**B**) Fine-grained numerical simulations of the error threshold for ribozyme maintenance using observed QT45 length, fitness landscape and fidelities. These directly simulate the consequence of different global error rates in a quasispecies of polymerase mutants over which QT45 is assigned experimentally derived selective superiorities. Only the QT45 master sequence is present at the start; shown are the steady state functional concentrations of the quasispecies of active variants (Hamming distance (HD) <=2 from QT45, replication rate >= 0.8) emerging after modelling self-replication after 500 time steps (in arbitrary time units). Relative replication rates of these HD <= 2 sequences are directly taken from data in Figure 2. Individuals with missing data, as well as all HD > 2 mutants, are assigned a relative replication rate of 0.01. Per-nucleotide fidelity is provided as an average with uniform distribution of errors across nucleotides. Quasispecies concentrations from fidelity values from 90% to 99.5% (tested in steps of 0.5) are shown in blue, and from fidelities of hammerhead synthesis and QT45(-) strand synthesis are shown in red and orange respectively.

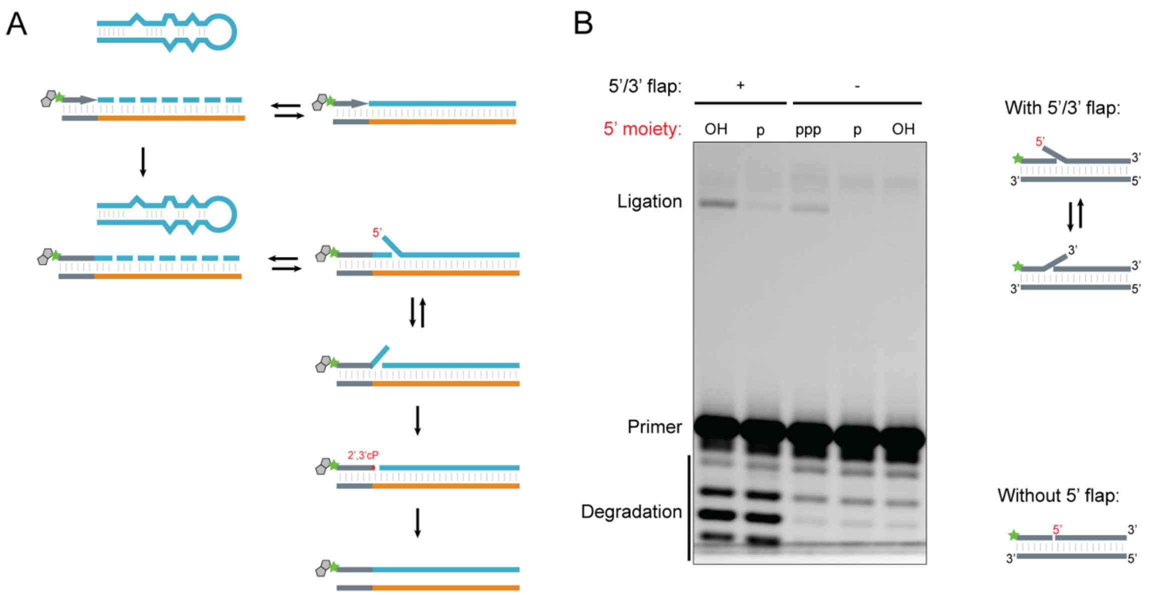

Fig. S33. Recombination between partial self-synthesis products and the ribozyme.
(**A**) Model for the recombination between partial self-synthesis products and the ribozyme used for self-synthesis. After incorporation of 1+ triplets the ribozyme can hybridize to the template, leading to a 5′-end flap. This can interchange with the partially extended product, leading to a 3′-end flap. Degradation of this flap leads to the formation of a 2′3′-cyclic phosphate, which can then lead to the templated ligation to the ribozyme used for synthesis that is hybridized to the template (this type of degradation followed by recombination was previously observed in (*51*)). 5′-end phosphorylation can reduce this side reaction, as now two separate degradation events need to occur for ligation to occur. (**B**) Model system for the study of recombination. The presence of a 5′/3′ flap leads to a ligation product that migrates like the product of nonenzymatic ligation to a 5′ triphosphorylated control oligo (a phenomenon previously described in (*77*)). Presence of a monophosphate at the 5′ reduces the extent of recombination. In the absence of the 5′/3′ flap no ligation is observed. Reaction conditions: 0.5 µM primer F10, 0.5 µM template tempF10Ltest1, 0.5 µM substrate (pppLtest1 or pLtest1 or ccgpLtest1 or pccgpLtest1), 0.01% Tween 20, 50 mM $MgCl_2$, 50 mM CHES-KOH, pH 9, incubated for 16 hours at 37 °C and then 7 days at -7 °C frozen.

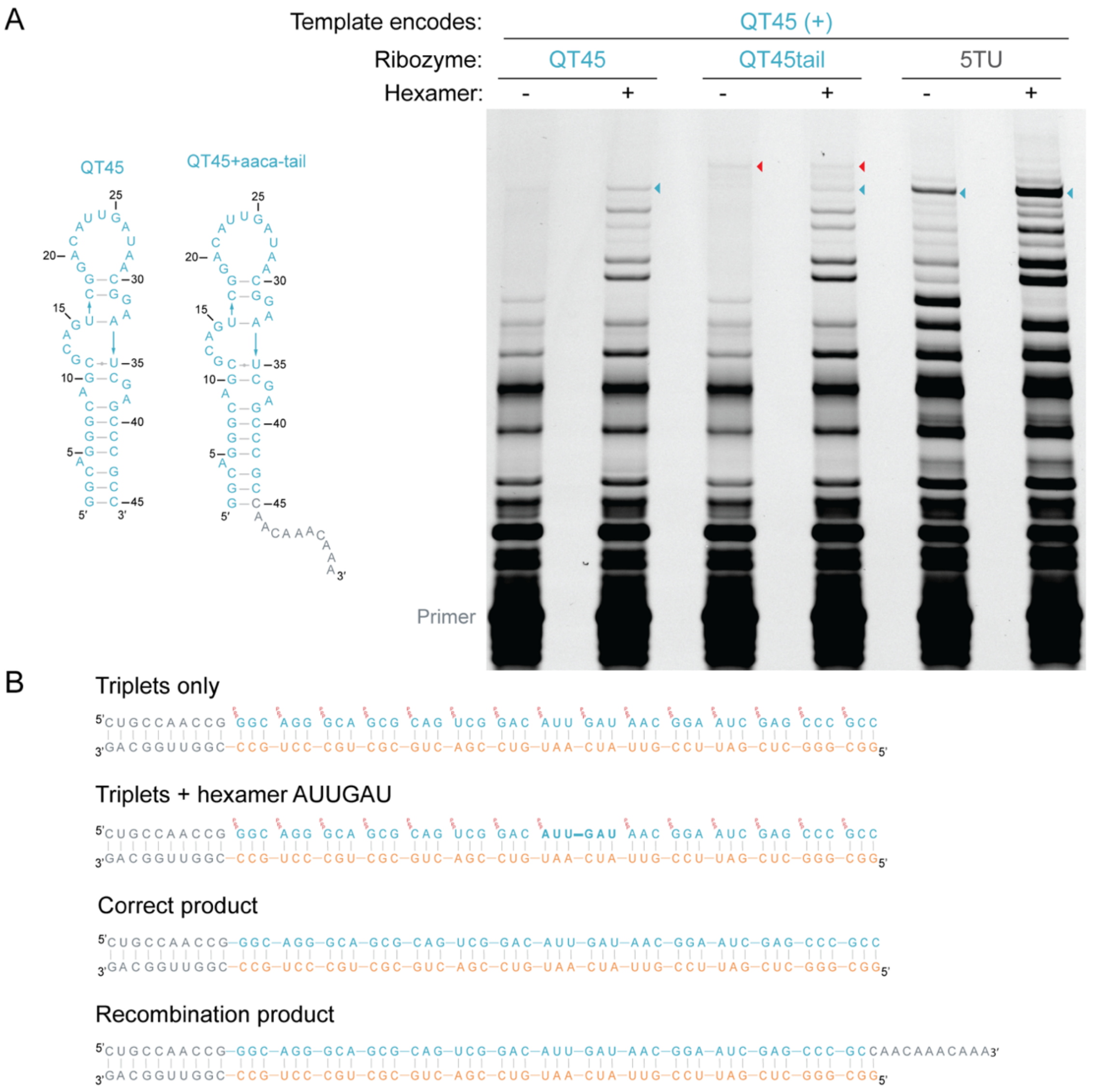

Fig. S34. QT-ribozyme-catalyzed synthesis of itself.

(**A**) Synthesis of QT45(+) by either QT45, QT45 with an added 3′-tail, or 5TU. Diagrams of the QT45 and QT45-tail variants used for this experiment are shown on the left, with nucleotides in gray indicating DNA modification. A DNA modification was introduced in the 3′-tail nucleotides to prevent adapter ligation and distinguish synthetic from recombined products via size mobility and sequencing. Reactions were carried out using a mix of triplet substrates with or without the aid of one pre-formed hexamer. Full-length is indicated by a teal triangle, incorrect recombination product that is larger and hence migrates with slower mobility is indicated by a red triangle. All sequencing reactions of QT45 (+) strand synthesis described in Fig. 4 were run with these variants to ensure discrimination. (**B**) Diagrams indicate the reactions without and with the hexamer substrate, the correct synthesis product, as well as the potential recombination product. Reaction conditions: 20 nM primer BCy3P10, 8 nM template, 16 nM QT45, 100 nM each triplet and hexamer, 0.01% Tween 20, 0.4 mM $MgCl_2$, 0.6 mM KCl, 1 mM CHES-KOH, pH 9, acid-heat-cycled once, incubated for 30 days at -7 °C frozen.

Table S1. ***De novo*** **selection conditions.**
*N20 from R5_1L and R5_2L were mixed and carried forward as 'N20'. N30 and N40 from R5_2L were mixed and carried forwards as "Mix". Error prone PCR was carried out on all libraries before carrying out round 6. ** Libraries were monomeric from round 8 onwards. "Mix" library from previous round was gel purified and its two, originally, N30 and N40 libraries were separated. These were respectively mixed with the N30 and N40 libraries from in the input to round 8. Reaction conditions: 50 nM each library/primer/template in round 1, 20 nM in later rounds.

Round number	DNA used (pmoles)	RNA used (pmoles)	Substrate	Template/primer	Time	% ligated
R1	~10	1000	Single ligation	tempF6F10<br>BTCy3P10	73h	0.1
R2	~10	100	Single ligation	tempF6F10<br>BTCy3P10	15.5h	0.02
R3	~6	100	Single ligation	tempF6F10<br>BTCy3P10	13h	0.03
R4_1L	na	20	Single ligation	tempF6F10<br>BTCy3P10	15h	0.05
R4_2L	na	20	1 triplet (GCG)	temp6FnewP10GCG<br>BCy3newP10	15h	na
R5_1L	na	20	Single ligation	tempF6F10<br>BTCy3P10	16h	0.94% (N20), 3.26% (N30), 2.25% (N40)
R5_2L	na	20	1 triplet (GCG)	temp6FnewP10GCG<br>BCy3newP10	16h	2.47% (N20), 0.15% (N30), 0.28% (N40)
R6*	na	20	1 triplet (GCG)	temp6FnewP10GCG<br>BCy3newP10	15h	0.03% (N20), 0.02% (N30), 0.04% (N40), 0.27% (Mix)
R7	na	20	1 triplet (GCG)	temp6FnewP10GCG<br>BCy3newP10	16h	0.2% (N20), 0.03% (N30), 0.27% (N40), 0.54% (Mix)
R8**	na	20	3 triplets (GCG)	temp6FP10gaGCG3<br>BCy3P10GA	16h	na
R9	na	20	3 triplets (3ACC)	temp6FP10gaACC3<br>BCy3P10GA	14h	na
R10	na	20	3 triplets (3ACC)	temp6FP10gaACC3<br>BCy3P10GA	17h	na
R11	na	20	3 triplets (3ACC)	temp6FP10gaACC3<br>BCy3P10GA	2h	na

Table S2. Reselection conditions

Round number	RNA used (pmoles)	Substrate	Template/primer	Time
R12	20000	3 triplets (GCG)	temp6FP10gaGCG3 BCy3P10ga	20 h
R13	200	3 triplets (ACC)	t6FP103ACC BCy3P10	16 h
R14	50	3 triplets (UGC)	t6FP103UGC BCy3P10	16 h
R15	20	12 triplets (CUA)	t6FP1012CUA BCy3P10	41 h
R16	20	12 triplets (CUA)	t6FP1012CUA BCy3P10	42 h
R17	10	6 triplets (CUU)	t6FnewP10CUU6 BCy3newP10	22 h
R18	20	6 triplets (AUA)	t6FnewP10AUA6 BCy3newP10	46 h

Table S3. Double mutants with reciprocal sign epistasis.

Only double mutants with statistically significant positive reciprocal sign epistasis are shown (false discovery rate < 5%). Double mutants are ranked by epistasis value from highest to lowest. Genotype notation indicates the two constituent point mutations (e.g., U16G A34C represents mutations U16G and A34C in the same sequence). Fitness values are log-transformed enrichment relative to wild type. Epistasis values represent the deviation from expected additive effects of constituent single mutations.

Genotype	Fitness	Epistasis
U16G A34C	0.57	13.47
C11U U35C	0.36	8.14
G7C C40G	0.01	7.77
C8G G39C	0.75	4.30
G7U C40A	0.85	2.82
C8G C40A	0.52	2.43

Table S4. Double mutants with significant and large (>8) positive epistasis.
Only double mutants with statistically significant positive epistasis above 8.0 are shown (false discovery rate < 5%). Double mutants are ranked by epistasis value from highest to lowest. See Table 3 for definitions.

Genotype	Fitness	Epistasis
U16G A34C	0.57	13.47
G10U C36G	-5.96	10.43
G10C C36G	-5.80	10.41
U27A G31C	-9.97	10.13
U27G U35G	-10.47	10.09
A14C A20C	-9.07	9.58
G18C U27A	-10.00	9.58
A20C A22C	-9.68	9.52
U35G C36G	-9.20	9.46
A20U A22C	-7.93	9.37
U27A U35A	-10.55	9.27
C17G U27G	-10.58	9.21
U27G A29C	-10.68	9.17
U16A A34U	-0.45	9.06
G32U U35G	-9.18	9.01
A20C C21A	0.01	8.96
A34U U35G	-9.29	8.96
G18C G32C	-9.10	8.81
C17G C30G	-9.76	8.68
A14C G18C	-9.32	8.64
A14C U27G	-11.04	8.64
G18C C30G	-8.95	8.63
G19C A22C	-10.35	8.62
C17G G31C	-10.00	8.60
A20C G31C	-9.96	8.47
U27A A34U	-10.51	8.46
A14C G32U	-8.91	8.40
U27A C36A	-10.58	8.39
G18C G31C	-9.40	8.34
C17G G32C	-10.43	8.34
C21A A22C	-1.25	8.30
C17G A29U	-10.04	8.28
A14C G31U	-9.53	8.26
A14C C30G	-10.12	8.20

C30G G32C	-10.07	8.19
U27G G31C	-11.27	8.17
C11U U35C	0.36	8.14
G31C A34U	-9.01	8.13
U27A G37C	-9.89	8.13
A20C G31U	-9.63	8.12
G18C U27G	-10.83	8.10
G18C U35G	-10.76	8.09
A22U U23A	-2.75	8.09
G18C A20C	-9.83	8.09

Table S5. Fitness and epistasis values for base pair-retaining and base pair-breaking double mutations.

Canonical base-pairing positions were identified using the algorithm described in SI Note 1 and are ranked by the average epistasis of base pair-retaining double mutants. Fitness values, epistasis values, and statistical significance are provided for each mutation type. The non-canonical C11-U35 base pair is included at the end. See Table S3 for definitions.

Position	Type	Mutation	Epistasis	Fitness	Base Pair Change
		G10A C36U	7.55	-4.51	G-C → A-U
		G10U C36A	7.95	-8.02	G-C → U-A
		G10U C36G	10.43	-5.96	G-C → U-G
	Retaining	G10C C36G	10.41	-5.80	G-C → C-G
10-36	Breaking	G10A C36A	4.80	-10.68	G-C → A-A
		U16A A34U	9.06	-0.45	U-A → A-U
		U16C A34G	-0.09	-9.69	U-A → C-G
		U16G A34U	6.94	-7.66	U-A → G-U
	Retaining	U16G A34C	13.47	0.57	U-A → G-C
		U16A A34C	5.31	-2.50	U-A → A-C
		U16A A34G	-0.37	-6.47	U-A → A-G
		U16C A34U	3.32	-9.69	U-A → C-U
16-34	Breaking	U16C A34C	1.56	-9.74	U-A → C-C
		G18A C30U	0.16	-6.81	G-C → A-U
		G18U C30A	4.79	-10.29	G-C → U-A
		G18U C30G	5.58	-10.42	G-C → U-G
	Retaining	G18C C30G	8.63	-8.95	G-C → C-G
		G18A C30A	4.21	-10.82	G-C → A-A
		G18A C30G	4.31	-11.64	G-C → A-G
		G18U C30U	0.47	-6.56	G-C → U-U
		G18C C30A	6.65	-10.00	G-C → C-A
18-30	Breaking	G18C C30U	0.46	-8.14	G-C → C-U
		C8A G39U	3.93	-0.16	C-G → A-U
		C8U G39A	2.86	1.22	C-G → U-A
		C8G G39U	3.75	-0.24	C-G → G-U
	Retaining	C8G G39C	4.30	0.75	C-G → G-C
		C8A G39A	1.74	-0.98	C-G → A-A
		C8A G39C	3.16	-0.48	C-G → A-C
		C8U G39U	-1.70	-4.70	C-G → U-U
		C8U G39C	-0.42	-2.99	C-G → U-C
8-39	Breaking	C8G G39A	1.43	-1.20	C-G → G-A
		G7A C40U	0.59	0.34	G-C → A-U
7-40	Retaining	G7U C40A	2.82	0.85	G-C → U-A

		G7U C40G	3.57	-0.51	G-C → U-G
		G7C C40G	7.77	0.01	G-C → C-G
		G7A C40A	0.50	-1.79	G-C → A-A
		G7A C40G	1.29	-3.10	G-C → A-G
		G7U C40U	-1.45	-1.37	G-C → U-U
		G7C C40A	3.53	-2.12	G-C → C-A
	Breaking	G7C C40U	0.08	-3.53	G-C → C-U
		G6A C41U	-0.38	0.76	G-C → A-U
		G6U C41A	-0.39	0.67	G-C → U-A
		G6U C41G	4.62	1.01	G-C → U-G
	Retaining	G6C C41G	4.73	0.83	G-C → C-G
		G6A C41A	0.47	1.19	G-C → A-A
		G6A C41G	2.36	-1.57	G-C → A-G
		G6U C41U	-0.91	0.57	G-C → U-U
		G6C C41A	-0.78	-0.01	G-C → C-A
6-41	Breaking	G6C C41U	-1.74	-0.57	G-C → C-U
		C11G U35C	1.07	-2.78	C-U → G-C
		C11U U35C	8.14	0.36	C-U → U-C
		C11U U35A	4.15	-9.41	C-U → U-A
	Retaining	C11U U35G	4.07	-10.87	C-U → U-G
		C11A U35A	6.43	-4.80	C-U → A-A
		C11A U35C	4.97	-0.48	C-U → A-C
		C11A U35G	2.56	-10.05	C-U → A-G
		C11G U35A	0.74	-8.89	C-U → G-A
11-35	Breaking	C11G U35G	3.88	-7.13	C-U → G-G

Table S6. Oligonucleotide sequences.
Oligonucleotide sequences are collated below. RNA is colored in orange; DNA is in black. Any modification is annotated with the supplier's specific code. 'GP' describes in house PAGE purified oligonucleotides, 'RNE' describes QIAGEN RNEasy purification, if not stated the sequences were used as supplied. 'IVT' describes T7 *in vitro* transcribed RNA. Oligonucleotides used for fitness landscape analysis are in table S7 and S8.

Application	Name	Source, purification	Sequence (5′-3′)	Notes
General fill-in/PCR	5T7	Sigma	GATCGATCTCGCCCGCGAAATTAATACGACTCACTATA	
	HDVrt	Sigma	CTTCTCCCTTAGCCTACCGAAGTAGCCCAGGTCGGACCGCGAGGAGGTGGAGATGCCATGCCGACCC	
Homodimer selection libraries and library generation oligos	pTLT	IDT	/5Phos/GGACAGTCAGGCAGT	
	fGG17	IDT	/5Phos/CAAAACAAACAAACAGG	
	ULTc2dN40	IDT, GP	/5Phos/TTGTTTGTTGGACAGTCAGGCAG/ideoxyU/NNNNNNNNNNNNNNNNNNNNNNNNNNNNNNNNNNNNNNNNCCTGTTTGTTTGTTTTG	
	ULTc2dN30	IDT, GP	/5Phos/TTGTTTGTTGGACAGTCAGGCAG/ideoxyU/NNNNNNNNNNNNNNNNNNNNNNNNNNNNNNCCTGTTTGTTTGTTTTG	
	ULTc2dN20	IDT, GP	/5Phos/TTGTTTGTTGGACAGTCAGGCAG/ideoxyU/NNNNNNNNNNNNNNNNNNNNCCTGTTTGTTTGTTTTG	
	5T76FfGG	IDT	GATCGATCTCGCCCGCGAAATTAATACGACTCACTATAGGTCCAAACAAACAACAAAACAAACAAACAGG	

	bio5T76FfGG	IDT	/5Biosg/GATCGATCTCGCCCGCGAAATTAATACGACTCACTATAGGTCCAAACAAACAACAAAACAAACAAACAGG	
	5T76FfGGLaaca15cagg	IDT	GATCGATCTCGCCCGCGAAATTAATACGACTCACTATAGGTCCAAACAAACAAACAAACAAACAAACAAACAAACAAACAAACAAACAACAAAACAAACAGG	Longer version of 5T76FfGG for 15AACA linker
Reselection libraries	1-30-sp24		CAGGCAGTAAGCAGTGCGTTTTTACGTTAATTGTTCACCTGTTTGTTTGTTTTGTTGTTTGTTTGGACC	Underlined nt were spiked at 24%
	2-30-sp24		GGACAGTCAGGCAGTTACTCGTTAGGTACTTCTTAATTTTTCGCCCCTGTTTGTTTGTTTTGTTGTTTGTTTGGACC	Underlined nt were spiked at 24%
	1-40-sp24		CAGGCAGTCTCTCGAGTCCGTTATCTATTTCCGCCTGCGCTGAGATGCCTGTTTGTTTGTTTTGTTGTTTGTTTGGACC	Underlined nt were spiked at 24%
Primers and templates used in selection	BTCy3P10	IDT, GP	/5BiotinTEG//iCy3/CUGCCAACCG	
	BCy3P10	IDT, GP	/5Biosg//iCy3/CUGCCAACCG	
	BCy3P10GA	IDT, GP	/5Biosg//iCy3/CUGCCAACCGGA	
	BCy3newP10	IDT, GP	/5BiosG//iCy3/CGCACUCAGG	
	temp6FF10	IDT, GP	UGGACCCGGUUGGCAG/3SpC3/	
	temp6FnewP10GCG	IDT, GP	UGGACCCGCCCUGAGUGCG/3SpC3/	
	t6FP10gaGCG3	IDT, GP	UGGACCCGCCGCCGCUCCGGUUGGCAG/3SpC3/	

	temp6FP10gaACC3	IDT, GP	UGGACCGGUGGUGGUUCCGGUUGGCAG/3SpC3/	
	temp6FP10ACC3	IDT, GP	UGGACCGGUGGUGGUCGGUUGGCAG/3SpC3/	
	temp6FP103ugc	IDT, GP	UGGACCgcagcagcaCGGUUGGCAG/3SpC3/	
	temp6FP1012cua	IDT, GP	UGGACCuaguaguaguaguaguaguaguaguaguaguaguagCGGUUGGCAG	
	temp6FnewP10CUU6	IDT, GP	UGGACCaagaagaagaagaagaagCCUGAGUGCG/3SpC3/	
	temp6FnewP10AUA6	IDT, GP	UGGACCuauuauuauuauuauuauCCUGAGUGCG/3SpC3/	
Recovery primers	HDVrec	IDT	GATGCCATGCCGACCC	Used for recovery of rounds 12-18 alongside round-specific primers described below
	pP106Frec	IDT	/5Phos/CTGCCAACCGGGTCCA	Used for recovery of rounds R1, R2, R3, R4_1L alongside pTLT
	pnewP10gcFrec	IDT	/5Phos/CGCACTCAGGGC	Used for recovery of rounds R4_2L, R5_1L, R5_2L, R6, R7 alongside pTLT
	pP10GAgcgFrec	IDT	/5Phos/TGCCAACCGGAgc	Used for recovery of rounds R8, R12
	pP10GAaccFrec	IDT	/5Phos/CTGCCAACCGGAac	Used for recovery of rounds R9, R10, R11
	P10accFrec	IDT	CTGCCAACCGacc	Used for recovery of R13
	P10ugcFrec	IDT	CTGCCAACCGtgc	Used for recovery of R14
	forceGG	Sigma	AACAAACAACAAAACAAACAAACAGG	Used for recovery in R15, R16, R17, R18
Adapters used in recovery	AdeHDVlig		Ap-pGGGTCGGCATGGCATC/3SpC3/	Prepared via adenylation as in method 1.4,

				starting from HDVlig
	HDVlig	IDT, GP	/5Phos/GGGTCGGCAT GGCATC/3SpC3/	
Regiospecificity assay	FBAP9_3p	ChemGens, GP	[FAM][biotin]CUACAA CCG[3′-phosphate]	Marker for cleaved reaction
	FBAP9_35ACC	ChemGens, GP	[FAM][biotin]CUACAA CCGACC	Standard for correct 3'-5' bond
	FBAP9_25ACC	ChemGens, GP	[FAM][biotin]CUACAA CCG[2'-5']ACC	Standard for incorrect 2'-5' bond
	FBAP9	ChemGens, GP	[FAM][biotin]CUACAA CCG	Generated via T4 PNK dephosphorylation of FITCreg3P
	t6FAP9ACCCUG	IDT, GP	UGGACCCAGGGUCG GUUGUAG/3SpC3/	Template for regiospecificity assay.
	hoCUG	ChemGenes	hoCUG	Monophosphorylated chemically synthesized triplet
Clones from de novo selection	8_R11_20 (1-30)	IVT, GP	GGUCCAAACAAACA ACAAAACAAACAAA **CAGGUGAACAAUUA ACGUAAAAAACGCA CUGCUUACUGCCUG**	Underlined nucleotides hybridize to the template. Bold nucleotides indicate residues likely to fold into ribozyme.
	8_R11_20_R	IDT	CAGGCAGTAAGCAG TGCGTTTTTTACGTT AATTGTTCACCTGTT TGTTTGTTTTGTTGTT TGTTTGGACC	Used together with 5T76FfGG to generate template for transcription of 8_R11_r0
	0_R8_30 (2-30)	IVT, GP	GGUCCAAACAAACA ACAAAACAAACAAA **CAGGGGCGAAAAA UUAAGAAGUACCUA ACGAGUAACUGCCU GACUGUCC**	Underlined nucleotides hybridize to the template. Bold nucleotides indicate residues likely to fold into ribozyme.
	0_R8_30_R	IDT	GGACAGTCAGGCAG TTACTCGTTAGGTAC TTCTTAATTTTTCGC	Used together with 5T76FfGG to generate

			CCCTGTTTGTTTGTTTTGTTGTTTGTTTGGACC	template for transcription of 1_R11_40
	1_R11_40 (1-40)	IVT, GP	GGUCCAAACAAACAACAAAACAAACAAA**CAGGCAUCUCAGCGCAGGCGGAAAUAGAUAACGGACUCGAGAGACUGCCUG**	Underlined nucleotides hybridize to the template. Bold nucleotides indicate residues likely to fold into ribozyme.
	1_R11_40_R	IDT	CAGGCAGTCTCTCGAGTCCGTTATCTATTTCCGCCTGCGCTGAGATGCCTGTTTGTTTGTTTTGTTGTTTGTTTGGACC	Used together with 5T76FfGG to generate template for transcription of 1_R11_40
Ribozymes used in this study	5TU	IVT,GP	GGAUCUUCUCGAUCUAACAAAAAGACAAAUCUGCCACAAAGCUUGAGAGCAUCUUCGGAUGCAGAGGCGGCAGCCUUCGGUGGCGCGAUAGCGCCAACGUUCUCAACUAUGACACGCAAAACGCGUGCUCCGUUGAAUGGAGUUUAUCAUG	
	5T7_5TUF	Sigma	GATCGATCTCGCCCGCGAAATTAATACGACTCACTATAGGATCTTCTCGATCTAACAAAAAGACAAATCTGCCACAAAGCTTGAGAGCATCTTCGGATG	Used with 5TUR for fill-in and generate a template for 5TU
	5TUR	Sigma	CATGATAAACTCCATTCAACGGAGCACGCGTTTTGCGTGTCATAGTTGAGAACGTTGGCGCTATCGCGCCACCGAAGGCTGCCGCCTCTGCATCCGAAGATGCTCTCAAGCTTTGTGGCA	
	T1.5	IVT, GP	GACCAAUCUGCCCUCAGAGCCCGAGAACAUCUUCGGAUGCAG	

			AGGAGGCAGGCUUC GGUGGCGCGAUAGC GCCAACGUCCUCAA CCUCCAAUGCAUCC CACCACAUGAUGAG CCUGAAGAGCCUUG GUUUUUUUG	
	5T7_t1.5F	Sigma	GATCGATCTCGCCCG CGAAATTAATACGA CTCACTATAGACCAA TCTGCCCTCAGAGCC CGAGAACATCTTCGG ATGCAGAGGAG	Used with t1.5R to fill-in and generate a template for t1.5
	t1.5R	Sigma	CAAAAAAACCAAGG CTCTTCAGGCTCATC ATGTGGTGGGATGC ATTGGAGGTTGAGG ACGTTGGCGCTATCG CGCCACCGAAGCCT GCCTCCTCTGCATCC G	
	QT51	IDT, GP	ACAGGCAUCUCAGC GCAGUCGGACAUUG AUAAUGGAAUCGAG AGAGUCUGU	
	QT45	IDT, GP	GGCAGGGCAGCGCA GUCGGACAUUGAUA ACGGAAUCGAGCCC GCC	
	pQT45	IDT,GP	/5Phos/GGCAGGGCAG CGCAGUCGGACAUU GAUAACGGAAUCGA GCCCGCC	5′-end monophosphate used to reduce recombination to partial synthesis products.
	FITC-QT45	IDT, GP	GCAGGGCAGCGCAG UCGGACAUUGAUAA CGGAAUCGAGCCCG CC/36-FAM/	Used to assess degradation/half-life
	QT40	IDT, GP	GGGGCAGCGCAGUC GGACAUUGAUAACG GAAUCGAGCCCC	
	QT35	IDT, GP	GGCAGCGCAGUCGG AAUUGAUAAUGGAA UCGAGCC	
	QT45aaca		GGCAGGGCAGCGCA GUCGGACAUUGAUA	3′-end DNA tail use to distinguish

			ACGGAAUCGAGCCCGCC AACAAACAAA	synthetic products from QT ribozyme used for synthesis.
	QT39	IVT, GP	GGGGCAGCGCAGUCGGACAUUGAUAACGGAAUCGAGCCC	
	0_51_r7trim	IDT, GP	GGUCCAAACAAACAACAAAACAAACAAA**GGCCAGCGCAGTCGGACATTGATAATGGAATCGAGGCC**	Underlined nucleotides hybridize to the template. Bold nucleotides indicate residues likely to fold into ribozyme.
	0_qt51_r7_trim_R	IDT	GGCCTCGATTCCATTATCAATGTCCGACTGCGCTGGCCTTTGTTTGTTTTGTTGTTTGTT	Used with 5T76FnocaGG (below) to prepare the template for transcription of 0_51_r7trim
	5T76FnocaGG	IDT	GATCGATCTCGCCCGCGAAATTAATACGACTCACTATAGGTCCAAACAAACAACAAAACAAACAAAGG	
	QT51_6F5L	IVT, GP	GGUCCAAACAACAAAACAAACAA**ACAGGCAUCUCAGCGCAGUCGGACAUUGAUAAUGGAAUCGAGAGAGUCUGU**	Underlined nucleotides hybridize to the template. Bold nucleotides indicate residues likely to fold into ribozyme
	QT51_6F5L_F		GATCGATCTCGCCCGCGAAATTAATACGACTCACTATAGGTCCAACAAAACAAACAAACAGGCATCTCAGC	Used with QT51_6F5L_R for fill-in to generate the template for transcription of QT51_6F5L
	QT51_6F5L_R		ACAGACTCTCTCGATTCCATTATCAATGTCCGACTGCGCTGAGATGCCTGTTTGTTTGTTTTG	

Primers used for RNA-catalyzed RNA synthesis	F10	IDT, GP	/56-FAM/CUGCCAACCG	
Templates used for RNA-catalyzed RNA synthesis of Fig. 1 and 2	tP10CGU20	IVT, GP	GGACGACGACGACGACGACGACGACGACGACGACGACGACGACGACGACGACGACGACGACGACGCGGUUGGCAG	Primer binding site underlined.
	tP10_20cgu(tx)	IDT	CTGCCAACCGCGTCGTCGTCGTCGTCGTCGTCGTCGTCGTCGTCGTCGTCGTCGTCGTCGTCGTCGTCGTGGTATAGTGAGTCGTATTAATTTCGC	Used with 5T7 to prepare the template for transcription of tP10CGU20.
	P10CGU20comp	IVT, RNE	GGCUGCCAACCGCGUCGUCGUCGUCGUCGUCGUCGUCGUCGUCGUCGUCGUCGUCGUCGUCGUCGUCGUCGUCGU	Used as "competing oligo" to improve resolution of tP10CGU20 copying reaction on a denaturing PAGE.
	P10CGU20comp(tx)	IDT	ACGACGACGACGACGACGACGACGACGACGACGACGACGACGACGACGACGACGACGACGACGCGGTTGGCAGCCTATAGTGAGTCGTATTAATTTCGCGGGC	Used with 5T7 to prepare the template for transcription of P10CGU20comp.
	tP10CGU14	IDT, GP	ACGACGACGACGACGACGACGACGACGACGACGACGACGACGACGCGGUUGGCAG	Primer binding site underlined.
	P10CGU14comp	IVT, RNE	GGCUGCCAACCGCGUCGUCGUCGUCGUCGUCGUCGUCGUCGUCGUCGUCGUCGUCGU	Used as "competing oligo" to improve resolution of tP10CGU14 copying reaction on a denaturing PAGE.
	P10CGU14comp(tx)	IDT	ACGACGACGACGACGACGACGACGACGA	Used with 5T7 to prepare the

			CGACGACGACGACG CGGTTGGCAGCCTAT AGTGAGTCGTATTAA TTTCGCGGGCGAGAT CGA	template for transcription of P10CGU14comp
	t6FP10mix	IDT, GP	UGGACCUAUGCGUU CGAAGGUCGCCGGU UGGCAG/3SpC3/	
	rc_of_t6FP10mix	IDT, GP	CUGCCAACCGGCGA CCUUCGAACGCAUA GGUCCA	Used as “competing oligo” to improve resolution of t6FP10mix copying reaction on a denaturing PAGE.
Primers and templates encoding fragments of the t5 polymerase, used for synthesis with mono- di- and tri-nucleotide mixes	Fdelta7	IDT, GP	/56-FAM/GCGAUAG	
	tdelta	IDT, GP	AUGUCAUGGUUGAG AACGUUGGCGCUAU CGC	
	(tx)tdelta_comp	IDT	ATGTCATGGTTGAGA ACGTTGGCGCTATCG CTATAGTGAGTCGTA TTAATTTC	Competing oligo used to visualize delta synthesis reactions
	Fgamma7	IDT, GP	/56-FAM/GGAUGCA	
	tgamma	IDT, GP	GCCACCGAAGGCUG CCGCCUCUGCAUCC	
	(tx)tgamma_comp	IDT	GCCACCGAAGGCTG CCGCCTCTGCATCCT ATAGTGAGTCGTATT AATTTC	Competing oligo used to visualize gamma synthesis reactions
Stem-loop containing templates copying	t_4S	IDT, GP	GAGAAGGAGUCCGC UCCACACCGGUUGG CAG	4 base-pairs stem template
	t_6S	IDT, GP	GAGAAGGAGUGUCC GCACUCCACACCGG UUGGCAG	6 base-pairs stem template
	t_8S	IDT, GP	GAGAAGGAGUGUGU CCGCACACUCCACA CCGGUUGGCAG	8 base-pairs stem template
	F9	IDT, GP	/56-FAM/CUGCCAACC	Primer used for stem-loop containing templates copying

Hammerhead Seq0-HH synthesis and activity assay	tP10Lte_seq0HH	IDT, GP	ACACGCUUCGCCGCAUUUCGUGCCGUAGCACUCAUCAGUACGUCCGGUUGGCAG	Template for seq0-HH synthesis
	Fsub-seq0HH	IDT,GP	/56-FAM/CGCAUAUACGUC	Substrate used for assaying seq0-HH
	5T7kyleF		GATCGATCTCGCCCGCGAAATTAATACGACTCACTATAGGATTCACTGCGATAGAGTC	Primer to amplify seq0-HH for subsequent transcription
	kyleP10LteHDV_seq0HH	IDT	GATGCCATGCCGACCCACACGCTTCGCCGCATTTCGTGCCGTAGCACTCATCAGTACGTCCGGTTGGCAGGACTCTATCGCAGTGAATCC	Oligo to generate WT seq0-HH by *in vitro* transcription
Mini hammerhead synthesis and activity assay	tP10HHz	IDT, GP	GCGCCUCAUCAGUCGAGCCGGUUGGCAG	Template used for mini hammerhead synthesis in Fig. 3
	Fsubuhl	IDT, GP	/56-FAM/GCGCCGAAACACCGUGUCUCGAGC	Substrate for mini hammerhead cleavage
	DNAtP10_Euhlp1	IDT	GCGCCTCATCAGTCGAGCCGGTTGGCAG	Template used for TGK synthesis of mini hammerhead marker
	A647BP10	IDT	/5Alex647N//iBiodT/CUGCCAACCG	Primer used for synthesis of mini hammerhead used for cleavage assay
	bioCy3KyleP10	IDT, GP	/5BiosG//iCy3/GGATTCACTGCGATAGAGTCCUGCCAACCG	Primer used for synthesis of mini hammerhead used for sequencing
Templates used for (+) and (-) strand synthesis	t4psP10QT45	IDT, GP	GGCGGGCUCGAUUCCGUUAUCAAUGUCCGACUGCGCUGCCCUGCCCGGUUGGCAG	Template for (+) strand synthesis in Fig. 4
	t4msP10QT45	IDT, GP	GCAGGGCAGCGCAGUCGGACAUUGAUAACGGAAUCGAGCCCGCCCCGGUUGGCAG	Template for (-) strand synthesis in Fig. 4
Hexamer synthesis	pppGACAUU	IVT, GP	pppGACAUU	

	GACAUU_tx	Sigma	AATGTCTATAGTGAGTCGTATTAATTTCGCGGGCGAGATCGATC	
	pppGAUAAC	IVT, GP	pppGAUAAC	
	GAUAAC_tx	Sigma	GTTATCTATAGTGAGTCGTATTAATTTCGCGGGCGAGATCGATC	
	pppGGAAUC	IVT, GP	pppGGAAUC	
	GGAAUC_tx	Sigma	GATTCCTATAGTGAGTCGTATTAATTTCGCGGGCGAGATCGATC	
	pppAUUGAU	Chemgenes, GP	pppAUUGAU	
Analysis of recombination	pppLtest1	IVT, GP	pppGCGAAGCGUGU	
	(tx)Ltest1	IDT	ACACGCTTCGCTATAGTGAGTCGTATTAATTTCGCGGGCGAGATCGATC	Fill-in with 5T7 to generate template to transcribe pppLtest1
	Ltest	IDT, GP	GCGAAGCGUGU	
	pLtest	IDT, GP	/5Phos/GCGAAGCGUGU	
	AppLtest	GP	App-GCGAAGCGUGU	Adenylated pLtest
	ccgpLtest1	IDT, GP	CCGGCGAAGCGUGU	
	pccgpLtest1	IDT, GP	/5Phos/CCGGCGAAGCGUGU	
Polymerisation using mononucleotides	tP10_44G_AGUA	IDT, GP	AUGACCCCCGGUUGGCAG	Template encoding 4 Gs, used for mononucleotides extensions
	(tx)comptP10_44G_AGUA	IDT	ATGACCCCCGGTTGGCAGccTATAGTGAGTCGTATTAATTTCGCGGGCGAGATCGATC	Competing oligo for improved visualisation of the above synthesis
	tP10_44C_AGUA	IDT, GP	AUGAGGGGCGGUUGGCAG	Template encoding 4 Cs, used for mononucleotides extensions

	(tx)comptP10_44C_AGUA	IDT	ATGAGGGGCGGTTGGCAGccTATAGTGAGTCGTATTAATTTCGCGGGCGAGATCGATC	Competing oligo for improved visualisation of the above synthesis
	tP10_44A_AGUA	IDT, GP	AUGAUUUUCGGUUGGCAG	Template encoding 4 As, used for mononucleotides extensions
	(tx)comptP10_44A_AGUA	IDT	ATGATTTTCGGTTGGCAGccTATAGTGAGTCGTATTAATTTCGCGGGCGAGATCGATC	Competing oligo for improved visualisation of the above synthesis
	tP10_44U_AGUA	IDT, GP	AUGAAAAACGGUUGGCAG	Template encoding 4 Us, used for mononucleotides extensions
	(tx)comptP10_44U_AGUA	IDT	ATGAAAAACGGTTGGCAGccTATAGTGAGTCGTATTAATTTCGCGGGCGAGATCGATC	Competing oligo for improved visualisation of the above synthesis
Synthesis of 3 GCA comparing triphosphate and adenylate activation	BCy3nnP12	IDT, GP	/5Biosg//iCy3/CGCACGAGUCUC	Used for synthesis using AppGCA
	temp6FP12nn3GCA	IDT, GP	UGGACCUGCUGCUGCGAGACUCGUGCG/3SpC3/	Used as template for AppGCA/pppGCA synthesis
Deoxynucleotide substitution scanning	F10_d-1	Sigma, GP	/56-FAM/CUGCCAACC[dG]	
	F10_d-2	Sigma, GP	/56-FAM/CUGCCAAC[dC]G	
	F10_d-3	Sigma, GP	/56-FAM/CUGCCAA[dC]CG	
	F10_d-4	Sigma, GP	/56-FAM/CUGCCA[dA]CCG	
	F10_d-5	Sigma, GP	/56-FAM/CUGCC[dA]ACCG	

	tF10Ltest_d-1	Sigma, GP	ACACGCUUCGC[dC]G GUUGGCAG	
	tF10Ltest_d-2	Sigma, GP	ACACGCUUCGCC[dG] GUUGGCAG	
	tF10Ltest_d-3	Sigma, GP	ACACGCUUCGCCG[d G]UUGGCAG	
	tF10Ltest_d-4	Sigma, GP	ACACGCUUCGCCGG[dU]UGGCAG	
	tF10Ltest_d-5	Sigma, GP	ACACGCUUCGCCGG U[dU]GGCAG	
	tF10Ltest_d+1	Sigma, GP	ACACGCUUCG[dC]CG GUUGGCAG	
	tF10Ltest_d+2	Sigma, GP	ACACGCUUC[dG]CCG GUUGGCAG	
	tF10Ltest_d+3	Sigma, GP	ACACGCUU[dC]GCCG GUUGGCAG	
	tF10Ltest_d+4	Sigma, GP	ACACGCU[dU]CGCCG GUUGGCAG	
	tF10Ltest_d+5	Sigma, GP	ACACGC[dU]UCGCCG GUUGGCAG	
	tF10Ltest_GCGt GCA	Sigma, GP	ACACGCUUUGCCGG UUGGCAG	
	DSsub	IVT, GP	pppGCGAAGCGUGU	
	tDSsub	Sigma	ACACGCTTCGCTATA GTGAGTCGTATTAAT TTCGC	Fill-in with 5T7 to generate template for DSsub
	DSsub_GCGtGC A	IVT, GP	pppGCAAAGCGUGU	
	tDSsub_GCGtGC A	Sigma	ACACGCTTTGCTATA GTGAGTCGTATTAAT TTCGC	Fill-in with 5T7 to generate template for DSub_GCGtGCA
	DSsub_GCGtGC A_d+1	Chemg enes, GP	ppp[dG]CAAAGCGUG U	
	DSsub_GCGtGC A_d+2	Chemg enes, GP	pppG[dC]AAAGCGUG U	
	DSsub_GCGtGC A_d+3	Chemg enes, GP	pppGC[dA]AAGCGUG U	
	DSsub_GCGtGC A_d+4		pppGCA[dA]AGCGUG U	Prepared via splinted ligation of pppGCA to DSsub_minGCA_

				d+4 using tTripletLigation_3spc3 as the template. T4 RNA ligase 2 (NEB) was used for ligation according to the manufacturer's protocol, with incubation overnight at 5 °C.
	DSsub_minGCA_d+4	IDT, GP	/5Phos/[dA]AGCGUGU	
	DSsub_GCGtGCA_d+5		pppGCAA[dA]GCGUGU	Prepared via splinted ligation of pppGCA to DSsub_minGCA_d+5 using tTripletLigation_3spc3 as the template. T4 RNA ligase 2 (NEB) was used for ligation according to the manufacturer's protocol, with incubation overnight at 5 °C.
	DSsub_minGCA_d+5	IDT, GP	/5Phos/A[dA]GCGUGU	
	tTripletLigation_3spc3	IDT, GP	AACAAACAAACAACACGCTTTGC/3SpC3	Splint used for the generation of DSsub_minGCA_d+4 and DSsub_minGCA_d+5
Replication assay	Rep3plus	IDT	GGUCCAGGAGCACGUGUAGC	
	Rep3minus	IDT	UGCUACACGUGCUCCUGGACC	
	FITCrep	IDT	FITC-GGUCCA	
	Cy5rep	IDT	Cy5-UGCUACA	

	pppGUAGC	IVT, GP	pppGUAGC	
	(tx)GUAGC	Sigma	GCTACTATAGTGAGTCGTATTAATTTCGCGGGCGAGATCGATC	Fill-in with 5T7 to generate template for transcription of GUAGC
	pppGGACC	IVT, GP	pppGGACC	
	(tx)GGACC	Sigma	GGTCCTATAGTGAGTCGTATTAATTTCGCGGGCGAGATCGATC	Fill-in with 5T7 to generate template for transcription of GGACC
Sequencing primers	ggP10	IDT	GGCTGCCAACCG	Used for RT-PCR of (+) and (-) strand synthesis products together with HDVrec
	kyleF	IDT	GGATTCACTGCGATAGAGTC	Used for RT-PCR of seq0-HH synthesis product.
	P7XGGP10	IDT	CAAGCAGAAGACGGCATACGAGATGTGACTGGAGTTCAGACGTGTGCTCTTCCGATCTNNNXXXXXXGGGCTGCCAACCG	Primer used to incorporate P7 sequencing adapter to RT-PCR products of ggP10. XXXXXX region corresponds to either of the following barcodes: ATCACG, CGATGT, TTAGGC, TGACCA, ACAGTG, GCCAAT, CAGATC, ACTTGA, GATCAG, TAGCTT, GGCTAC, CTTGTA, AGTCAA, AGTTCC

	P7XkyleF	IDT	CAAGCAGAAGACGG CATACGAGATGTGA CTGGAGTTCAGACGT GTGCTCTTCCGATCT NNNXXXXXXGGATT CACTGCGATAGAGTC	Primer used to incorporate P7 sequencing adapter to RT-PCR products of kyleF. XXXXXX uses barcodes similarly to P7XGGP10.
	P5XHDVba	IDT	AATGATACGGCGAC CACCGAGATCTACAC TCTTTCCCTACACGA CGCTCTTCCGATCTN NNXXXXXXGATGCC ATGCCGACCC	Primer used to incorporate P5 sequencing adapter to RT-PCR products of HDVrec. XXXXXX uses barcodes similarly to P7XGGP10.

Table S7. Oligonucleotide sequences used for fitness landscape analysis.
Oligonucleotide sequences used for fitness landscape of Figure 2 are collated below. RNA is colored in orange, DNA is in black. Any modification is annotated with the supplier's specific code. 'GP' describes in house PAGE purified oligonucleotides, 'RNE' describes QIAGEN RNEasy purification, if not stated the sequences were used as supplied. 'IVT' describes T7 *in vitro* transcribed RNA.

Application	Name	Source, purification	Sequence	Notes
Sequencing primers	p71forceG_2024	IDT	CAAGCAGAAGACGGCATACGAGATGTGACTGGAGTTCAGACGTGTGCTCTTCCGATCTNNNGATACTAACAAACAACAAAACAAACAAACAG	Used for all libraries
	p51HDVba_2021	IDT	AATGATACGGCGACCACCGAGATCTACACTCTTTCCCTACACGACGCTCTTCCGATCTNNNATCACGGATGCCATGCCGACCC	Nodel input library
	p52HDVba_2021	IDT	AATGATACGGCGACCACCGAGATCTACACTCTTTCCCTACACGACGCTCTTCCGATCTNNNCGATGTGATGCCATGCCGACCC	Del input library
	p53HDVba_2021	IDT	AATGATACGGCGACCACCGAGATCTACACTCTTTCCCTACACGACGCTCTTCCGATCTNNNTTAGGCGATGCCATGCCGACCC	Nodel output A
	p54HDVba_2021	IDT	AATGATACGGCGACCACCGAGATCTACACTCTTTCCCTACACGACGCTCTTCCGATCTNNNTGACCAGATGCCATGCCGACCC	Nodel output B
	p55HDVba_2021	IDT	AATGATACGGCGACCACCGAGATCTACACTCTTTCCCTACACGACGCTCTTCCGATCTNNNACAGTGGATGCCATGCCGACCC	Nodel output C
	p56HDVba_2021	IDT	AATGATACGGCGACCACCGAGATCTACACTCTTTCCCTACACGACGCTCTTCCGATCTNNNGCCAATGATGCCATGCCGACCC	Del output A

	p57HDVba_2021	IDT	AATGATACGGCGACCACCGAGATCTACACTCTTTCCCTACACGACGCTCTTCCGATCTNNNCAGATCGATGCCATGCCGACCC	Del output B
	p58HDVba_2021	IDT	AATGATACGGCGACCACCGAGATCTACACTCTTTCCCTACACGACGCTCTTCCGATCTNNNACTTGAGATGCCATGCCGACCC	Del output C
Reverse fill-in oligonucleotides for making nodel libraries	C45N_sp12_QT45mo10	IDT	/5Phos/GATGCCATGCCGACCCNGCGGGCTCGATTCCGTTATCAATGTCCGACTGCGCTGCCCTGCCTGTTTGTTTGTTTTGTTG	Underlined nt were spiked at 12%
	C44N_sp12_QT45mo10	IDT	/5Phos/GATGCCATGCCGACCCGNCGGGCTCGATTCCGTTATCAATGTCCGACTGCGCTGCCCTGCCTGTTTGTTTGTTTTGTTG	Underlined nt were spiked at 12%
	G43N_sp12_QT45mo10	IDT	/5Phos/GATGCCATGCCGACCCGGNGGGCTCGATTCCGTTATCAATGTCCGACTGCGCTGCCCTGCCTGTTTGTTTGTTTTGTTG	Underlined nt were spiked at 12%
	C42N_sp12_QT45mo10	IDT	/5Phos/GATGCCATGCCGACCCGGCNGGCTCGATTCCGTTATCAATGTCCGACTGCGCTGCCCTGCCTGTTTGTTTGTTTTGTTG	Underlined nt were spiked at 12%
	C41N_sp12_QT45mo10	IDT	/5Phos/GATGCCATGCCGACCCGGCGNGCTCGATTCCGTTATCAATGTCCGACTGCGCTGCCCTGCCTGTTTGTTTGTTTTGTTG	Underlined nt were spiked at 12%
	C40N_sp12_QT45mo10	IDT	/5Phos/GATGCCATGCCGACCCGGCGGNCTCGATTCCGTTATCAATGTCCGACTGCGCTGCCCTGCCTGTTTGTTTGTTTTGTTG	Underlined nt were spiked at 12%
	G39N_sp12_QT45mo10	IDT	/5Phos/GATGCCATGCCGACCCGGCGGGNTCGATTCCGTTATCAATGTCCGACTGCGCTGCCCTGCCTGTTTGTTTGTTTTGTTG	Underlined nt were spiked at 12%

	A38N_sp12_QT45mo10	IDT	/5Phos/GATGCCATGCCGACCCGGCGGGCNCGATTCCGTTATCAATGTCCGACTGCGCTGCCCTGCCTGTTTGTTTGTTTTGTTG	Underlined nt were spiked at 12%
	G37N_sp12_QT45mo10	IDT	/5Phos/GATGCCATGCCGACCCGGCGGGCTNGATTCCGTTATCAATGTCCGACTGCGCTGCCCTGCCTGTTTGTTTGTTTTGTTG	Underlined nt were spiked at 12%
	C36N_sp12_QT45mo10	IDT	/5Phos/GATGCCATGCCGACCCGGCGGGCTCNATTCCGTTATCAATGTCCGACTGCGCTGCCCTGCCTGTTTGTTTGTTTTGTTG	Underlined nt were spiked at 12%
	U35N_sp12_QT45mo10	IDT	/5Phos/GATGCCATGCCGACCCGGCGGGCTCGNTTCCGTTATCAATGTCCGACTGCGCTGCCCTGCCTGTTTGTTTGTTTTGTTG	Underlined nt were spiked at 12%
	A34N_sp12_QT45mo10	IDT	/5Phos/GATGCCATGCCGACCCGGCGGGCTCGANTCCGTTATCAATGTCCGACTGCGCTGCCCTGCCTGTTTGTTTGTTTTGTTG	Underlined nt were spiked at 12%
	A33N_sp12_QT45mo10	IDT	/5Phos/GATGCCATGCCGACCCGGCGGGCTCGATNCCGTTATCAATGTCCGACTGCGCTGCCCTGCCTGTTTGTTTGTTTTGTTG	Underlined nt were spiked at 12%
	G32N_sp12_QT45mo10	IDT	/5Phos/GATGCCATGCCGACCCGGCGGGCTCGATTNCGTTATCAATGTCCGACTGCGCTGCCCTGCCTGTTTGTTTGTTTTGTTG	Underlined nt were spiked at 12%
	G31N_sp12_QT45mo10	IDT	/5Phos/GATGCCATGCCGACCCGGCGGGCTCGATTCNGTTATCAATGTCCGACTGCGCTGCCCTGCCTGTTTGTTTGTTTTGTTG	Underlined nt were spiked at 12%
	C30N_sp12_QT45mo10	IDT	/5Phos/GATGCCATGCCGACCCGGCGGGCTCGATTCCNTTATCAATGTCCGACTGCGCTGCCCTGCCTGTTTGTTTGTTTTGTTG	Underlined nt were spiked at 12%

	A29N_sp12_QT45mo10	IDT	/5Phos/GATGCCATGCCGACCCGGCGGGCTCGATTCCGNTATCAATGTCCGACTGCGCTGCCCTGCCTGTTTGTTTGTTTTGTTG	Underlined nt were spiked at 12%
	A28N_sp12_QT45mo10	IDT	/5Phos/GATGCCATGCCGACCCGGCGGGCTCGATTCCGTNATCAATGTCCGACTGCGCTGCCCTGCCTGTTTGTTTGTTTTGTTG	Underlined nt were spiked at 12%
	U27N_sp12_QT45mo10	IDT	/5Phos/GATGCCATGCCGACCCGGCGGGCTCGATTCCGTTNTCAATGTCCGACTGCGCTGCCCTGCCTGTTTGTTTGTTTTGTTG	Underlined nt were spiked at 12%
	A26N_sp12_QT45mo10	IDT	/5Phos/GATGCCATGCCGACCCGGCGGGCTCGATTCCGTTANCAATGTCCGACTGCGCTGCCCTGCCTGTTTGTTTGTTTTGTTG	Underlined nt were spiked at 12%
	G25N_sp12_QT45mo10	IDT	/5Phos/GATGCCATGCCGACCCGGCGGGCTCGATTCCGTTATNAATGTCCGACTGCGCTGCCCTGCCTGTTTGTTTGTTTTGTTG	Underlined nt were spiked at 12%
	U24N_sp12_QT45mo10	IDT	/5Phos/GATGCCATGCCGACCCGGCGGGCTCGATTCCGTTATCNATGTCCGACTGCGCTGCCCTGCCTGTTTGTTTGTTTTGTTG	Underlined nt were spiked at 12%
	U23N_sp12_QT45mo10	IDT	/5Phos/GATGCCATGCCGACCCGGCGGGCTCGATTCCGTTATCANTGTCCGACTGCGCTGCCCTGCCTGTTTGTTTGTTTTGTTG	Underlined nt were spiked at 12%
	A22N_sp12_QT45mo10	IDT	/5Phos/GATGCCATGCCGACCCGGCGGGCTCGATTCCGTTATCAANGTCCGACTGCGCTGCCCTGCCTGTTTGTTTGTTTTGTTG	Underlined nt were spiked at 12%
	C21N_sp12_QT45mo10	IDT	/5Phos/GATGCCATGCCGACCCGGCGGGCTCGATTCCGTTATCAATNTCCGACTGCGCTGCCCTGCCTGTTTGTTTGTTTTGTTG	Underlined nt were spiked at 12%

	A20N_sp12_QT45mo10	IDT	/5Phos/GATGCCATGCCGACCCGGCGGGCTCGATTCCGTTATCAATGNCCGACTGCGCTGCCCTGCCTGTTTGTTTGTTTTGTTG	Underlined nt were spiked at 12%
	G19N_sp12_QT45mo10	IDT	/5Phos/GATGCCATGCCGACCCGGCGGGCTCGATTCCGTTATCAATGTNCGACTGCGCTGCCCTGCCTGTTTGTTTGTTTTGTTG	Underlined nt were spiked at 12%
	G18N_sp12_QT45mo10	IDT	/5Phos/GATGCCATGCCGACCCGGCGGGCTCGATTCCGTTATCAATGTCNGACTGCGCTGCCCTGCCTGTTTGTTTGTTTTGTTG	Underlined nt were spiked at 12%
	C17N_sp12_QT45mo10	IDT	/5Phos/GATGCCATGCCGACCCGGCGGGCTCGATTCCGTTATCAATGTCCNACTGCGCTGCCCTGCCTGTTTGTTTGTTTTGTTG	Underlined nt were spiked at 12%
	U16N_sp12_QT45mo10	IDT	/5Phos/GATGCCATGCCGACCCGGCGGGCTCGATTCCGTTATCAATGTCCGNCTGCGCTGCCCTGCCTGTTTGTTTGTTTTGTTG	Underlined nt were spiked at 12%
	G15N_sp12_QT45mo10	IDT	/5Phos/GATGCCATGCCGACCCGGCGGGCTCGATTCCGTTATCAATGTCCGANTGCGCTGCCCTGCCTGTTTGTTTGTTTTGTTG	Underlined nt were spiked at 12%
	A14N_sp12_QT45mo10	IDT	/5Phos/GATGCCATGCCGACCCGGCGGGCTCGATTCCGTTATCAATGTCCGACNGCGCTGCCCTGCCTGTTTGTTTGTTTTGTTG	Underlined nt were spiked at 12%
	C13N_sp12_QT45mo10	IDT	/5Phos/GATGCCATGCCGACCCGGCGGGCTCGATTCCGTTATCAATGTCCGACTNCGCTGCCCTGCCTGTTTGTTTGTTTTGTTG	Underlined nt were spiked at 12%
	G12N_sp12_QT45mo10	IDT	/5Phos/GATGCCATGCCGACCCGGCGGGCTCGATTCCGTTATCAATGTCCGACTGNGCTGCCCTGCCTGTTTGTTTGTTTTGTTG	Underlined nt were spiked at 12%

	C11N_sp12_QT45mo10	IDT	/5Phos/GATGCCATGCCGACCCGGCGGGCTCGATTCCGTTATCAATGTCCGACTGCNCTGCCCTGCCTGTTTGTTTGTTTTGTTG	Underlined nt were spiked at 12%
	G10N_sp12_QT45mo10	IDT	/5Phos/GATGCCATGCCGACCCGGCGGGCTCGATTCCGTTATCAATGTCCGACTGCGNTGCCCTGCCTGTTTGTTTGTTTTGTTG	Underlined nt were spiked at 12%
	A9N_sp12_QT45mo10	IDT	/5Phos/GATGCCATGCCGACCCGGCGGGCTCGATTCCGTTATCAATGTCCGACTGCGCNGCCCTGCCTGTTTGTTTGTTTTGTTG	Underlined nt were spiked at 12%
	C8N_sp12_QT45mo10	IDT	/5Phos/GATGCCATGCCGACCCGGCGGGCTCGATTCCGTTATCAATGTCCGACTGCGCTNCCCTGCCTGTTTGTTTGTTTTGTTG	Underlined nt were spiked at 12%
	G7N_sp12_QT45mo10	IDT	/5Phos/GATGCCATGCCGACCCGGCGGGCTCGATTCCGTTATCAATGTCCGACTGCGCTGNCCTGCCTGTTTGTTTGTTTTGTTG	Underlined nt were spiked at 12%
	G6N_sp12_QT45mo10	IDT	/5Phos/GATGCCATGCCGACCCGGCGGGCTCGATTCCGTTATCAATGTCCGACTGCGCTGCNCTGCCTGTTTGTTTGTTTTGTTG	Underlined nt were spiked at 12%
	G5N_sp12_QT45mo10	IDT	/5Phos/GATGCCATGCCGACCCGGCGGGCTCGATTCCGTTATCAATGTCCGACTGCGCTGCCNTGCCTGTTTGTTTGTTTTGTTG	Underlined nt were spiked at 12%
	A4N_sp12_QT45mo10	IDT	/5Phos/GATGCCATGCCGACCCGGCGGGCTCGATTCCGTTATCAATGTCCGACTGCGCTGCCCNGCCTGTTTGTTTGTTTTGTTG	Underlined nt were spiked at 12%
	C3N_sp12_QT45mo10	IDT	/5Phos/GATGCCATGCCGACCCGGCGGGCTCGATTCCGTTATCAATGTCCGACTGCGCTGCCCTNCCTGTTTGTTTGTTTTGTTG	Underlined nt were spiked at 12%

	G2N_sp12_QT45mo10	IDT	/5Phos/GATGCCATGCCGACCCGGCGGGCTCGATTCCGTTATCAATGTCCGACTGCGCTGCCCTGNCTGTTTGTTTGTTTTGTTG	Underlined nt were spiked at 12%
Reverse fill-in oligonucleotides for making del libraries	G2d_sp12_QT45mo10	IDT	/5Phos/GATGCCATGCCGACCCGGCGGGCTCGATTCCGTTATCAATGTCCGACTGCGCTGCCCTGCTGTTTGTTTGTTTTGTTG	Underlined nt were spiked at 12%
	C3d_sp12_QT45mo10	IDT	/5Phos/GATGCCATGCCGACCCGGCGGGCTCGATTCCGTTATCAATGTCCGACTGCGCTGCCCTCCTGTTTGTTTGTTTTGTTG	Underlined nt were spiked at 12%
	A4d_sp12_QT45mo10	IDT	/5Phos/GATGCCATGCCGACCCGGCGGGCTCGATTCCGTTATCAATGTCCGACTGCGCTGCCCGCCTGTTTGTTTGTTTTGTTG	Underlined nt were spiked at 12%
	G7d_sp12_QT45mo10	IDT	/5Phos/GATGCCATGCCGACCCGGCGGGCTCGATTCCGTTATCAATGTCCGACTGCGCTGCCTGCCTGTTTGTTTGTTTTGTTG	Underlined nt were spiked at 12%
	C8d_sp12_QT45mo10	IDT	/5Phos/GATGCCATGCCGACCCGGCGGGCTCGATTCCGTTATCAATGTCCGACTGCGCTCCCTGCCTGTTTGTTTGTTTTGTTG	Underlined nt were spiked at 12%
	A9d_sp12_QT45mo10	IDT	/5Phos/GATGCCATGCCGACCCGGCGGGCTCGATTCCGTTATCAATGTCCGACTGCGCGCCCTGCCTGTTTGTTTGTTTTGTTG	Underlined nt were spiked at 12%
	G10d_sp12_QT45mo10	IDT	/5Phos/GATGCCATGCCGACCCGGCGGGCTCGATTCCGTTATCAATGTCCGACTGCGTGCCCTGCCTGTTTGTTTGTTTTGTTG	Underlined nt were spiked at 12%
	C11d_sp12_QT45mo10	IDT	/5Phos/GATGCCATGCCGACCCGGCGGGCTCGATTCCGTTATCAATGTCCGACTGCCTGCCCTGCCTGTTTGTTTGTTTTGTTG	Underlined nt were spiked at 12%

	G12d_sp12_QT45mo10	IDT	/5Phos/GATGCCATGCCGACCC<u>GGCGGGCTCGATTCCGTTATCAATGTCCGACTGGCTGCCCTGC</u>CTGTTTGTTTGTTTTGTTG	Underlined nt were spiked at 12%
	C13d_sp12_QT45mo10	IDT	/5Phos/GATGCCATGCCGACCC<u>GGCGGGCTCGATTCCGTTATCAATGTCCGACTCGCTGCCCTGC</u>CTGTTTGTTTGTTTTGTTG	Underlined nt were spiked at 12%
	A14d_sp12_QT45mo10	IDT	/5Phos/GATGCCATGCCGACCC<u>GGCGGGCTCGATTCCGTTATCAATGTCCGACGCGCTGCCCTGC</u>CTGTTTGTTTGTTTTGTTG	Underlined nt were spiked at 12%
	G15d_sp12_QT45mo10	IDT	/5Phos/GATGCCATGCCGACCC<u>GGCGGGCTCGATTCCGTTATCAATGTCCGATGCGCTGCCCTGC</u>CTGTTTGTTTGTTTTGTTG	Underlined nt were spiked at 12%
	U16d_sp12_QT45mo10	IDT	/5Phos/GATGCCATGCCGACCC<u>GGCGGGCTCGATTCCGTTATCAATGTCCGCTGCGCTGCCCTGC</u>CTGTTTGTTTGTTTTGTTG	Underlined nt were spiked at 12%
	C17d_sp12_QT45mo10	IDT	/5Phos/GATGCCATGCCGACCC<u>GGCGGGCTCGATTCCGTTATCAATGTCCACTGCGCTGCCCTGC</u>CTGTTTGTTTGTTTTGTTG	Underlined nt were spiked at 12%
	G19d_sp12_QT45mo10	IDT	/5Phos/GATGCCATGCCGACCC<u>GGCGGGCTCGATTCCGTTATCAATGTCGACTGCGCTGCCCTGC</u>CTGTTTGTTTGTTTTGTTG	Underlined nt were spiked at 12%
	A20d_sp12_QT45mo10	IDT	/5Phos/GATGCCATGCCGACCC<u>GGCGGGCTCGATTCCGTTATCAATGCCGACTGCGCTGCCCTGC</u>CTGTTTGTTTGTTTTGTTG	Underlined nt were spiked at 12%
	C21d_sp12_QT45mo10	IDT	/5Phos/GATGCCATGCCGACCC<u>GGCGGGCTCGATTCCGTTATCAATTCCGACTGCGCTGCCCTGC</u>CTGTTTGTTTGTTTTGTTG	Underlined nt were spiked at 12%

	A22d_sp12_QT45mo10	IDT	/5Phos/GATGCCATGCCGACCCGGCGGGCTCGATTCCGTTATCAAGTCCGACTGCGCTGCCCTGCCTGTTTGTTTGTTTTGTTG	Underlined nt were spiked at 12%
	U24d_sp12_QT45mo10	IDT	/5Phos/GATGCCATGCCGACCCGGCGGGCTCGATTCCGTTATCATGTCCGACTGCGCTGCCCTGCCTGTTTGTTTGTTTTGTTG	Underlined nt were spiked at 12%
	G25d_sp12_QT45mo10	IDT	/5Phos/GATGCCATGCCGACCCGGCGGGCTCGATTCCGTTATAATGTCCGACTGCGCTGCCCTGCCTGTTTGTTTGTTTTGTTG	Underlined nt were spiked at 12%
	A26d_sp12_QT45mo10	IDT	/5Phos/GATGCCATGCCGACCCGGCGGGCTCGATTCCGTTACAATGTCCGACTGCGCTGCCCTGCCTGTTTGTTTGTTTTGTTG	Underlined nt were spiked at 12%
	U27d_sp12_QT45mo10	IDT	/5Phos/GATGCCATGCCGACCCGGCGGGCTCGATTCCGTTTCAATGTCCGACTGCGCTGCCCTGCCTGTTTGTTTGTTTTGTTG	Underlined nt were spiked at 12%
	A29d_sp12_QT45mo10	IDT	/5Phos/GATGCCATGCCGACCCGGCGGGCTCGATTCCGTATCAATGTCCGACTGCGCTGCCCTGCCTGTTTGTTTGTTTTGTTG	Underlined nt were spiked at 12%
	C30d_sp12_QT45mo10	IDT	/5Phos/GATGCCATGCCGACCCGGCGGGCTCGATTCCTTATCAATGTCCGACTGCGCTGCCCTGCCTGTTTGTTTGTTTTGTTG	Underlined nt were spiked at 12%
	G32d_sp12_QT45mo10	IDT	/5Phos/GATGCCATGCCGACCCGGCGGGCTCGATTCGTTATCAATGTCCGACTGCGCTGCCCTGCCTGTTTGTTTGTTTTGTTG	Underlined nt were spiked at 12%
	A34d_sp12_QT45mo10	IDT	/5Phos/GATGCCATGCCGACCCGGCGGGCTCGATCCGTTATCAATGTCCGACTGCGCTGCCCTGCCTGTTTGTTTGTTTTGTTG	Underlined nt were spiked at 12%

	U35d_sp12_QT45mo10	IDT	/5Phos/GATGCCATGCCGACCCGGCGGGCTCGTTCCGTTATCAATGTCCGACTGCGCTGCCCTGCCTGTTTGTTTGTTTTGTTG	Underlined nt were spiked at 12%
	C36d_sp12_QT45mo10	IDT	/5Phos/GATGCCATGCCGACCCGGCGGGCTCATTCCGTTATCAATGTCCGACTGCGCTGCCCTGCCTGTTTGTTTGTTTTGTTG	Underlined nt were spiked at 12%
	G37d_sp12_QT45mo10	IDT	/5Phos/GATGCCATGCCGACCCGGCGGGCTGATTCCGTTATCAATGTCCGACTGCGCTGCCCTGCCTGTTTGTTTGTTTTGTTG	Underlined nt were spiked at 12%
	A38d_sp12_QT45mo10	IDT	/5Phos/GATGCCATGCCGACCCGGCGGGCCGATTCCGTTATCAATGTCCGACTGCGCTGCCCTGCCTGTTTGTTTGTTTTGTTG	Underlined nt were spiked at 12%
	G39d_sp12_QT45mo10	IDT	/5Phos/GATGCCATGCCGACCCGGCGGGTCGATTCCGTTATCAATGTCCGACTGCGCTGCCCTGCCTGTTTGTTTGTTTTGTTG	Underlined nt were spiked at 12%
	C42d_sp12_QT45mo10	IDT	/5Phos/GATGCCATGCCGACCCGGCGGCTCGATTCCGTTATCAATGTCCGACTGCGCTGCCCTGCCTGTTTGTTTGTTTTGTTG	Underlined nt were spiked at 12%
	G43d_sp12_QT45mo10	IDT	/5Phos/GATGCCATGCCGACCCGGGGGCTCGATTCCGTTATCAATGTCCGACTGCGCTGCCCTGCCTGTTTGTTTGTTTTGTTG	Underlined nt were spiked at 12%
	C45d_sp12_QT45mo10	IDT	/5Phos/GATGCCATGCCGACCCGCGGGCTCGATTCCGTTATCAATGTCCGACTGCGCTGCCCTGCCTGTTTGTTTGTTTTGTTG	Underlined nt were spiked at 12%
DNA to add HDV sequence to reverse fill-in	HDVrest	IDT	/5AmMC6/CTTCTCCCTTAGCCTACCGAAGTAGCCCAGGTCGGACCGCGAGGAGGTGGA	Used to ligate to library oligonucleotides and add the HDV sequence

oligonucleotides for making libraries	HDVspl	IDT	/5AmMC6/GGGTCGGCATGGCATCTCCACCTCCTCG/3AmMO/	Used to splint the ligation of library oligonucleotides and add the HDV sequence
Forward fill-in oligonucleotide for making libraries	5T76FfG	IDT	GATCGATCTCGCCCGCGAAATTAATACGACTCACTATAGGTCCAAACAAACAACAAAACAAACAAACAG	Fill-in oligo for library preparation
Adapters used in recovery	AdeHDVlig	GP	Ap-pGGGTCGGCATGGCATC/3SpC3/	Prepared via adenylation starting from HDVlig
	HDVlig	IDT, GP	/5Phos/GGGTCGGCATGGCATC/3SpC3/	Starting material to prepare adeHDVlig
Forward recovery primer	P10UGCugFrec	IDT	CTGCCAACCGTGCTG	Used for RTPCR of output libraries
	forceG	IDT	AACAAACAACAAAACAAACAAACAG	Used for RTPCR of input libraries
Reverse recovery primer	HDVrec	IDT	GATGCCATGCCGACCC	Used for RTPCR of all output samples
Primer used in selection round	BCy3P10	IDT, GP	/5Biosg//iCy3/CUGCCAACCG	
Template used in selection round	temp6FP10UGC3	IDT, GP	UGGACCGCAGCAGCACGGUUGGCAG/3SpC3/	

Table S8. Oligonucleotide sequences used for fitness landscape analysis on multiple template sequences

Oligonucleotide sequences used for fitness landscape of Figure S16, S17, S18 are collated below. RNA is colored in orange; DNA is in black. Any modification is annotated with the supplier's specific code. 'GP' describes in house PAGE purified oligonucleotides, 'RNE' describes QIAGEN RNEasy purification, if not stated the sequences were used as supplied. 'IVT' describes T7 *in vitro* transcribed RNA.

Application	Name	Source, purification	Sequence	Notes
Sequencing primers	P71forceGG_2024	IDT	CAAGCAGAAGACGGCATACGAGATGTGACTGGAGTTCAGACGTGTGCTCTTCCGATCTNNNgatactAACAAACAACAAAACAAACAAACAGG	Fitness landscape sequencing primer for output libraries
	P72forceGG_2024	IDT	CAAGCAGAAGACGGCATACGAGATGTGACTGGAGTTCAGACGTGTGCTCTTCCGATCTNNNatcttgAACAAACAACAAAACAAACAAACAGG	Fitness landscape sequencing primer for input libraries
	P51HDVba_2021	IDT	AATGATACGGCGACCACCGAGATCTACACTCTTTCCCTACACGACGCTCTTCCGATCTNNNatcacgGATGCCATGCCGACCC	Fitness landscape sequencing primer for 3 UGC output sample
	P52HDVba_2021	IDT	AATGATACGGCGACCACCGAGATCTACACTCTTTCCCTACACGACGCTCTTCCGATCTNNNcgatgtGATGCCATGCCGACCC	Fitness landscape sequencing primer for 12 CUA output sample
	P53HDVba_2021	IDT	AATGATACGGCGACCACCGAGATCTACACTCTTTCCCTACACGACGCTCTTCCGATCTNNNttaggcGATGCCATGCCGACCC	Fitness landscape sequencing primer for 3 AUA output sample
	P512HDVba_2021	IDT	AATGATACGGCGACCACCGAGATCTACACTCTTTCCCTACACGACGCTCTTCCGATCTNNNcttgtaGATGCCATGCCGACCC	Fitness landscape sequencing primer for sp12-6L library
	P514HDVba_2021	IDT	AATGATACGGCGACCACCGAGATCTACACTCTTTCCCTACACGACGCTCTTCCGATCTNNNagttccGATGCCATGCCGACCC	Fitness landscape sequencing primer for sp12-8L library

Reverse fill-in oligonucleotides for making libraries	QT45MO10	IDT	/5Phos/GATGCCATGCCGACCCGGCGGGCTCGATTCCGTTATCAATGTCCGACTGCGCTGCCCTGCCCTGTTTGTTTGTTTTGTTG	Underlined nt were spiked at 12%
	QT45MO10_iG1_dC45	IDT	/5Phos/GATGCCATGCCGACCCGCGGGCTCGATTCCGTTATCAATGTCCGACTGCGCTGCCCTGCCCCTGTTTGTTTGTTTTGTTG	Underlined nt were spiked at 12%
	QT45MO10_dG1_iC45	IDT	/5Phos/GATGCCATGCCGACCCGGGCGGGCTCGATTCCGTTATCAATGTCCGACTGCGCTGCCCTGCCTGTTTGTTTGTTTTGTTG	Underlined nt were spiked at 12%
	QT45MO10_C21D_ins1G	IDT	/5Phos/GATGCCATGCCGACCCGGCGGGCTCGATTCCGTTATCAATTCCGACTGCGCTGCCCTGCCCCTGTTTGTTTGTTTTGTTG	Underlined nt were spiked at 12%
	QT45MO10_C21D_ins45C	IDT	/5Phos/GATGCCATGCCGACCCGGGCGGGCTCGATTCCGTTATCAATTCCGACTGCGCTGCCCTGCCCTGTTTGTTTGTTTTGTTG	Underlined nt were spiked at 12%
	QT45MO10_C21D_ins20G	IDT	/5Phos/GATGCCATGCCGACCCGGCGGGCTCGATTCCGTTATCAATTCCCGACTGCGCTGCCCTGCCCTGTTTGTTTGTTTTGTTG	Underlined nt were spiked at 12%
	QT45MO10_C21D_ins22G	IDT	/5Phos/GATGCCATGCCGACCCGGCGGGCTCGATTCCGTTATCAACTTCCGACTGCGCTGCCCTGCCCTGTTTGTTTGTTTTGTTG	Underlined nt were spiked at 12%
	QT45MO10_U23D_ins45C	IDT	/5Phos/GATGCCATGCCGACCCGGGCGGGCTCGATTCCGTTATCATGTCCGACTGCGCTGCCCTGCCCTGTTTGTTTGTTTTGTTG	Underlined nt were spiked at 12%
	QT45MO10_U23D_ins1G	IDT	/5Phos/GATGCCATGCCGACCCGGCGGGCTCGATTCCGTTATCATGTCCGACTGCGCTGCCCTGCCCCTGTTTGTTTGTTTTGTTG	Underlined nt were spiked at 12%
DNA to add HDV	HDVrest	IDT	/5AmMC6/CTTCTCCCTTAGCCTACCGAAGTAGCCC	Used to ligate to library oligos and

sequence to reverse fill-in oligonucleotides for making libraries			AGGTCGGACCGCGAGGAGGTGGA	add the HDV sequence
	HDVspl	IDT	/5AmMC6/GGGTCGGCATGGCATCTCCACCTCCTCG/3AmMO/	Used to splint the ligation of library oligos and add the HDV sequence
Forward fill-in oligonucleotide for making libraries	5T76FfGG	IDT	GATCGATCTCGCCCGCGAAATTAATACGACTCACTATAGGTCCAAACAAACAACAAAACAAACAAACAGG	Fill-in oligo for library preparation
Adapters used in recovery	AdeHDVlig	GP	Ap-pGGGTCGGCATGGCATC/3SpC3/	Prepared via adenylation starting from HDVlig
	HDVlig	IDT	/5Phos/GGGTCGGCATGGCATC/3SpC3/	Starting material to prepare adeHDVlig
Forward recovery primer	P10UGCugFrec	IDT	CTGCCAACCGTGCTG	Used for RTPCR of 3 UGC output sample
	forceGG	IDT	AACAAACAACAAAACAAACAAACAGG	Used for RTPCR of 12 CUA output sample
	newnewP12auaaFrec	IDT	CGCACGAGTCTCATAA	Used for RTPCR of 3 AUA sample
Reverse recovery primer	HDVrec	IDT	GATGCCATGCCGACCC	Used for RTPCR of all output samples
Primers used in selection round	BCy3newnewP12	IDT, GP	/5Biosg//iCy3/CGCACGAGUCUC	Primer used with 3 AUA template
	BCy3P10	IDT, GP	/5Biosg//iCy3/ CUGCCAACCG	Primer used with 12 CUA and 3 UGC templates
Templates used in selection round	temp6FP10UGC3	IDT, GP	UGGACCGCAGCAGCACGGUUGGCAG/3SpC3/	3 UGC template
	temp6FnewnewP12AUA3	IDT, GP	UGGACCUAUUAUUAUGAGACUCGUGCG/3SpC3/	3 AUA template
	temp6FP10CUA12	IDT, GP	UGGACCUAGUAGUAGUAGUAGUAGUAGUAGUAGUAGUAGUAGCGGUUGGCAG	12 CUA template
